# Insights into Electrochemical CO_2_ Reduction on Metallic and Oxidized Tin Using Grand-Canonical DFT and In Situ ATR-SEIRA Spectroscopy

**DOI:** 10.1021/acscatal.4c01290

**Published:** 2024-05-14

**Authors:** Todd N. Whittaker, Yuval Fishler, Jacob M. Clary, Paige Brimley, Adam Holewinski, Charles B. Musgrave, Carrie A. Farberow, Wilson A. Smith, Derek Vigil-Fowler

**Affiliations:** † Department of Chemical and Biological Engineering, Renewable and Sustainable Energy Institute, University of Colorado Boulder, Boulder, Colorado 80303, United States; ‡ National Renewable Energy Laboratory, Golden, Colorado 80401, United States; § Materials, Chemical, and Computational Science Directorate, National Renewable Energy Laboratory, Golden, Colorado 80401, United States; ∥ Materials Science and Engineering Program, University of Colorado Boulder, Boulder, Colorado 80303, United States; ⊥ Catalytic Carbon Transformation and Scale-Up Center, National Renewable Energy Laboratory, Golden, Colorado 80401, United States

**Keywords:** CO_2_ reduction, formic acid production, grand-canonical DFT, ATR-SEIRAS, mechanism evaluation

## Abstract

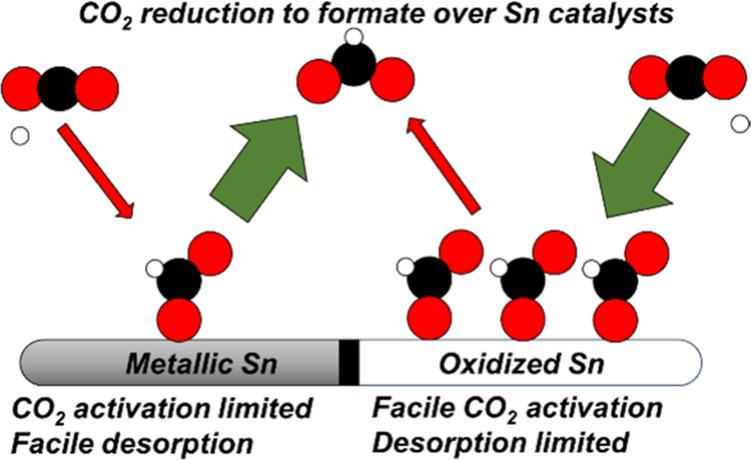

Electrochemical CO_2_ reduction (CO_2_R) to formate is an attractive carbon emissions mitigation strategy due to the existing market and attractive price for formic acid. Tin is an effective electrocatalyst for CO_2_R to formate, but the underlying reaction mechanism and whether the active phase of tin is metallic or oxidized during reduction is openly debated. In this report, we used grand-canonical density functional theory and attenuated total reflection surface-enhanced infrared absorption spectroscopy to identify differences in the vibrational signatures of surface species during CO_2_R on fully metallic and oxidized tin surfaces. Our results show that CO_2_R is feasible on both metallic and oxidized tin. We propose that the key difference between each surface termination is that CO_2_R catalyzed by metallic tin surfaces is limited by the electrochemical activation of CO_2_, whereas CO_2_R catalyzed by oxidized tin surfaces is limited by the slow reductive desorption of formate. While the exact degree of oxidation of tin surfaces during CO_2_R is unlikely to be either fully metallic or fully oxidized, this study highlights the limiting behavior of these two surfaces and lays out the key features of each that our results predict will promote rapid CO_2_R catalysis. Additionally, we highlight the power of integrating high-fidelity quantum mechanical modeling and spectroscopic measurements to elucidate intricate electrocatalytic reaction pathways.

## Introduction

Electrochemical CO_2_ reduction (CO_2_R) is a potential strategy to lessen the impact of anthropogenic climate change and has been studied extensively across a variety of electrode materials over the last few decades.^1^ Copper has received the most attention in the CO_2_R literature due to its seemingly unique ability to produce C_2+_ products such as ethylene and ethanol with appreciable Faradaic efficiency, while gold and silver have also been studied for their high selectivity to CO.^2−4^ However, a recent technoeconomic analysis showed that under 2019 market conditions, formic acid was the CO_2_R product with a production cost closest to break-even.^5^ Formic acid, which has an annual global demand of ∼800 kilotons and is used mostly as a food preservative and in the production of rubber and leather, is produced at scale via the hydrolysis of formamide, which has undesirable environmental impacts.^6^ Therefore, CO_2_R to produce formic acid is not only a promising climate solution but can potentially reduce dependence on a nonideal existing industrial process.

CO_2_R catalysts that are selective toward formate are generally characterized by having a weak affinity for CO. Most are p-block metals, and, among these, tin (Sn) is the most attractive because it has higher electrochemical stability than zinc and indium, is more environmentally benign than cadmium or lead, and is more abundant than bismuth.^7,8^ There have been many reports of using Sn-based electrocatalysts for CO_2_R, generally showing that at potentials more negative than −0.6 V_RHE_, Faradaic efficiencies of >70% toward formic acid can be achieved with state-of-the-art current densities reaching 500 mA/cm^2^.^9−12^ However, several reports have shown differing interpretations of the precise nature of the active phase of Sn during CO_2_R. Based on the Pourbaix diagram for Sn, the purely metallic phase is expected to be present at potentials more negative than −0.5 V_RHE_. However, numerous reports have invoked oxide phases of Sn as the active phase,^10,13−20^ with some going as far as to say that CO_2_R does not proceed on metallic Sn at all.^21−23^ In direct opposition to this conclusion, many other reports claim that the metallic phase is the active phase and that in situ reduction of the oxide material to a more active metallic Sn phase is responsible for the high observed CO_2_R activity and selectivity.^9,24,25^ Clearly, neither the true surface state nor the mechanism of CO_2_R on Sn-based electrocatalysts has been firmly established, and this lack of clarity has limited the development of improved catalysts and reactor systems.

In this report, we use a combined computational chemistry and in situ spectroscopic approach to examine CO_2_R on both metallic and oxidized Sn electrodes. While a number of metastable Sn phases exist between the fully metallic Sn^0^ and fully oxidized SnO_2_ phases that could be responsible for CO_2_R activity,^13,21^ we chose these end points as useful limiting cases to examine. We use grand-canonical density functional theory (GC-DFT) to investigate the CO_2_R mechanism under conditions relevant to the in situ experimental measurements. This approach poses several advantages compared to simpler methods such as the computational hydrogen electrode (CHE) method developed by Nørskov and co-workers almost 20 years ago. While the CHE is elegantly simple and has led to valuable insights into electrocatalytic mechanisms,^26^ it also suffers from key limitations, namely that all molecular geometries, including the catalyst adsorbate structures, are relaxed at the potential of zero charge (PZC), that all electron transfers must be charge neutral (e.g., proton-coupled electron transfer, PCET, rather than sequential proton/electron transfer), and that it neglects the potential dependence on energetics of chemical steps.^27−30^ GC-DFT accurately describes electrochemical mechanisms due to its self-consistent treatment of the electrified interface under an applied potential and its ability to capture decoupled charge transfer, widely believed to be a relevant elementary step in CO_2_R (CO_2_ + * + e^–^ → CO_2_^–^*, where * represents a surface active site).^4,30,31^ We also use attenuated total reflection surface-enhanced infrared absorption spectroscopy (ATR-SEIRAS) to investigate metallic and oxidized Sn surfaces during electrocatalysis. We recently developed a method of preparing Sn-based films that exhibit excellent surface enhancement in ATR-SEIRAS.^32^ Much of the ongoing debate in the community regarding CO_2_R on Sn originates from the lack of reliability in preparing and characterizing model Sn surfaces for spectroscopic investigation, as well as ambiguous frequency assignment.^10,21^ By carefully comparing our observations from the GC-DFT and ATR-SEIRAS studies, we show that CO_2_R is feasible on both metallic and oxidized Sn through a combination of pathways that lead to adsorbed formate. Based on these results, metallic Sn is expected to show stronger competition from the hydrogen evolution reaction (HER) because of its lower affinity for CO_2_R intermediates, whereas oxidized Sn is hindered by overbound formate and competition with molecularly adsorbed water and electrolyte ions.

## Methods

### Materials

All solutions were prepared in 18.2 MΩ deionized water (Elga PURELAB flex 1). CO_2_ (4.0, Airgas), Ar (5.0, Airgas), 96% sulfuric acid (Suprapur, Merck), 65% nitric acid (Suprapur, Merck), tin(II) sulfate (≥95%, Sigma-Aldrich), and potassium bicarbonate (Certified ACS Crystalline, Fisher Chemical) were used as received.

### ATR-SEIRAS

In situ ATR-SEIRAS experiments were performed on a Nicolet 6700 FTIR spectrometer (Thermo Fisher Scientific) with a VeeMAX III ATR chamber (PIKE Technologies). The spectro-electrochemical experiments were performed in a J1W Jackfish spectro-electrochemical cell (PIKE Technologies) with a PTFE/PEEK base. The synthesis and characterization of ATR-SEIRAS-active Sn-based films have been described in detail elsewhere.^32^ Other reports have also detailed the synthesis of electrodeposited metal films for SEIRAS, but not films of metal oxides.^33,34^ Briefly, a polycrystalline Au underlayer was chemically deposited onto a Si(100) specialized 1 ATR element (single-bounce ATR crystal, IRUBIS) according to the procedure reported by Osawa.^35^ This underlayer was electrochemically cycled between 0.2 and 1.75 V_RHE_ at 50 mV/s for 20 cycles with a Au counter electrode to achieve a clean, SEIRAS-active film. Metallic Sn was electrodeposited onto the Au underlayer at −0.467 V_Ag/AgCl_ in 0.1 M H_2_SO_4_ until the total charge passed was 37.9 mC/cm_geo_^2^, which corresponds to a ∼10 nm thick Sn film. Oxidized Sn was deposited onto the Au underlayer by precipitating SnO_2_ directly at the electrode surface by controlling the local pH via nitrate reduction to nitrite. The nitrate reduction was performed at −0.6 V_Ag/AgCl_ in 1.5 M HNO_3_ until the total charge passed was 3.16 C/cm_geo_^2^, which corresponds to a ∼20 nm thick SnO_2_ film. The oxidized Sn film was reductively pretreated at −0.4 V_RHE_ for 5 min in 0.1 M KHCO_3_ to improve the film’s conductivity. Both Sn film deposition procedures used a graphite counter electrode. A non-Pt counter electrode was used during the Au underlayer and Sn film preparation was to avoid Pt dissolution and electrodeposition on the working electrode surface, which can cause erroneous SEIRAS features.^36,37^ Once the Sn film was synthesized, spectroscopy was acquired using a Pt counter electrode which only experienced a linear anodic current sweep. Pt dissolution has been shown to be initiated during the oxide reduction process when switching from high anodic potentials back toward cathodic potentials.^38,39^ Therefore, the use of a Pt counter electrode should not convolute the SEIRAS features on Sn electrodes. CO_2_R experiments were performed on metallic or oxidized Sn electrodes by sparging the electrolyte (0.1 M KHCO_3_) with CO_2_ for 20 min before starting the experiment. Cyclic voltammograms were collected using a Gamry Interface 1010 potentiostat at a sweep rate of 1 mV/s from −0.25 to −1 V_RHE_. The uncompensated resistance was corrected using the current interrupt compensation feature of the potentiostat. Control experiments were also performed, where the electrolyte was sparged/blanketed with Ar rather than CO_2_. All electrochemical experiments were performed using a homemade Ag/AgCl reference electrode, calibrated, and subsequently converted to the RHE potential scale.

### Computational Details

**Figure 1:**
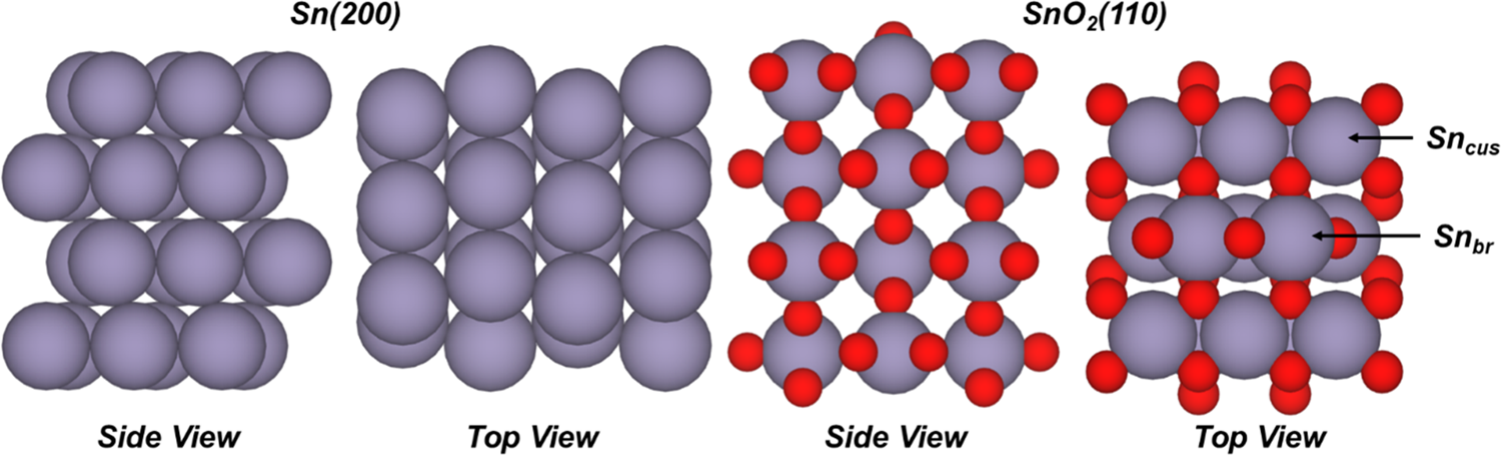
Side and top views of Sn(200) and SnO_2_(110) surfaces.

GC-DFT calculations were performed using the open-source JDFTx software.^40^ The generalized-gradient approximation PBE DFT functional with Grimme’s D3 dispersion corrections was used for all calculations.^41,42^ The Brillouin zone was sampled using a γ-centered 4 × 4 × 1 folded *k*-point mesh. The core electrons were modeled with GBRV v1.5 ultrasoft pseudopotentials with an energy cutoff of 20 hartree (544 eV) and a charge density cutoff of 100 hartree (2721 eV).^43^ Charge neutrality was ensured by the inclusion of the CANDLE implicit solvation model.^44^ The fluid solvent was water with 0.5 M NaF and was chosen as a noninteracting electrolyte. The constant potential calculations were performed by setting the electron chemical potential, μ_calc_, to the desired potential via eq 1
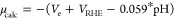
1where *V*_e_ is the absolute electron potential (taken to be 4.66 eV, calibrated using the CANDLE solvation model^44^), *V*_RHE_ is the desired potential on the RHE scale, and pH is the solution pH being modeled. Our calculations were performed at 0, −0.5, and −1 V_RHE_ and a pH of 8 to match the experimental conditions (energies were linearly interpolated between potentials to find equilibrium potentials reported in Results and Discussion). Bulk body-centered tetragonal (bct) Sn and rutile SnO_2_ structures, acquired from the Materials Project,^45^ were first relaxed and reproduced the experimental lattice parameters within 2% accuracy (calculated *a* = 5.958 Å, *c* = 3.157 Å and experimental *a* = 5.838 Å, *c* = 3.180 Å for bct Sn;^46^ calculated *a* = 4.811 Å, *c* = 3.232 Å and experimental *a* = 4.741 Å, *c* = 3.187 Å for rutile SnO_2_^47,48^). The lowest energy surfaces of each material were taken to be the 200 and 110 surface termination for bct Sn and SnO_2_, respectively.^49,50^ These surfaces were created using the Pymatgen python package.^51^ Both surface unit cells, shown in Figure 1, contained 48 atoms (16 Sn and 32 O atoms for SnO_2_(110)), and were 4 atomic layers thick, with at least 30 Å separating the slabs to ensure adequate potential screening. The bottom two atomic layers were frozen to their bulk coordinates. Geometry optimizations were performed using the Atomic Simulation Environment (ASE) python package.^52^ Geometry optimizations were considered converged when the net force on the atoms was lower than 0.05 eV/Å. Vibrational frequencies for relevant adsorbates were calculated within JDFTx, with all atoms frozen aside from the adsorbate atoms and surface atoms directly bound to the adsorbate. Grand free energy was calculated according to eq 2
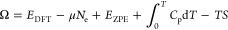
2where *E*_DFT_ is the electronic energy, μ is the potential of the calculation, *N*_e_ is the number of electrons in the calculation, *E*_ZPE_ is the zero-point energy, *C*_p_ is the heat capacity, *T* is the temperature, and *S* is the total entropy (sum of translational, vibrational, rotational and electronic). For molecules, the free energy corrections were determined from the ideal gas partition function using ASE’s thermochemistry IdealGasThermo package. To investigate the vibrational frequency dependence on potential (Stark shift), we also computed the vibrational frequencies (at 298 K) as a function of potential. All converged geometries are shown in Figure S1.

## Results and Discussion

### Sn Surface Speciation Under Relevant Electrochemical Conditions

**Figure 2:**
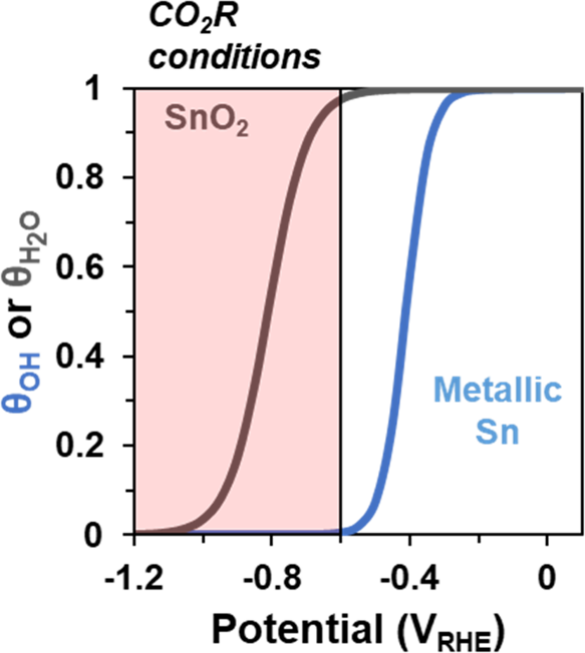
Coverage of OH* on Sn(200) and cus-water on SnO_2_(110) as a function of potential, as derived from the potential-dependent change in grand free energy and Langmuir isotherm (eq 3, temperature = 298.15 K, pH = 8 and *a*_H_2_O_ = 1).

Before examining the thermodynamics of CO_2_R intermediates on Sn surfaces, we first evaluated the potential-dependent interactions of both metallic and oxidized Sn with water in the absence of CO_2_ across the range of potentials used in experiments. We performed this analysis because it is commonly claimed that surface hydroxylation is a key element for effective CO_2_R catalysis on Sn-based electrodes.^10,13,21^ For metallic Sn, we examined the oxidative adsorption of hydroxyl (Sn + OH^–^ → Sn – OH + e^–^) as well as associative adsorption of water molecules. We found that water spontaneously desorbed at all potentials on the Sn(200) surface. Figure 2 shows the coverage of hydroxyls on Sn(200) using a potential-dependent Langmuir adsorption isotherm, shown in eq 3
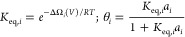
3

The coverage of hydroxyls on Sn(200) approaches zero for potentials more negative than −0.6 V_RHE_. Therefore, for potentials more negative than −0.6 V_RHE_ on fully metallic Sn surfaces, we do not expect surface hydroxyls to make a significant contribution to CO_2_R.

Oxidized Sn has a more complex surface speciation, summarized in Figure S2. Stoichiometric metal oxides are known to spontaneously dissociate water to form surface hydroxyls.^53−55^ These surface hydroxyls are also known to have different Brønsted acid–base properties, namely that the hydroxyls bound to 5-fold coordinate metal sites (coordinatively unsaturated, or cus-) are more basic than those bound to 6-fold coordinate metal sites (bridge-bound, or br-).^56^ We used the stoichiometric SnO_2_(110) surface as our model starting point. The results of our calculations agree that stoichiometric SnO_2_(110) spontaneously dissociates water to form cus- and br-hydroxyls at all potentials studied (more positive than −1 V_RHE_). We also calculated the energy of protonating the cus-hydroxyls and found that, at potentials more negative than −0.5 V_RHE_, the formation of cus-water groups is also spontaneous. Lastly, we considered the molecular desorption of water from cus- sites and found that it became favorable at potentials more negative than −0.8 V_RHE_. We found that br-hydroxyls could not be protonated; if a neighboring cus-hydroxyl site was available, the proton was spontaneously transferred to form the more stable cus-water, and if no cus-hydroxyl sites were available, the change in grand free energy was greater than 2 eV at all potentials.

CO_2_R on Sn-based electrodes is generally carried out at potentials more negative than −0.5 V_RHE_, so we considered the starting oxidized surface to be fully hydroxylated/protonated (where all br- sites are br-hydroxyl and all cus- sites are cus-water). Figure 2 also shows the coverage of cus-water as a function of potential. The coverage remains much higher than fully metallic Sn, only approaching zero at potentials more negative than −1.1 V_RHE_. Therefore, in the potential regime relevant to CO_2_R (−0.5 to −1 V_RHE_), a non-negligible coverage of cus-water and br-hydroxyl is present. Consequently, we considered the clean Sn(200) (that is, no surface hydroxyls) and a SnO_2_(110) surface with partial cus-water coverage (one cus-water and one cus-Sn with two br-hydroxyls), as well as with no cus-water groups (two cus-Sn and two br-hydroxyls). The other consequence of this finding is that a CO_2_R mechanism that invokes the formation of surface bicarbonate/carbonate via nucleophilic attack of hydroxyls to CO_2_ is unlikely to substantially contribute to the CO_2_R activity of Sn-based electrodes.

### Activation of CO_2_

**Figure 3:**
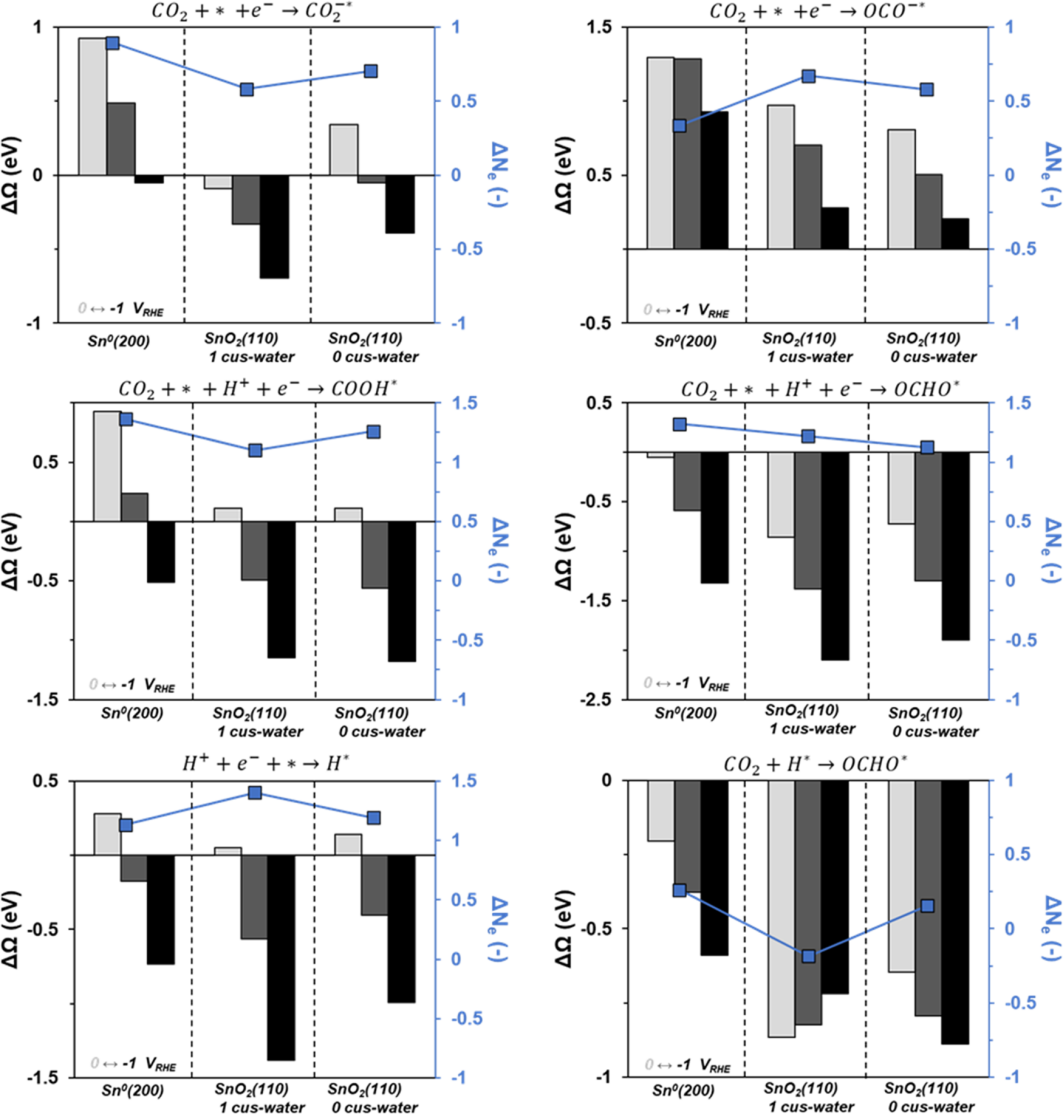
Change in grand free energy as a function of potential for different CO_2_ adsorption elementary steps. The potentials are 0 (light gray), −0.5 (dark gray), and −1 (black) V_RHE_. The reactions represented are reductive adsorption of CO_2_ through the carbon atom (top left) and oxygen atom(s) (top right), proton-coupled electron transfer adsorption to form COOH* (middle left) and bidentate OCHO* (middle right), and proton adsorption (bottom left) and CO_2_ insertion into a metal-hydride bond to form monodentate OCHO* (bottom right). The average number of electrons transferred across the three potentials is also shown (in blue).

It has been proposed that the origin of selectivity toward formate over CO in CO_2_R relates to bifurcation in pathways during CO_2_ activation. One pathway creates OCHO* adsorbed via surface-oxygen(s) bonds, while the other creates COOH* adsorbed via a surface-carbon bond.^24^ However, it is also often proposed that the first elementary step of CO_2_R (to either product) is the single-electron reductive adsorption CO_2_ + * + e^–^ → CO_2_^–^.^10,13,16,57^ The CO_2_^–*^ intermediate can be bound through the carbon atom or the oxygen atom(s), which can be protonated to form COOH* or OCHO*, respectively. We therefore considered both the single-electron reductive adsorption and each of the PCET pathways to activate CO_2_. We hypothesize that selectivity toward formate originates with the preference of Sn to activate CO_2_ through the oxygen atoms rather than the carbon atom. We calculated the adsorption energy of CO_2_ in both carbon-bound and oxygen-bound geometries as well as COOH* and OCHO* on the clean metallic Sn(200), SnO_2_(110) with 1 cus-water, and SnO_2_(110) with no cus-water surfaces as a function of potential, shown in Figure 3. It is immediately apparent however that the selective formation of OCO^–*^ vs CO_2_^–*^ is not a reasonable explanation for the selectivity to formate vs CO, on metallic or oxidized Sn, regardless of potential. Reductive adsorption of CO_2_ to form OCO^–*^ is not favorable at any potential more positive than −1 V_RHE_—in fact, CO_2_ spontaneously desorbs during geometry optimization at 0 and sometimes −0.5 V_RHE_, see the Supporting Information, Figures S3 and S4 for a more detailed discussion of this point. In contrast, the formation of CO_2_^–*^ becomes favorable at −0.99 and −0.45 V_RHE_ on metallic Sn and SnO_2_ with no cus-water, respectively, while the formation of CO_2_^–*^ is favorable at all examined potentials on the SnO_2_ surface with one cus-water. Therefore, OCO^–*^ is not expected to play a substantial role in CO_2_R toward formate. We note that in either case, the number of electrons transferred determined from the GC-DFT is not exactly one, but rather in the range of 0.4–0.9, depending on the surface and binding geometry. This demonstrates the utility of GC-DFT’s ability to determine, rather than assume, the extent of charge transfer.

The formation of OCHO* is more favorable than COOH* across all potentials and surfaces, which is consistent with the experimentally observed selectivity trend. We did not calculate any transition state energies in the current study, and while a large kinetic barrier to forming OCHO* (or COOH*) via the PCET mechanism may exist, we expect the more exothermic step to also possess a lower activation barrier on the basis of Brønsted–Evans–Polanyi (BEP) scaling relationships.^58−60^ Once again, we note that the number of electrons transferred in this step is not precisely what would be presumed from writing out the elementary steps, but in this case, more electrons (1.1–1.4) are transferred than expected. The consequence of this asymmetry in the degree of charge transfer is that the PCET adsorption of CO_2_ to form either COOH* or OCHO* becomes more favorable at less reducing potentials than the reductive adsorption to form CO_2_^–*^.

We also considered the formation of monodentate OCHO* via CO_2_ insertion into the Sn–H bond, known as the Eley–Rideal pathway, which has been suggested as a possible pathway for formate-selective CO_2_R.^61^Figure 3 (bottom left) shows the energetics for proton reduction for the three different surfaces as a function of potential. The formation of H* becomes favorable at −0.29, 0.26, and −0.12 V_RHE_ on the metallic Sn(200), SnO_2_(110) with one cus-water, and SnO_2_(110) with zero cus-water surfaces, respectively. Therefore, under potentials relevant for CO_2_R, we expect a non-negligible coverage of H*, especially considering the adsorption energy is more favorable than for the adsorption of CO_2_^–*^. Figure 3 (bottom right) shows the energetics for insertion of CO_2_ into the Sn–H bond. For all surfaces at all potentials, the formation of OCHO* in this fashion is favorable. There is less than a 0.1 eV difference in the formation energy for monodentate OCHO* and bidentate OCHO*. The potential dependence of the energetics of CO_2_ insertion into the Sn–H bond is attenuated when compared to the Faradaic reactions in the other panels of Figure 3 because there is a lower, near-zero amount of electron transfer involved in this reaction, i.e., this is a chemical step. The origin of the potential dependence of this step may be due to second-order effects such as the interaction of the adsorbate dipole with the developing electric field, or changes in the stabilization of the electrolyte due to changes in the local concentration of electrolyte ions.^62^

### Formation of Desorbed CO and Formate

The previous section compared the different pathways CO_2_ may take to form an activated adsorbate on the surface of Sn-based catalysts. We now consider the next steps of CO_2_R that involve the desorption of the products CO and formate. We started by calculating the adsorbate geometry of both CO and formic acid on the three Sn surfaces but found that they do not adsorb under any conditions considered (Figures S5 and S6). Therefore, moving forward we assume that any step that forms either CO or formic acid includes spontaneous desorption.

**Figure 4:**
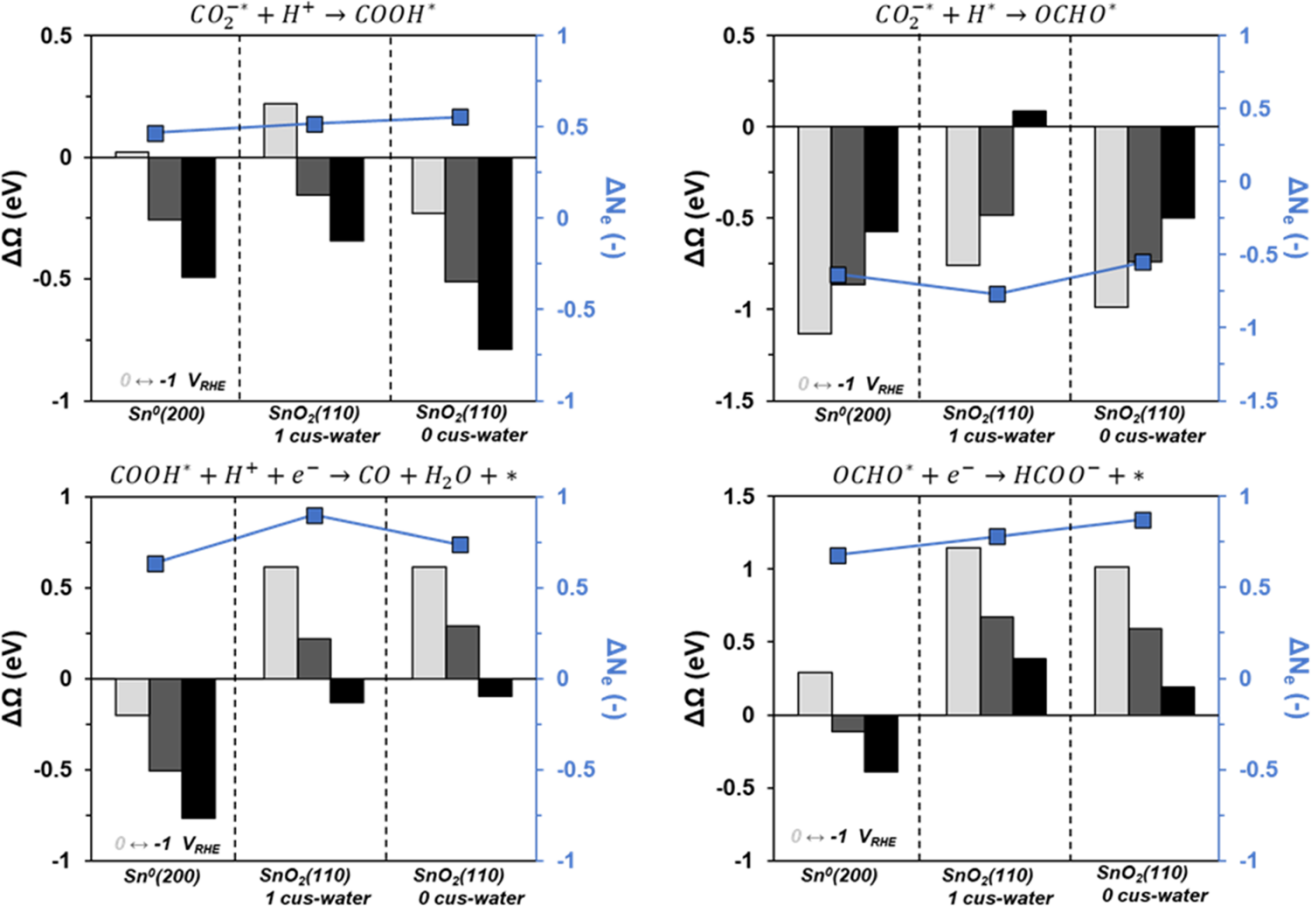
Change in grand free energy as a function of potential for the protonation of CO_2_^–*^ (top left), coupling of CO_2_^–*^ and H* (top right), reduction of COOH* to form CO (bottom left), and reductive molecular desorption of OCHO* (bottom right). The potentials are 0 (light gray), −0.5 (dark gray), and −1 (black) V_RHE_. The average number of electrons transferred across the three potentials is also shown (in blue).

First, we considered reactions involving CO_2_^–*^. We note that the energetics presented in the previous section do not rule out the formation of CO_2_^–*^ simply because the PCET adsorption to form COOH* or OCHO* is more downhill. The presence of a large kinetic barrier for the PCET steps and sufficiently low barrier for the direct reductive adsorption of CO_2_^–*^ could lead to non-negligible flux through this pathway, particularly on the oxide surfaces which bind CO_2_^–*^ more strongly than metallic Sn. Both COOH* and OCHO* are accessible from CO_2_^–*^, the former likely coming from a proton transfer and the latter likely coming from a surface-catalyzed coupling of CO_2_^–*^ with H*. The energetics for these reactions are depicted in Figure 4. Protonation of CO_2_^–*^ is not purely a proton transfer—the GC-DFT calculation predicts that ∼0.5 electrons are transferred during this step as well. The formation of COOH* in this manner is mostly favorable across the three surfaces at all potentials considered. The Langmuir–Hinshelwood coupling between H* and CO_2_^–*^ to form OCHO* is also mostly favorable across all potentials and surfaces studied, although interestingly the calculated electron transfer shows that this is an oxidation, with ∼0.5 electrons transferred to, rather than from, the electrode. This step therefore becomes less favorable with more negative potential.

Next, we consider the reactions that form the CO or desorbed formate products (Figure 4). We examined a PCET reduction of COOH* to form CO and water as the pathway to CO and the reductive molecular desorption of OCHO* as the primary pathway to formate. We also considered the Langmuir–Hinshelwood coupling of COOH* and H* to form formic acid directly, but due to a less favorable change in grand free energy that becomes increasingly less favorable at more negative potential than the direct desorption, we do not consider it to be an active pathway (Figure S7). Interestingly, metallic Sn has a much larger driving force to form CO than either of the oxide surfaces, which only become favorable at potentials more negative than −0.8 V_RHE_. This is somewhat expected based on the observation that COOH* is bound less strongly on the metallic Sn surface than on the SnO_2_ surfaces. The same trend is observed when considering the reductive molecular desorption of OCHO*, where the desorption is more favorable on the metallic surface than the oxide. Without explicit calculation of transition state energetics, we cannot definitively say which of these steps will be kinetically faster, but molecular adsorption/desorption steps typically do not have significant activation barriers. Complex reaction coordinates such as reducing COOH* to CO and water may have an appreciable activation barrier, so the reductive desorption of OCHO* and formation of CO may be relatively competitive in rate.

### Competition by HER and Carbonate Adsorption

The competition between cathodic reactions and HER is ubiquitous in electrocatalysis. An additional parasitic reaction that can lower the efficiency of CO_2_R is the strong adsorption of bicarbonate and carbonate, which are present in CO_2_R electrochemical cells either as supporting electrolyte or formed spontaneously from the equilibrium of CO_2_ and water/hydroxide. To holistically evaluate the performance of metallic and oxidized Sn catalysts for CO_2_R, we also considered the competition from HER and bicarbonate/carbonate adsorption.

HER may follow two well-known mechanisms: the Volmer–Heyrovsky and Volmer–Tafel mechanisms.^63^ HER on Sn is thought to mainly follow the Volmer–Heyrovsky mechanism due to low coverage of H* until larger overpotentials, by which time the rate of the Heyrovsky step becomes fast enough to scavenge H*.^64^ We consider both mechanisms for the sake of completeness. Figure S8 shows the reaction coordinate diagrams for both the Volmer–Heyrovsky and Volmer–Tafel mechanisms on all three surfaces as a function of applied potential. The results agree with the existing literature that the Volmer–Heyrovsky mechanism is preferred on all Sn surfaces, with all steps being thermodynamically downhill at potentials more negative than −0.3 V_RHE_. The presence of a large kinetic barrier for the Heyrovsky step could lead to non-negligible flux through the Volmer–Tafel mechanism on metallic Sn once an appreciable coverage of H* has accumulated. Each Volmer step transfers 1.1–1.4 electrons from the electrode, meaning that 0.2–0.8 electrons must be transferred back to the electrode during the Tafel step. Therefore, the Tafel step becomes less favorable at more negative potentials. This is more pronounced on the SnO_2_ surfaces, so it is even more likely that the reaction proceeds through the Volmer–Heyrovsky mechanism on these surfaces. The most significant consequence of this is that, because the Volmer step is potential-determining on all three surfaces (for the Volmer–Heyrovsky mechanism), the surface coverage of H* is likely to be low (but not zero) because of the favorable reaction between either H^+^ to from H_2_ or CO_2_ to form OCHO*. This will negatively impact the rate of CO_2_R steps that involve H* unless there is a much lower kinetic barrier for these steps than the Heyrovsky step.

**Figure 5:**
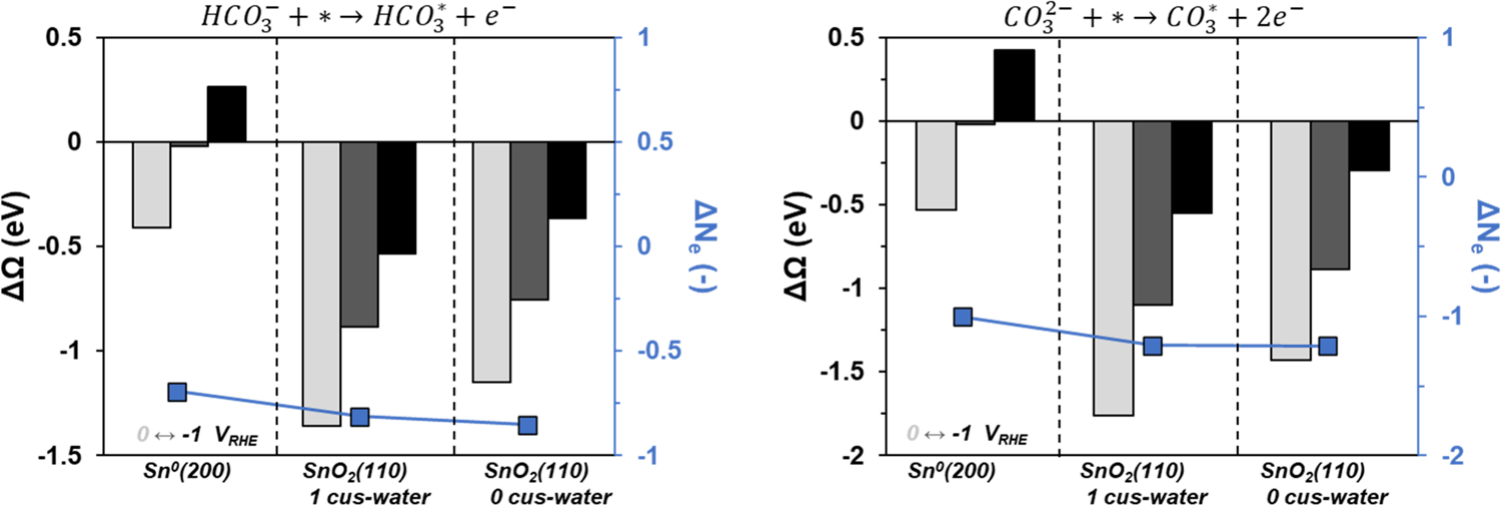
Change in grand free energy as a function of potential for the molecular adsorption of bicarbonate (left) and carbonate (right). The potentials are 0 (light gray), −0.5 (dark gray), and −1 (black) V_RHE_. The average number of electrons transferred across the three potentials is shown (in blue).

Next, we considered the competitive adsorption of bicarbonate/carbonate. Bicarbonate is often the supporting electrolyte of choice for CO_2_R because it can retard the aqueous CO_2_ equilibrium, which results in efficiency losses.^65,66^ This aqueous CO_2_ equilibrium means that bicarbonate will likely be present even in electrolytes that do not have intentionally added bicarbonate and therefore must be considered when evaluating the surface processes of CO_2_R catalysts. Lastly, carbonate may begin to accumulate near the electrode interface due to the increase in pH that arises from the consumption of protons during cathodic reactions.^67^ It is not unusual for the interfacial pH of a cathode to be 2–4 pH units higher than the bulk pH, meaning the interfacial pH could be greater than the p*K*_a_ of bicarbonate.^68^Figure 5 shows the energetics for the molecular adsorption of bicarbonate and carbonate on the three Sn surfaces as a function of potential. In line with the results described above for all previously discussed adsorbates, metallic Sn binds both bicarbonate and carbonate more weakly than either of the SnO_2_ surfaces. The adsorption of bicarbonate and carbonate becomes less favorable at more negative potentials as expected due to the net oxidation during adsorption. The net electrons transferred for bicarbonate is close to the expected value of 1, but the number of electrons transferred for carbonate is much lower than the expected value of 2, only slightly above 1. This means the carbonate adsorbate is partially charged and the adsorption energy is less sensitive to potential than expected. We also considered the energetics of the adsorption of bicarbonate with a simultaneous discharge of its proton to form adsorbed carbonate (Figure S9), but this process was less favorable than either of the two molecular adsorption processes and is therefore not expected to contribute significantly to the adsorption processes. This evidence suggests that molecular adsorption of bicarbonate and carbonate can compete with other intermediates for active sites at low potential, but will be driven off of the surface at more negative potentials.

Before discussing the results of the ATR-SEIRAS experiments, we summarize the observations from the preceding sections. First, metallic Sn is unlikely to have an appreciable coverage of hydroxyls during CO_2_R, whereas oxidized Sn may have a non-negligible coverage of hydroxyls and water groups throughout the CO_2_R relevant potential window. Next, CO_2_ has four feasible activation pathways: direct reductive adsorption to form CO_2_^–*^ bound through the carbon atoms, PCET adsorption to form either COOH* or OCHO*, and insertion of CO_2_ into a Sn–H bond to form OCHO*. CO_2_^–*^ can subsequently be protonated to form COOH*, which may react to form CO and H_2_O in a final PCET, or react with H* to form OCHO*, which subsequently undergoes reductive molecular desorption to form solution-phase formate. A non-negligible competition may exist between CO_2_R and (i) molecular adsorption of bicarbonate/carbonate at low overpotential and (ii) Volmer–Heyrovsky HER at higher overpotential.

### ATR-SEIRAS on Metallic and Oxide-Derived Sn

**Figure 6:**
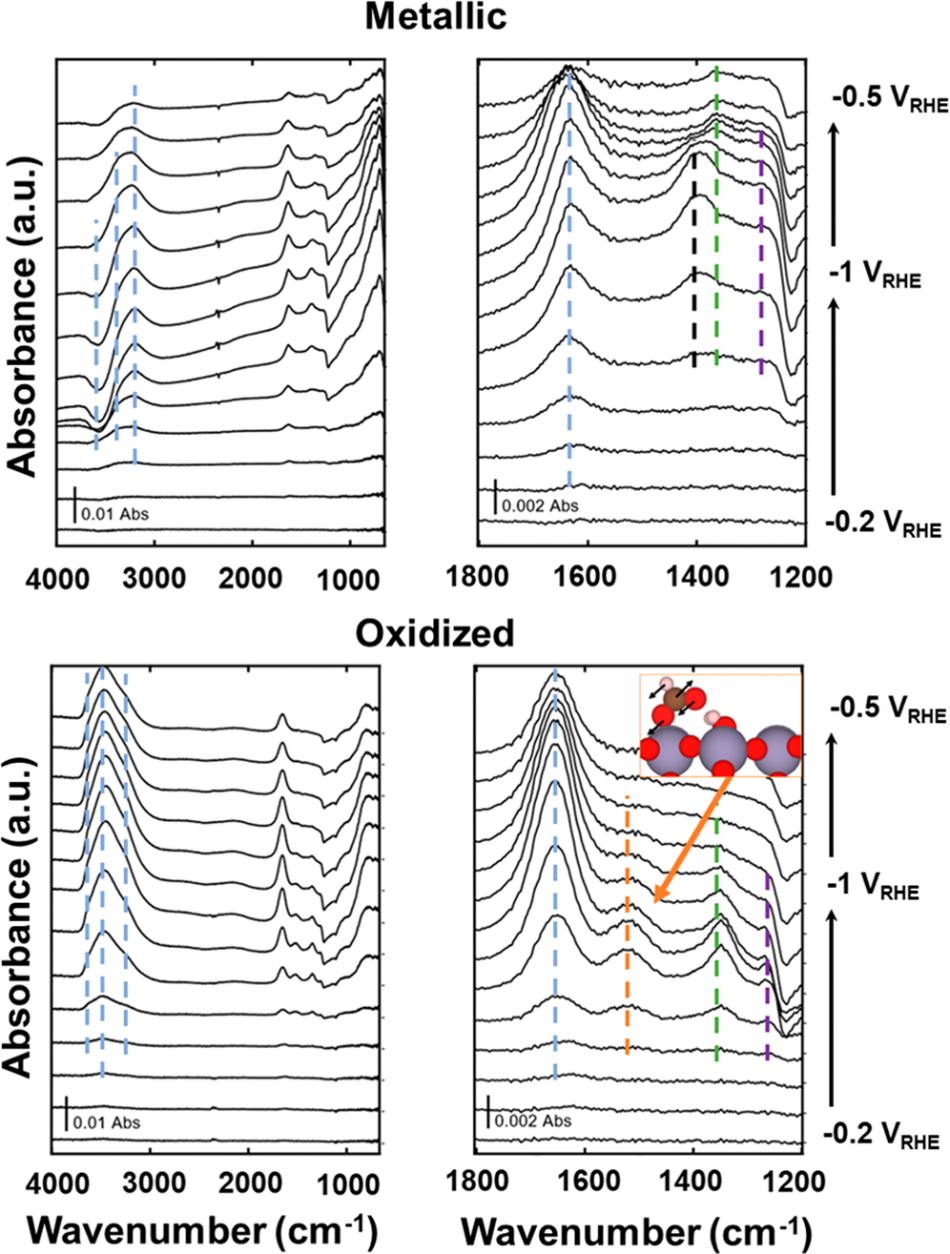
Full (4000–800 cm^–1^, top left) and carbonaceous region (1800–1200 cm^–1^, top right) ATR-SEIRAS spectra on the metallic Sn electrode during CO_2_R. Full (4000–800 cm^–1^, bottom left) and carbonaceous region (1800–1200 cm^–1^, bottom right) ATR-SEIRAS spectra on the oxidized Sn electrode during CO_2_R. Colored lines are drawn to guide the eye (spectra without these lines are available in the Supporting Information, Figures S34 and S35). Conditions: 0.1 M KHCO_3_ (pH = 8) with bubbling CO_2_, Sn, or SnO_*x*_ thin-film deposited on Au working electrode, Pt wire counter electrode and Ag/AgCl reference electrode. Inset: monodentate formate adsorbed on SnO_2_(110) with no cus-waters with annotated vibration vectors for the calculated vibrational frequency of 1519 cm^–1^.

With some degree of understanding of what might be present on the surface during CO_2_R from the GC-DFT analysis described above, we performed ATR-SEIRAS experiments to validate and refine this understanding. We previously reported a method to prepare both fully metallic and oxidized SEIRAS-active Sn films, which we used in the present study to discern differences in adsorbed species during CO_2_R.^32^ Before we begin the discussion of the SEIRAS results, we would like to point out that ex situ characterization of the two Sn films after the reductive treatment shows that they have similar degrees of oxidation. Both Sn films undergo spontaneous oxidation in air, which complicates the assignment of the exact oxidation state of the two films during CO_2_R. Dutta et al. performed operando EXAFS experiments on SnO_2_-based catalysts and found that metallic Sn coordination was not observed until −0.88 V_RHE_, and SnO_2_ and SnO features were still observed even at −1 V_RHE_.^18^ Therefore, while the oxidized Sn film undoubtedly partially reduces under the electrochemical conditions present in CO_2_R, we do not necessarily expect it to resemble the fully metallic Sn film under the conditions examined and spectroscopic differences between the two Sn films may still appear. Figure 6 shows the ATR-SEIRAS spectra for both the metallic and oxidized Sn film as a function of potential during CO_2_R. We point out two common features for both samples, at ∼1200 and ∼1100 cm^–1^. These features are attributed to the Si(100) phonon that comes from the wafer used to perform the ATR-SEIRAS experiment.^69^ There may also be trace amounts of sulfate that remain from the electrosynthesis of the Sn films. Both features are present during blank experiments with no CO_2_ or bicarbonate present and will not be discussed or interpreted further. The observed vibrational frequencies are collected in Table 1.

**Table 1:** Observed Vibrational Frequencies (in cm^–1^) for the Metallic and Oxidized Sn Films from Figure 6, along with the Corresponding Assignments

metallic Sn		oxidized Sn	
frequency (cm^–1^)	assignment	frequency (cm^–1^)	assignment
3562 (loss)	desorption of OH*	3644	interfacial water
3366	interfacial water	3486	interfacial water
3209	interfacial water	3256	interfacial water
∼2400 (loss)	consumption of CO_2_	∼2400 (loss)	consumption of CO_2_
1611	interfacial water	1635	interfacial water
1410	solution-phase carbonate	1524	monodentate formate
1363	monodentate formate	1349	monodentate formate
1282	monodentate formate	1267	monodentate formate

First, we observe positive bands in the 3200–3500 cm^–1^ region that could be assigned to changes in the interfacial water structure during electrode polarization.^70,71^ The band at 1611 and 1635 cm^–1^ for metallic and oxidized Sn, respectively, is likely also related to changes in interfacial water structure. A difference between the two materials is seen in the high-frequency O–H stretch region, where metallic Sn has a negative band at 3562 cm^–1^ and oxidized Sn has a positive band at 3644 cm^–1^. Given that the calculated PZC for Sn(200) is −0.52 V_RHE_ and the onset of the negative band is at −0.6 V_RHE_, the O–H stretches might be attributed to shifts in the interfacial water structure. However, these high-frequency spectral features have also previously been assigned to “dangling” surface hydroxyls that have less hydrogen bonding than water.^56^ Given that GC-DFT predicts that hydroxyls will desorb from metallic Sn at a similar potential, the negative IR peak is also consistent with the desorption of surface hydroxyls. This also explains why no such negative peak appears for the oxidized Sn surface, where the cus-hydroxyls spontaneously form cus-water, and br-hydroxyls are not predicted to desorb at these potentials. Furthermore, the negative IR peak is fully reversible, which implies that as the potential is returned to the starting value, the vibrating species can return to their prior state.

Focusing now on the bands that may be associated with carbon-bearing species, both the metallic and oxidized Sn materials have loss bands at ∼2400 cm^–1^ that appear between −0.5 and −0.6 V_RHE_ (although it is more apparent on the metallic Sn surface). These bands can be assigned to the consumption of CO_2_ during electrolysis. However, it is not immediately obvious if this is simply due to the reactions of CO_2_R, or if some of the CO_2_ is converted to aqueous bicarbonate and carbonate at the electrode interface due to the cathodic pH increase. We refrain from interpreting the magnitude of these bands because of this ambiguity. Next, there is a band at 1524 cm^–1^ that is only present on the oxidized Sn surface and grows between −0.5 and −0.6 V_RHE_, before mostly decreasing to the background level. A peak in this region has previously been interpreted as formate,^72^ bicarbonate^21^ and carbonate,^10^ so we attempted to assign this band by calculating the vibrational frequencies. Table S1 shows the GC-DFT-calculated vibrational frequencies as a function of potential for the Sn(200), SnO_2_(110) with 1 cus-water, and SnO_2_(110) with no cus-waters. For the oxidized Sn surfaces, the observed vibrational frequency is most consistent with monodentate formate and bidentate bicarbonate adsorbates. However, due to the surface selection rule, only adsorbate vibrational modes with a change in dipole that is perpendicular to the surface will be IR active. Visualizations of the vibrational modes are provided in Figures S10–S33 (gif file animations are also available in the Supporting Information). For both monodentate formate and bidentate bicarbonate, the vibrational mode responsible for the frequency that is closest to the observed value is the OCO asymmetric stretch. The vibration coordinate, shown as an inset in Figure 6, is more perpendicular for monodentate formate than for bidentate bicarbonate, so we assign this peak as monodentate formate. An additional clue that this is less likely to be bicarbonate and more likely to be formate is that we predicted the formation of formate to become increasingly favorable and the adsorption of bicarbonate to become decreasingly favorable at more negative potentials. Monodentate formate was assigned to a feature at 1680 cm^–1^ by Jiang et al. when studying CO_2_R on Pd-based electrocatalysts.^72^ They also assigned a peak at 1584 cm^–1^ to solution-phase, desorbed formate. This peak position differs significantly from both what we have observed in our own spectra and calculated using GC-DFT, but the difference could be explained by both differences in vibrational frequencies across different materials and under different electrochemical environments.

The next peak observed and used for mechanistic analysis is only present in the metallic Sn catalyst spectra as a reversible peak that appears at 1410 cm^–1^ at potentials more negative than −0.6 V_RHE_. This feature is most consistent with either monodentate carbonate, or solution-phase carbonate which would arise from the increase in interfacial pH. GC-DFT calculations predict that carbonate will desorb from metallic Sn at these potentials, so it is more likely that solution-phase carbonate has accumulated at the electrode interface. The absence of this feature in the oxidized Sn spectra is of particular interest, as the *I*–*V* curves (Figure S36) show that oxidized Sn draws a higher current (as well as higher current density) than metallic Sn, which implies that the consumption of protons at the interface should be higher on oxidized Sn than metallic Sn. This may be explained by the presence of the cus-water and br-hydroxyl groups on the oxidized Sn surface playing a buffering role, that is, when solution-phase protons are consumed in cathodic reactions, protons on the surface may be released to neutralize the change at the interface. To evaluate the feasibility of this hypothesis, we determined the favorability of transferring a proton from the SnO_2_(110) surface to a carbonate ion (Figure S37), which was favorable at all potentials. Therefore, a complex equilibrium exists at the interface of the oxidized Sn surface that may be able to neutralize the expected pH increase upon cathodic polarization. Metallic Sn is unable to perform this interfacial buffering because its surface hydroxyls have been reduced off the surface at these potentials. Assigning the relative importance of the intrinsic electrode reactivity and the local pH on the activity and selectivity of Sn-based CO_2_R catalysis would require a sophisticated coupled transport-kinetic model like Singh et al. developed for CO_2_R on Ag catalysts.^4^ Cao et al. assigned this feature to monodentate formate in their investigation of CO_2_R to formate over two-dimensional Bi catalysts.^73^ They measured this peak in the absence of CO_2_ without quantifying or detecting any formate production, so it is more likely that this peak is also due to the formation of carbonate once the pH increases from the cathodic consumption of protons.

The remaining bands, at 1363/1282 and 1349/1267 cm^–1^ for metallic and oxidized Sn, respectively, likely belong to monodentate formate. This is consistent with the calculated vibrational frequencies from GC-DFT for both surfaces. The peaks at 1363 and 1349 cm^–1^ are assigned to the combination mode of C–H wagging and OCO bending, and the peaks at 1282 and 1267 cm^–1^ are assigned to the OCO symmetric stretch. As with the formate peak at 1524 cm^–1^, this assignment is strengthened by the reversible appearance of these features at potentials more negative than −0.6 V_RHE_. One difference between the metallic and oxidized Sn spectra is that the peak at 1363 cm^–1^ is not fully reversible on metallic Sn, and a small peak is still present at −0.5 V_RHE_ on the backsweep. We assign this as readsorbed formate, but the GC-DFT calculations predict that formate is bound more strongly to oxidized Sn than metallic, so we expected that this feature would be present in the oxidized case too. However, there is also a larger driving force to readsorb hydroxyls at more positive potentials on oxidized Sn. Therefore, because a much weaker driving force exists for hydroxyl adsorption on metallic Sn, formate may only be displaced from metallic Sn at more positive potentials.

Finally, to assist with the assignments of the features in Figure 6, we repeated the SEIRAS experiment without CO_2_ present in the electrochemical cell. Figure S38 (nonannotated versions in Figures S39 and S40) shows the SEIRAS spectra for both the metallic and oxidized Sn materials. For the metallic Sn electrode, only the signals corresponding to the changes in interfacial water and the solution-phase carbonate persist in the absence of CO_2_. The solution-phase carbonate arises due to the pH increase from HER. This supports the assignment of the other peaks at 1363 and 1282 cm^–1^ as formate (or at least intermediates from CO_2_R) and not molecular adsorption of bicarbonate/carbonate present in the electrolyte. Similarly, most of the SEIRAS features disappear for the oxidized Sn electrode when CO_2_ is removed from the electrolyte. The features that arise from changes in the interfacial water remain, and a new negative feature appears at 1450 cm^–1^. This peak is most consistent with bidentate carbonate. This shows that there may be carbonate adsorption from the electrolyte on the oxidized Sn surface that can be reduced off of the surface, which is supported by the results of calculations reported in Figure 5. The disappearance of the peaks at 1524, 1349, and 1267 cm^–1^ when CO_2_ is absent confirms that these peaks originate from CO_2_R intermediates that are most likely monodentate formate.

### Proposed Mechanism and Strategies for Improved CO_2_R

**Figure 7:**
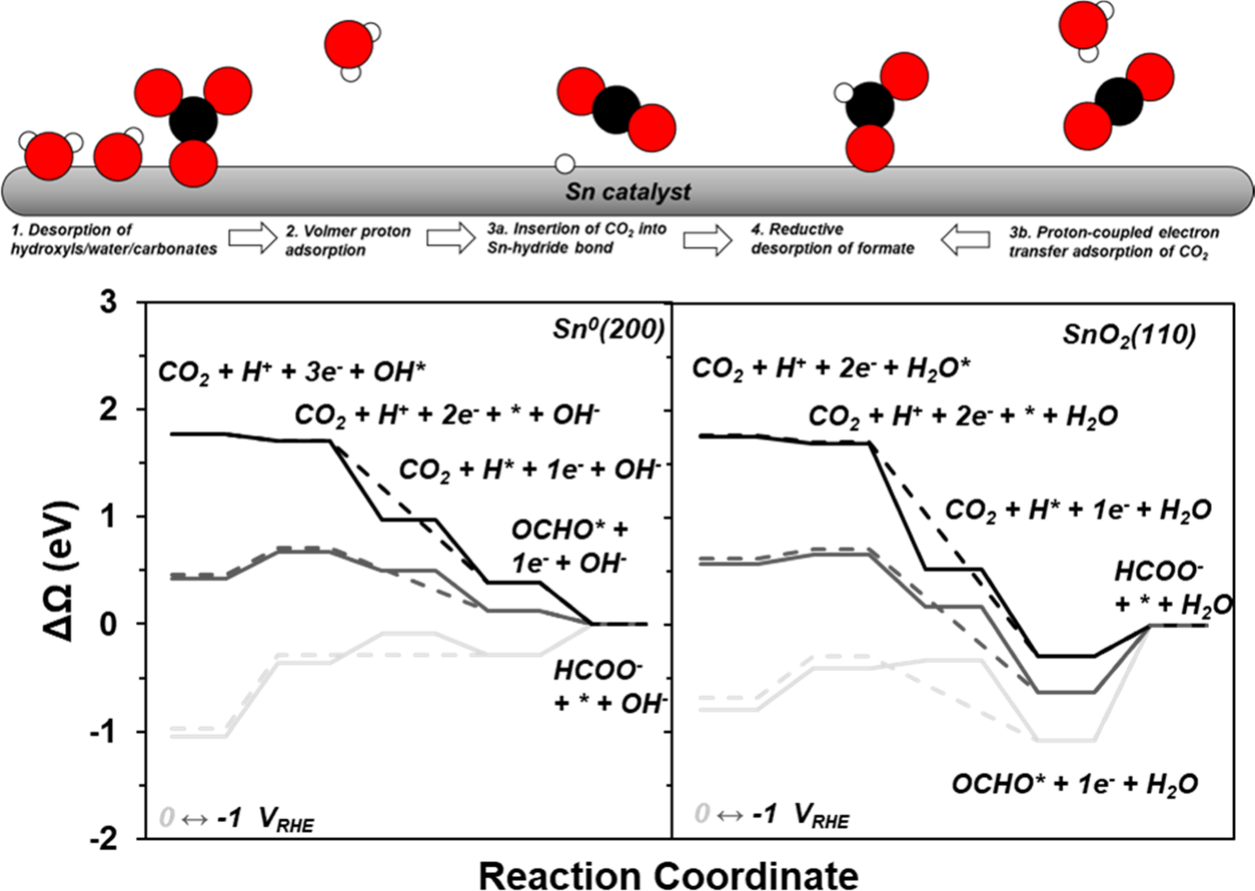
(Top) Schematic visualizing the proposed mechanism in Mechanism 1 and reaction coordinate diagram for metallic Sn (bottom left) and the average between oxidized Sn with one and no cus-waters (bottom right) for the proposed mechanism in Mechanism 1. The solid lines are for the Eley–Rideal pathway, and the dashed lines are for the PCET pathway.

Through both the GC-DFT and ATR-SEIRAS analyses described above, we have identified CO_2_R pathways that are feasible on both metallic and oxidized Sn surfaces. For metallic Sn, surface hydroxyls are reductively desorbed by −0.6 V_RHE_ (M.1). Direct reductive adsorption of CO_2_ to form OCO^–*^ does not become favorable until potentials more negative than −1 V_RHE_, which is more negative than the observed onset of peaks assigned to formate in the ATR-SEIRAS spectra. Therefore, we believe that CO_2_ is activated either by insertion into the Sn–H bond via an Eley–Rideal-like step (M.3a), preceded by Volmer proton adsorption (M.2), or PCET adsorption to OCHO* (M.3b). Only monodentate formate was detected using ATR-SEIRAS, which supports the Eley–Rideal activation of CO_2_ on metallic Sn because there is a larger driving force for formate desorption than to rearrange into the bidentate configuration. Jiang et al. made a similar conclusion regarding the formation of monodentate formate when investigating CO_2_R on Pd-based catalysts.^72^ The potential limiting step for this mechanism is the reductive molecular desorption of formate (M.4), which becomes favorable at −0.39 V_RHE_. The lack of IR bands associated with formate at this potential on metallic Sn is explained by the presence of hydroxyls, which are not predicted to completely vacate the surface until −0.6 V_RHE_. This mechanism is summarized in Mechanism 1 and Figure 7, which also shows the free energy landscape for the pathways on metallic and oxidized Sn.**Mechanism 1.** Proposed mechanism for CO_2_R to formate on Sn-based catalysts
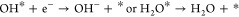
M.1
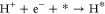
M.2
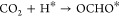
M.3a
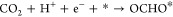
M.3b
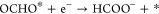
M.4

For oxidized Sn, the mechanism is more similar to metallic Sn than expected based on the differences in the energetics predicted from GC-DFT. The surface is initially blocked by cus-water groups that begin to desorb at −0.6 V_RHE_ (M.1). Next, because the oxidized Sn surface has higher affinity for all intermediates than metallic Sn, it is possible that all of the proposed CO_2_ activation pathways are accessible. However, unless there are substantial kinetic barriers for all of the steps except direct reductive adsorption of CO_2_, there is a larger driving force to form H*, COOH*, and OCHO* at potentials more negative than −0.5 V_RHE_ due to the greater extent of charge transfer. We expect that any COOH* formed will go on to produce CO, so the same two CO_2_ activation steps (Eley–Rideal reaction between CO_2_ and H* and PCET adsorption to form OCHO*) are most likely. Desorption of formate is again the potential-determining step, although for oxidized Sn it is predicted to be −1.22 V_RHE_. This implies that the active phase of Sn during CO_2_R is likely a partially reduced Sn oxide (or partially oxidized metallic Sn) such that the energetics of both activation and desorption steps are in between the two limiting cases examined in this study. We also considered the hydroxyl-mediated pathway proposed by Baruch et al.,^21^ and cannot conclusively eliminate it as a possibility but believe that is likely only a minor contribution to the overall CO_2_R rate (see the Supporting Information and Figures S41–S43). Metallic and oxidized Sn surfaces having different rate-limiting steps should manifest as different Tafel slopes. The electrochemical cell used for the SEIRAS experiments is not equipped to accurately determine the Tafel slope for CO_2_R toward formate due to influences from mass transport and very low product concentrations, so more thorough kinetic measurements to determine the reaction orders, apparent activation barriers and the Tafel slope under a broader range of conditions will be necessary to fully resolve the mechanistic differences between metallic and oxidized Sn electrocatalysts.

The proposed mechanism, and data that lead to it, highlight the factors that limit CO_2_R on Sn. On either metallic or oxidized Sn, GC-DFT predicts that the electrochemical activation of CO_2_ becomes favorable at a less negative potential than the observed onset potential of −0.6 V_RHE_, which coincides with the potential at which hydroxyls or molecular water groups are predicted to desorb from the surfaces. Therefore, lowering the overpotential of CO_2_R on Sn will require decreasing the affinity of Sn surfaces toward hydroxyls/water. Doing so will be challenging to achieve because, due to adsorbate scaling relationships, surfaces that bind one class of adsorbates more weakly typically bind all classes of adsorbates more weakly, and as such lowering the potential for hydroxyl/water removal will also increase the potential for CO_2_ activation. Some strategies for decorrelating carbon-bound and oxygen-bound adsorbates have been proposed, such as alloying with distinct elements such as sulfur and taking advantage of interfaces between different materials like RuO_2_ and CeO_2_.^15,74−77^ Controlling the near-electrode environment could also be a beneficial strategy. It has been demonstrated that the presence of electrolyte additives, such as self-assembled monolayers^70,71^ and ionomer coatings^78^ can modify the hydrophilicity of the electrode interface. Selectively destabilizing surface hydroxyls/water in this manner could lower the overpotential required to open active sites for CO_2_R on Sn surfaces.

## Conclusions

In this study, we used GC-DFT and ATR-SEIRAS to interrogate the differences in CO_2_R mechanism toward formate on both metallic and oxidized Sn surfaces. While the computed energetics and observed IR features are distinct for both materials, we have proposed probable mechanistic steps that are common to both materials, with different limiting characteristics. The steps most consistent with our study are 1. Potential-driven desorption of hydroxyls/water/bicarbonate, 2. Volmer proton adsorption, 3a. Eley–Rideal insertion of CO_2_ into a Sn–H bond, 3b. PCET adsorption of CO_2_, and 4. Reductive desorption of formate. Metallic Sn is less likely to be limited by the desorption of formate or other spectator species such as bicarbonate, carbonate, and hydroxyls, but oxidized Sn is more able to facilely activate CO_2_. In both cases, hydroxyls and molecular water must be driven off the surface to open active sites for CO_2_R. This analysis ultimately supports the conclusion that Sn surfaces are in situ oxidized or reduced to achieve an intermediate oxidation state such as Sn_2_O_3_ or SnO, that is more optimally active for the CO_2_R to formate than fully metallic or oxidized Sn. Future investigations of Sn-based electrocatalysts should focus on (1) Identifying the exact speciation of the operando Sn catalyst, (2) Calculating the explicit activation energies of the elementary steps identified here as most relevant and (3) Experimentally determining the kinetic signatures like reaction orders, apparent activation barriers, and apparent transfer coefficients across a diverse set of experimental conditions to diagnose the mechanism with more confidence.

## Supporting Information Available

GC-DFT calculated adsorption geometries; discussion of SnO_2_(110) surface speciation; additional discussion of GC-DFT for computational electrochemistry; additional discussion of CO_2_R adsorption intermediates; discussion of hydroxyl-mediated CO_2_R pathway on SnO_2_; hydrogen evolution reaction energetics; SEIRAS spectra of Ar-saturated experiments; nonannotated SEIRAS spectra; cyclic voltammograms corresponding to SEIRAS experiments; visualization of calculated vibrations; and tabulation of calculated vibrational frequencies (PDF)
Animations of the calculated vibrations as .gif files (ZIP)
