# Supplementary material for: Insights into Electrochemical CO2 Reduction on Metallic and Oxidized Tin Using Grand-Canonical DFT and In Situ ATR-SEIRA Spectroscopy
Source: ACS Catal. 2024 May 14;14(11):8353–65. doi: 10.1021/acscatal.4c01290 (PMC11165454; doi:10.1021/acscatal.4c01290)
Supplement: Supplementary file 1 — cs4c01290_si_001.pdf [file cs4c01290_si_001.pdf]

Insights into Electrochemical CO_2 Reduction on Metallic and Oxidized Tin using Grand-Canonical DFT and *in-situ* ATR-SEIRA spectroscopy

Supplementary Information

Todd N. Whittaker[a], Yuval Fishler[a], Jacob M. Clary[b,c], Paige Brimley[a], Adam Holewinski[a], Charles B. Musgrave[a,d], Carrie A. Farberow[b,e], Wilson A. Smith[a,b*], Derek Vigil-Fowler[b,c*]

[a]Department of Chemical and Biological Engineering and Renewable and Sustainable Energy Institute, University of Colorado Boulder, Boulder, Colorado 80303, United States

[b]National Renewable Energy Laboratory, Golden, Colorado 80401, United States

[c]Materials, Chemical, and Computational Science Directorate, National Renewable Energy Laboratory, Golden, Colorado 80401, United States;

[d]Materials Science and Engineering Program, University of Colorado Boulder, Boulder, Colorado 80303, United States

[e]Catalytic Carbon Transformation and Scale-Up Center, National Renewable Energy Laboratory, Golden, Colorado 80401, United States

*Corresponding Authors: e-mail: Derek.Vigil-Fowler@nrel.gov, Wilson.Smith@nrel.gov

Table of Contents

Energy landscape of $SnO_2(110)$ in aqueous environment 3
Importance of GC-DFT for adsorption energies 4
Additional CO_2R intermediate considerations 5
Hydrogen Evolution Reaction Energetics 7
Visualization of Vibrational Frequencies 9
Additional CO_2R SEIRAS information 33
Evidence for interfacial buffering by $SnO_2(110)$ 35
SEIRAS in the absence of CO_2 36
Hydroxyl-mediated CO_2R on SnO_2 37
References 42

CO_2R Adsorption Intermediate Geometries

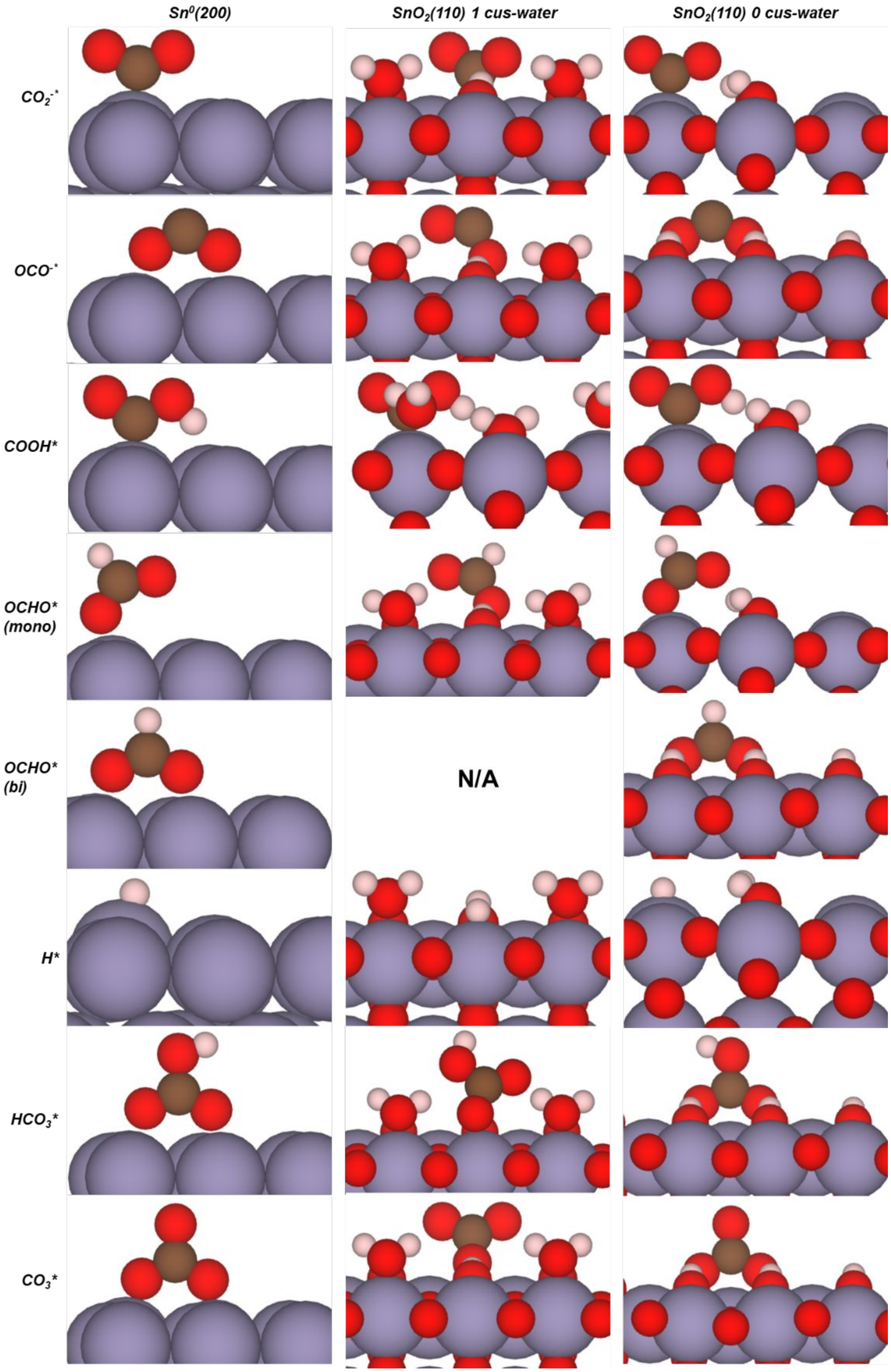

Figure S.1. Converged geometries of the CO_2R intermediates considered at -0.5 V_{RHE} across the three Sn surface.

Energy landscape of SnO_2(110) in aqueous environment

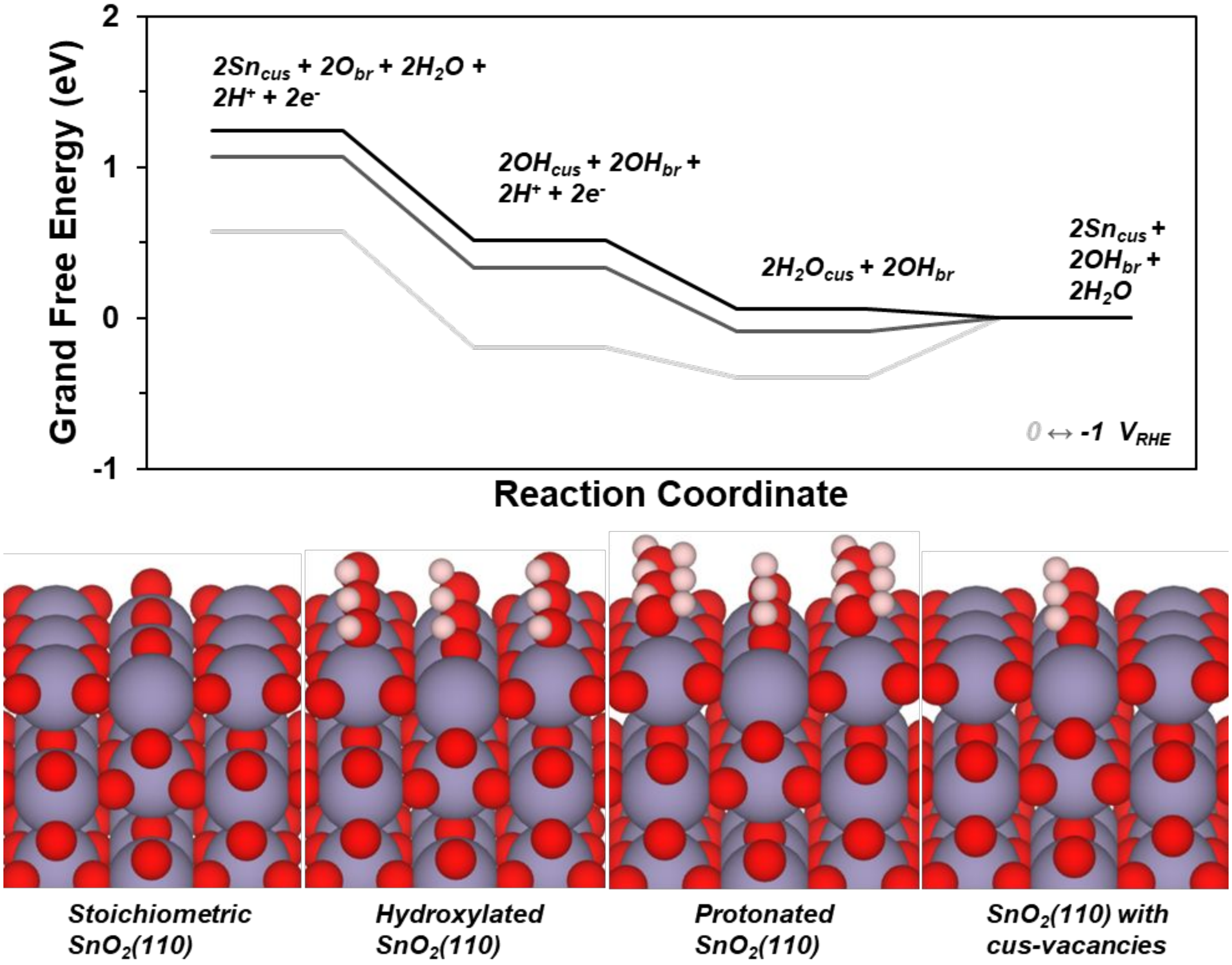

Figure S.2. (top) Reaction coordinate diagram showing the energetics of different degrees of SnO_2(110) hydroxylation. (bottom) SnO_2(110) structures corresponding to the different states in the above reaction coordinate diagram.

Figure S.2 shows the free energy landscape of SnO_2(110), specifically for the interaction with water. SnO_2(110) spontaneously dissociates water into cus-hydroxyls and br-hydroxyls at all potentials. Furthermore, the hydroxylated SnO_2(110) is spontaneously protonated at the cus-hydroxyl sites to form cus-water at all potentials. The cus-waters can be reductively desorbed at potentials more negative than -0.8 V_{RHE} to re-form the naked cus-Sn sites. For CO_2R, we considered the SnO_2(110) with all br- sites as br-hydroxyls and either one cus-water and one cus-Sn or two cus-Sn sites (shown as the far right structure in **Figure S.2**).

Importance of GC-DFT for adsorption energies

As we described in the introduction of the main text, GC-DFT is necessary to capture the energetics of partial charge transfer steps or decoupled electron/proton transfers. For CO_2R, the main step that this is relevant for is the reductive adsorption of CO_2 ($CO_2 + * + e^- \rightarrow CO_2^{-*}$). **Figure S.3** and **Figure S.4** show the initial structure for the adsorption of CO_2^{-*} and OCO^{-*} as well as the converged structures at 0, -0.5 and -1 V_{RHE} for all three Sn surfaces considered. The canonical, zero charge structure is also shown. First, it is apparent that, at low potential, the CO_2 spontaneously desorbs and there is no electron transfer. It is not until the potential is made more negative that an additional electron is added and the adsorption of CO_2^{-*} is an accessible state (even if it is still calculated to be uphill thermodynamically). This shows the importance of using GC-DFT for computational electrochemistry; without it the nuance of adsorption energetics as a function of potential may be lost. For the adsorption energetics shown in **Figure 3**, we constrained the Sn-C or Sn-O bonds to prevent spontaneous desorption accurately represent the potential dependence of adsorption.

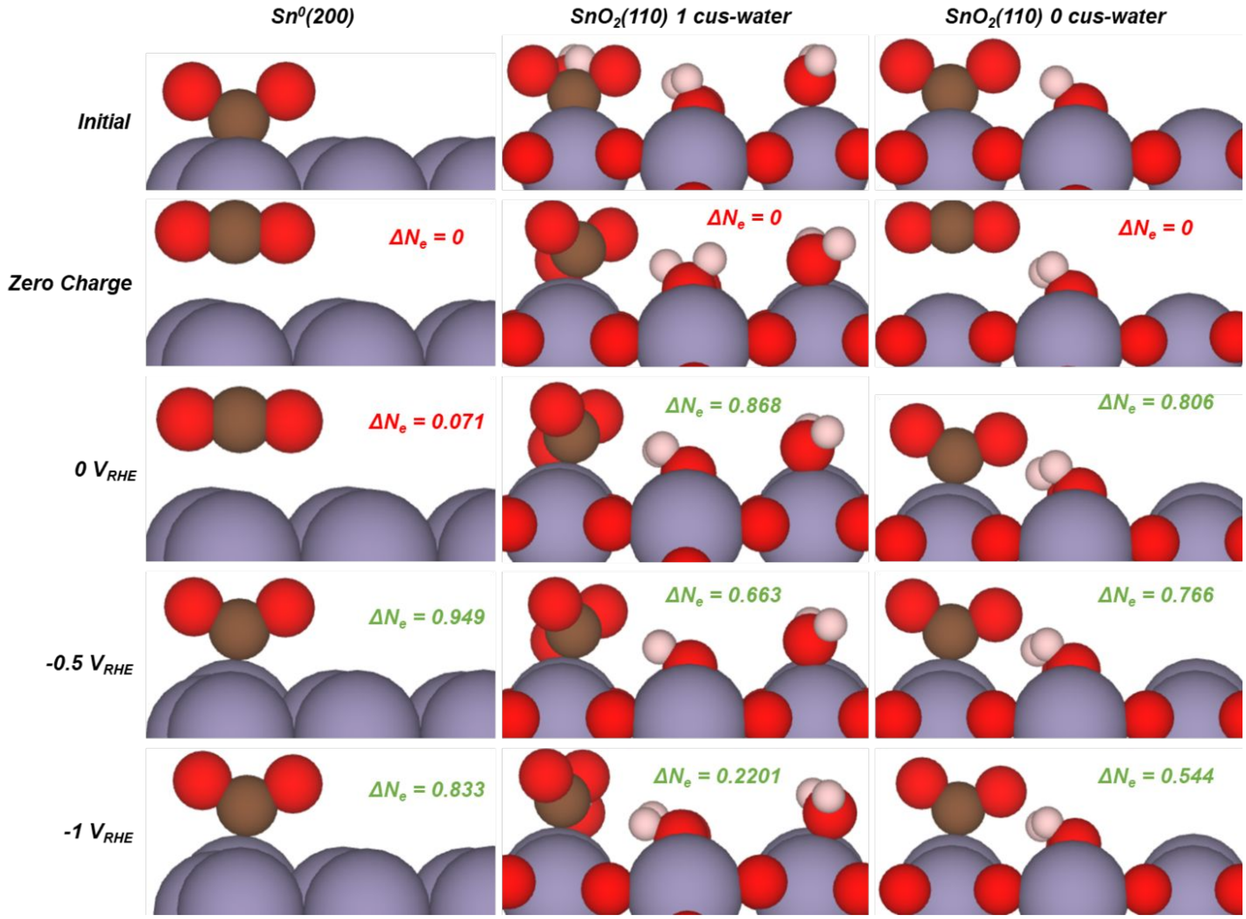

Figure S.3. GC-DFT adsorption geometries for CO_2^{-*}. Top row is the initial guess for each calculation, second row is the converged canonical, zero-charge structure and the remaining rows are the converged grand-canonical structures at 0 (third row), -0.5 (fourth row) and -1 (fifth row) V_{RHE}. The change in the number of electrons is also shown.

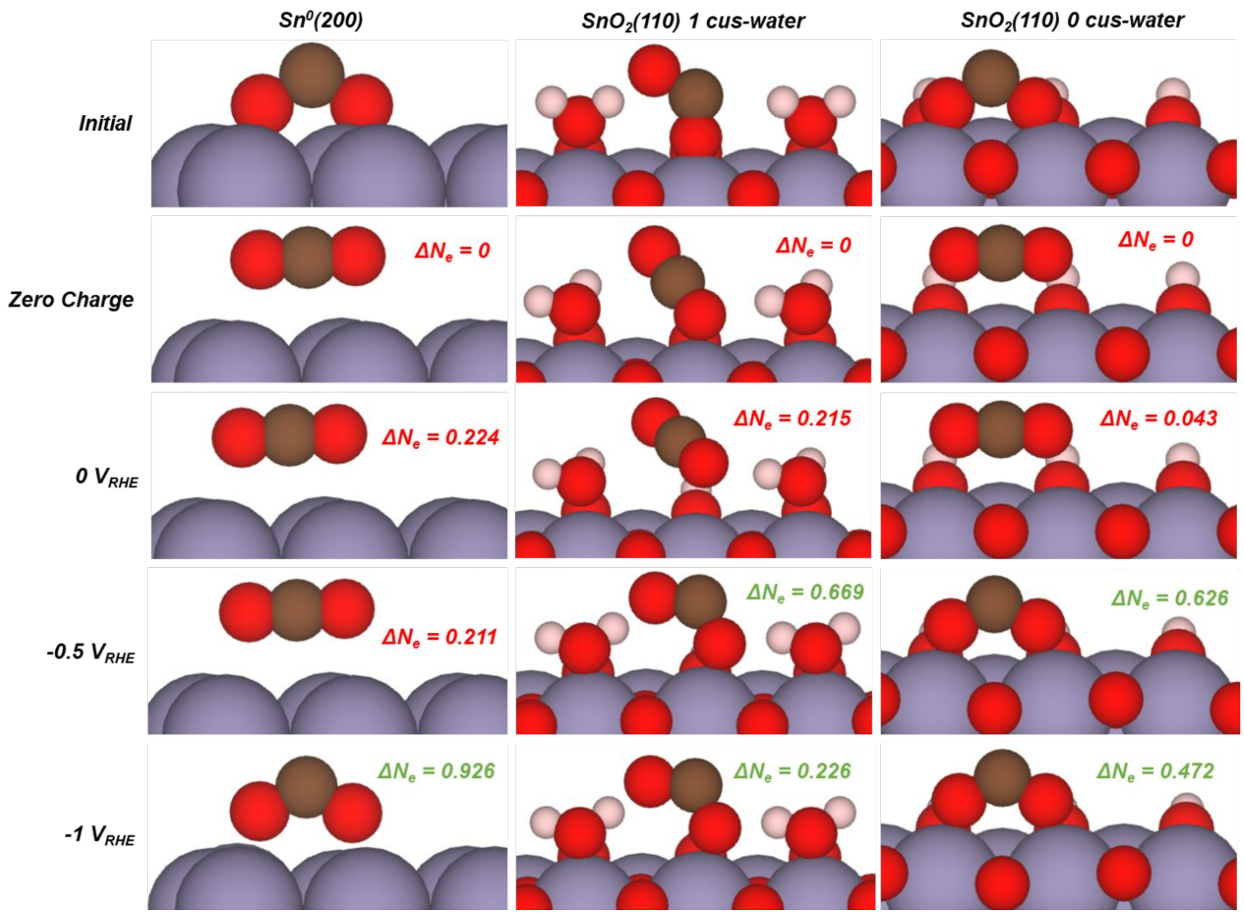

Figure S.4. GC-DFT adsorption geometries for OCO^{-*}. Top row is the initial guess for each calculation, second row is the converged canonical, zero-charge structure and the remaining rows are the converged grand-canonical structures at 0 (third row), -0.5 (fourth row) and -1 (fifth row) V_{RHE}. The change in the number of electrons is also shown.

Additional CO_2R intermediate considerations

We also considered the formation of surface bound CO and formic acid (HCOOH) as mechanistically relevant intermediates. However, as shown in **Figures S.5** and **S.6**, the Sn-C and Sn-O bonds spontaneously elongate to greater than 3 Å at all potentials on all three Sn surfaces considered, which is longer than what would typically be considered chemisorbed.

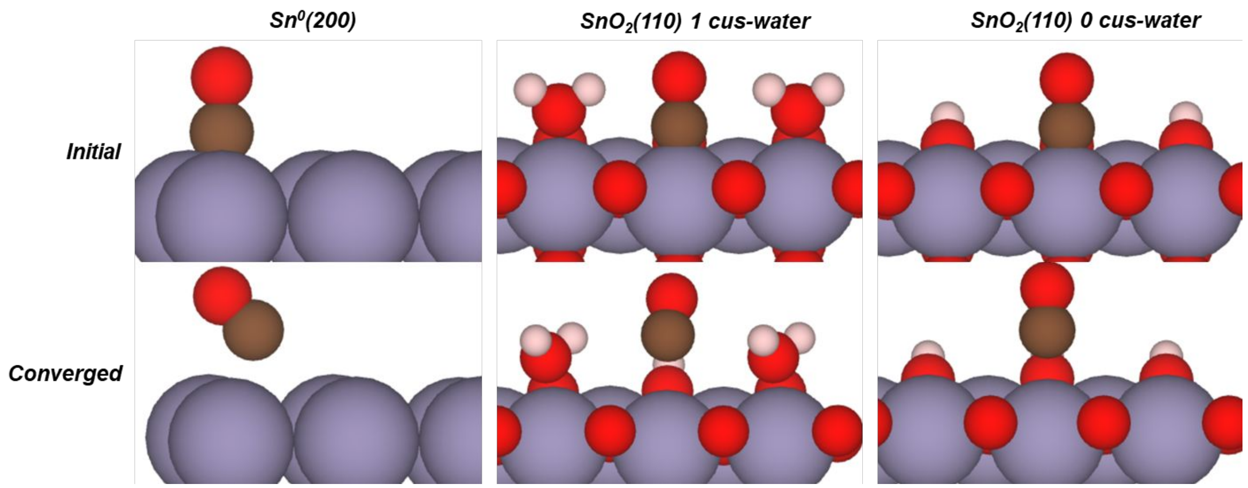

Figure S.5. GC-DFT adsorption geometries for CO. Top row is the initial guess for each calculation, second row is the converged structure (all of the potentials converged to a similar desorbed state).

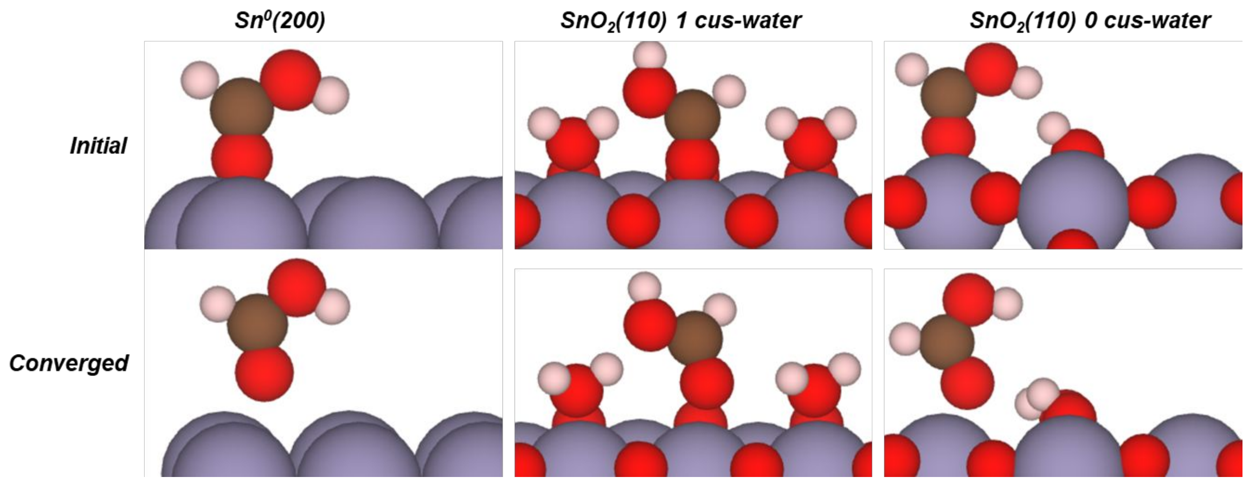

Figure S.6. GC-DFT adsorption geometries for formic acid. Top row is the initial guess for each calculation, second row is the converged structure (all of the potentials converged to a similar desorbed state).

We considered the formation of HCOOH via Langmuir-Hinshelwood coupling of COOH* and H*. The energetics are shown in **Figure S.7.** By looking at **Figure 3** in the main text, the number of electrons associated with the formation of COOH* and H* sums to greater than 2, which explains the net oxidation calculated for the step shown in **Figure S.7**. Therefore, this step becomes less favorable as the potential is made more negative, and this step is not considered further as a relevant process.

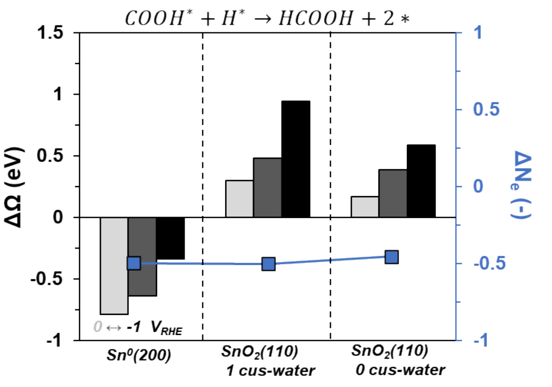

Figure S.7. Change in grand free energy as a function of potential for the formation of formic acid via the Langmuir-Hinshelwood coupling of COOH* and H*. The potentials are 0 (light grey), -0.5 (dark grey) and -1 (black) V_{RHE}. The average number of electrons transferred across the three potentials is also shown (in blue).

Hydrogen Evolution Reaction Energetics

We calculated the free energy changes for the two prominent hydrogen evolution reaction (HER) pathways to assess its competitiveness with CO_2R. In particular, we wanted to evaluate the driving force for formation of CO_2R intermediates through the Eley-Rideal reaction between CO_2 and H* vs H* reacting to form H_2 (via a Heyrovsky or Tafel step). **Figure S.8** shows the reaction coordinate diagrams for both the Volmer-Heyrovsky and Volmer-Tafel mechanism on all three Sn surfaces. The formation of H_2 via the Heyrovsky step is downhill at all potentials on all surfaces, so unless there is a significant kinetic barrier for this step, the coverage of H* is expected to be fairly low.

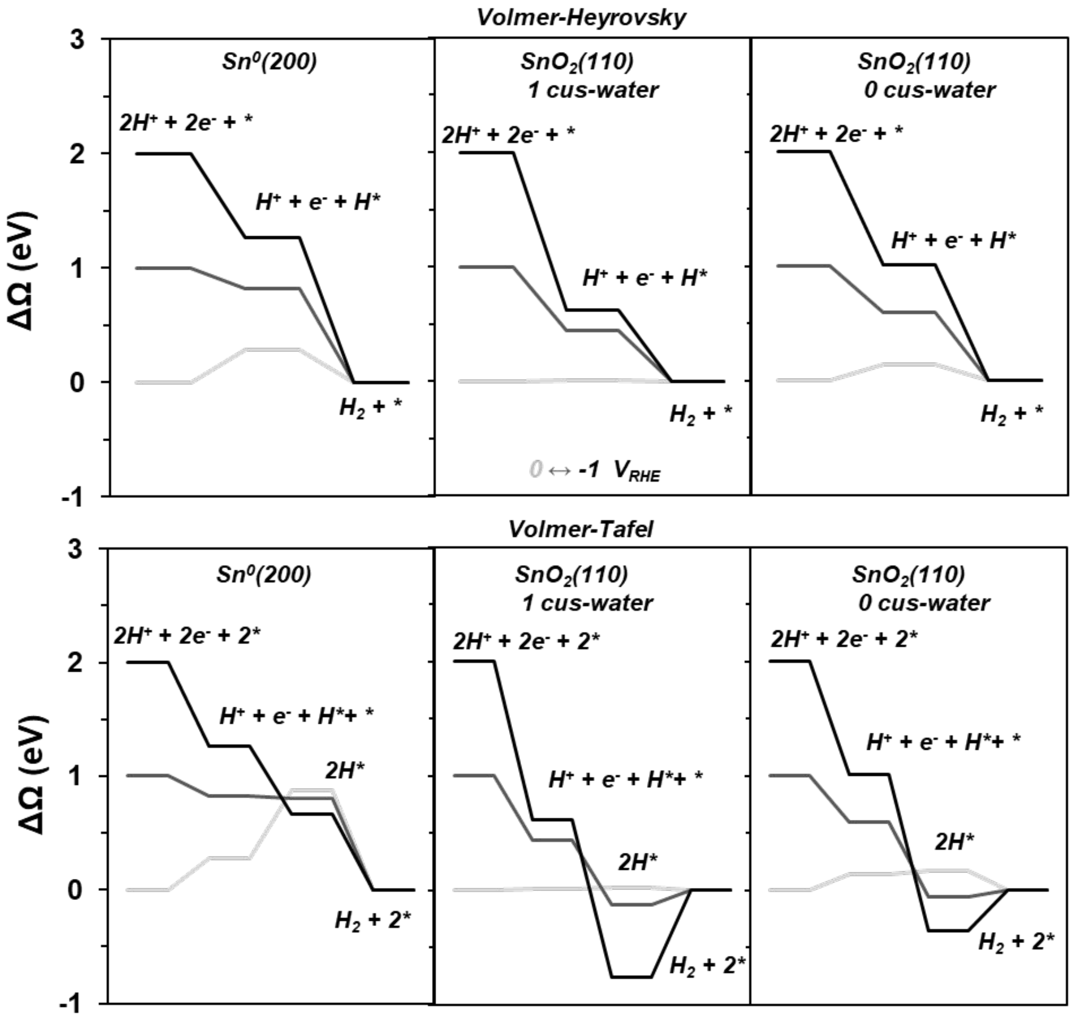

Figure S.8. Reaction coordinate diagram for the Volmer-Heyrovsky (top) and Volmer-Tafel (bottom) mechanisms of HER on metallic Sn (left), SnO_2 with one cus-water (middle) and no cus-waters (right) as a functional of potential. The potentials are 0 (light grey), -0.5 (dark grey) and -1 (black) V_{RHE}.

Additional carbonate adsorption consideration

As mentioned in the main text, we also considered the formation of carbonate on the surface via the simultaneous adsorption and discharge of proton from bicarbonate. The energetics for this process are shown in **Figure S.9**. Comparing the energetics of this process to the energetics shown in the main text (**Figure 5**), this process is much less favorable than either molecular adsorption of bicarbonate or carbonate, so it is more likely that surface-bound carbonate is formed via molecular adsorption of carbonate formed in solution from an increase in interfacial pH.

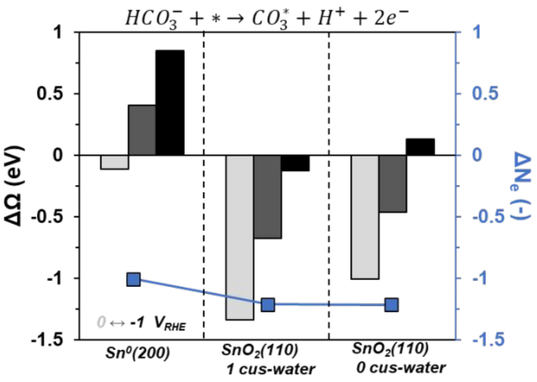

Figure S.9. Change in grand free energy as a function of potential for the formation of surface-bound carbonate via simultaneous adsorption and discharge of proton from bicarbonate. The potentials are 0 (light grey), -0.5 (dark grey) and -1 (black) V_{RHE}. The average number of electrons transferred across the three potentials is also shown (in blue).

Visualization of Vibrational Frequencies

Due to the surface selection rule, only vibrational modes that have a change in the dipole moment perpendicular to the surface will have an observable ATR-SEIRAS peak. Therefore, the vibrational modes need to be visualized to determine if they have a change in dipole in the z-direction. We chose to only visualize vibrational modes with frequencies above 900 cm^{-1} because the so-called fingerprint region (< 1000 cm^{-1}) is notoriously difficult to analyze with many overlapping peaks. Additionally, we were most interested in explaining the difference in the observed peaks for metallic and oxidic Sn and consolidating that with the observed activity difference in the context of our computed reaction energetics. All reported vibrational frequencies in **Figures S.10-33** are at -0.5 V_{RHE} (other potentials are reported below in **Table S.1**). We also include animated gifs of the vibrations that are available for download to assist in visualization.

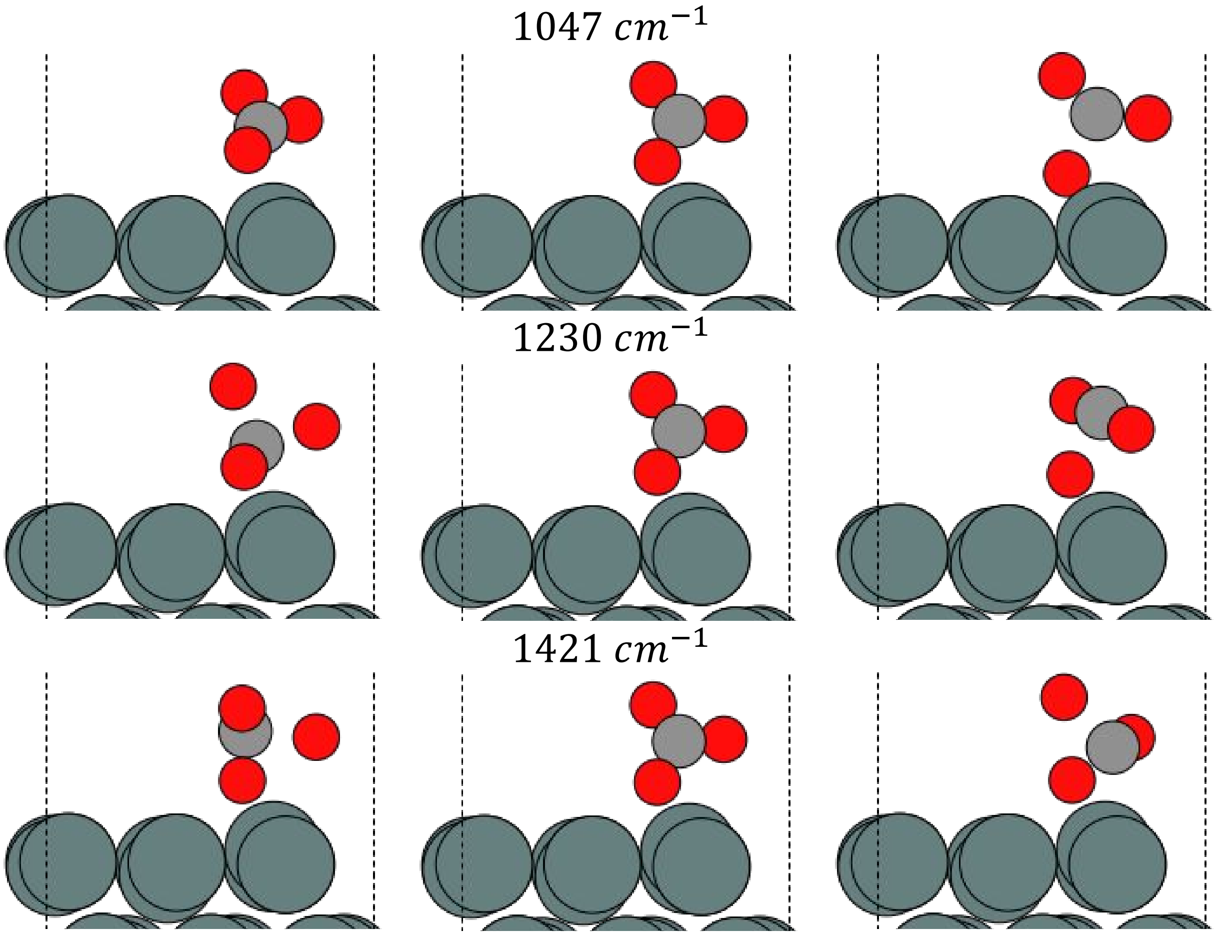

Figure S.10. Visualization of vibrational modes for monodentate CO_3 on metallic Sn

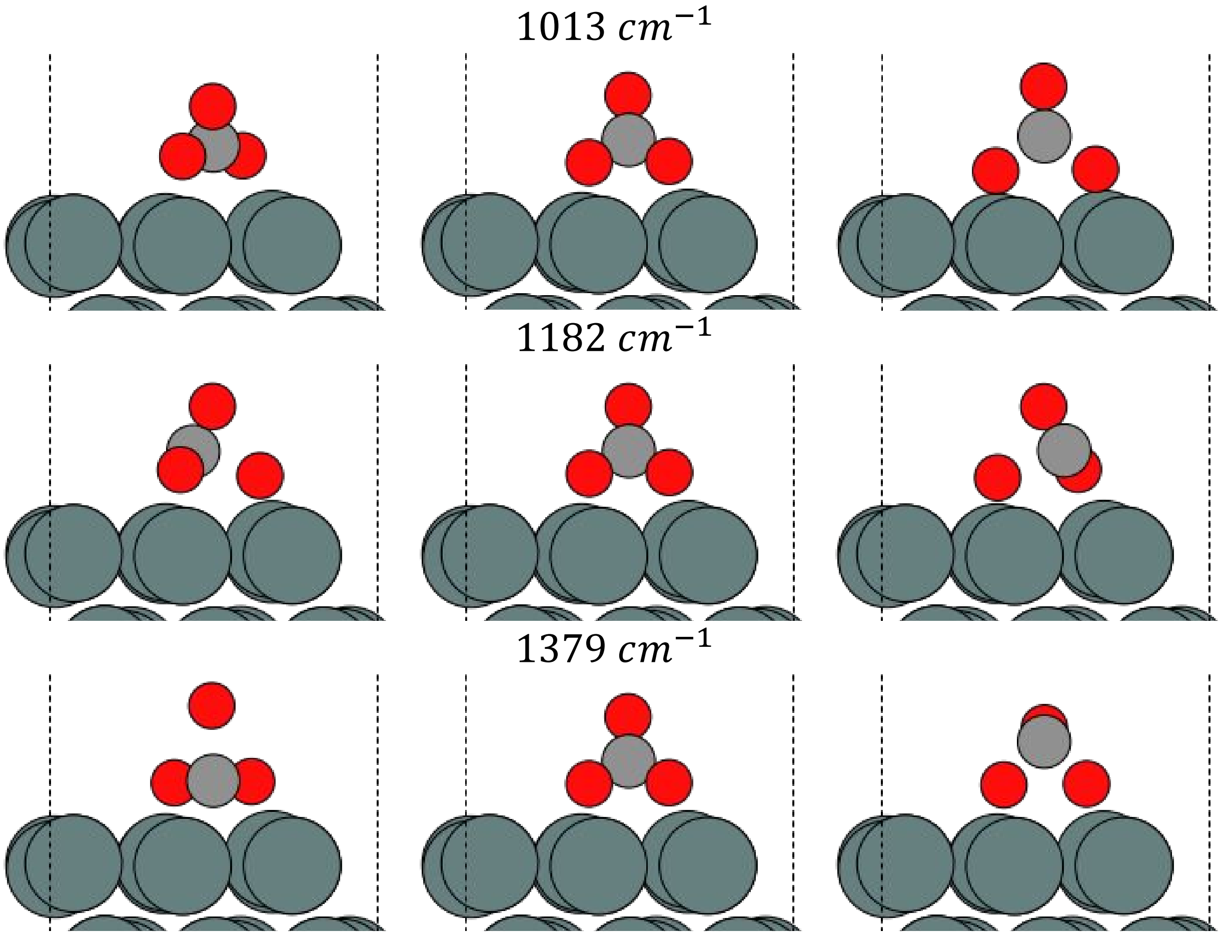

Figure S.11. Visualization of vibrational modes for bidentate CO_3 on metallic Sn.

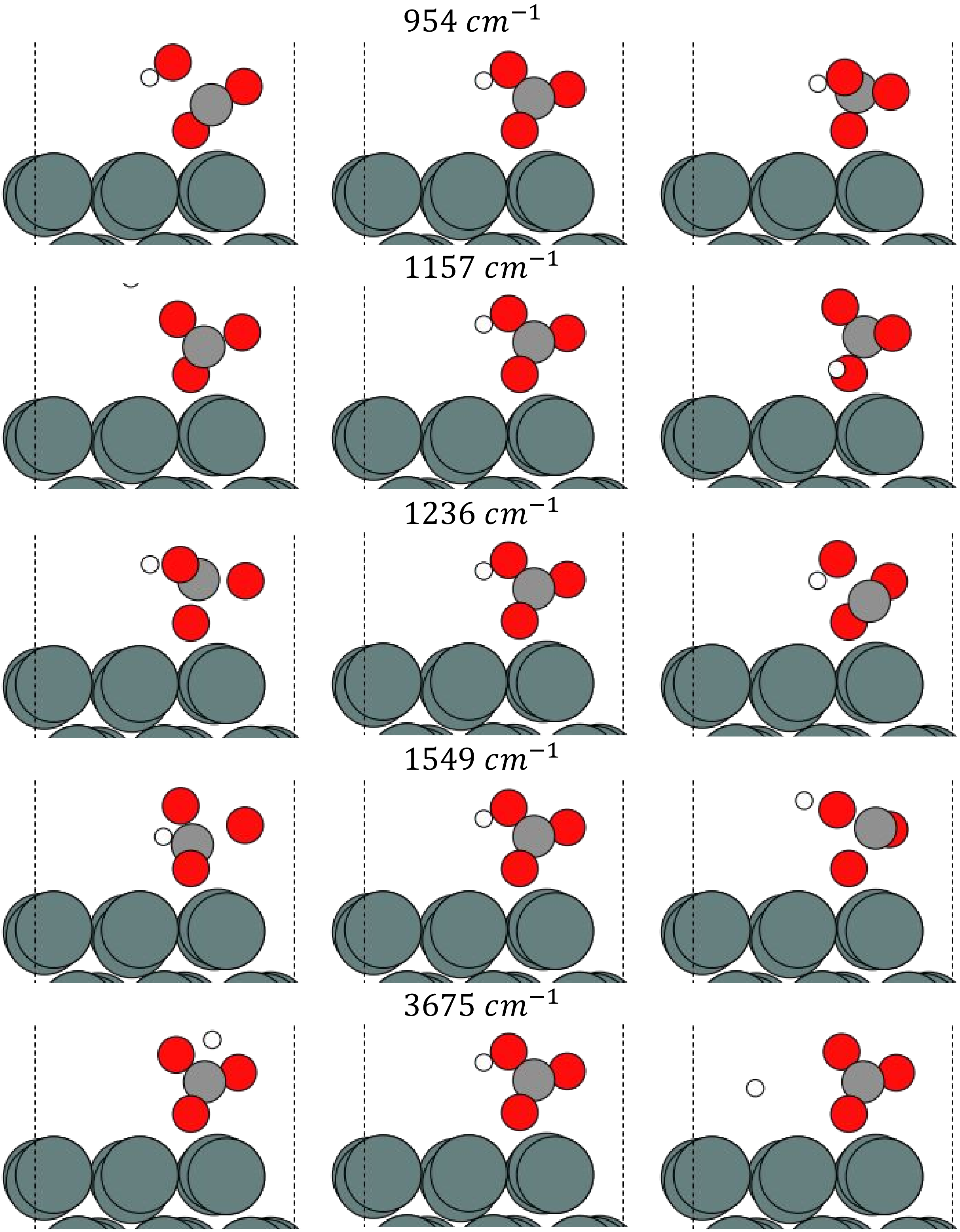

Figure S.12. Visualization of vibrational modes for monodentate HCO_3 on metallic Sn

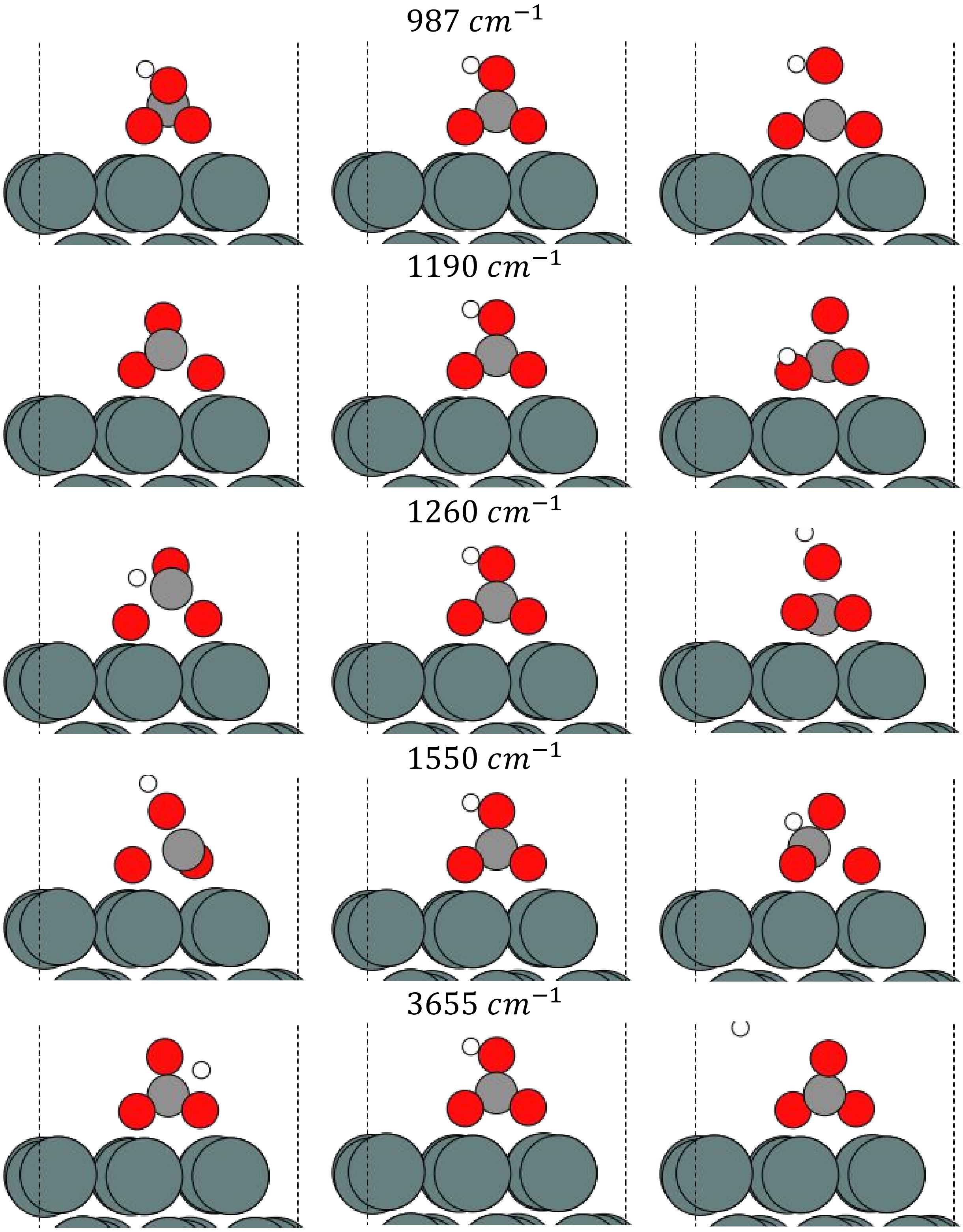

Figure S.13. Visualization of vibrational modes for bidentate HCO_3 on metallic Sn

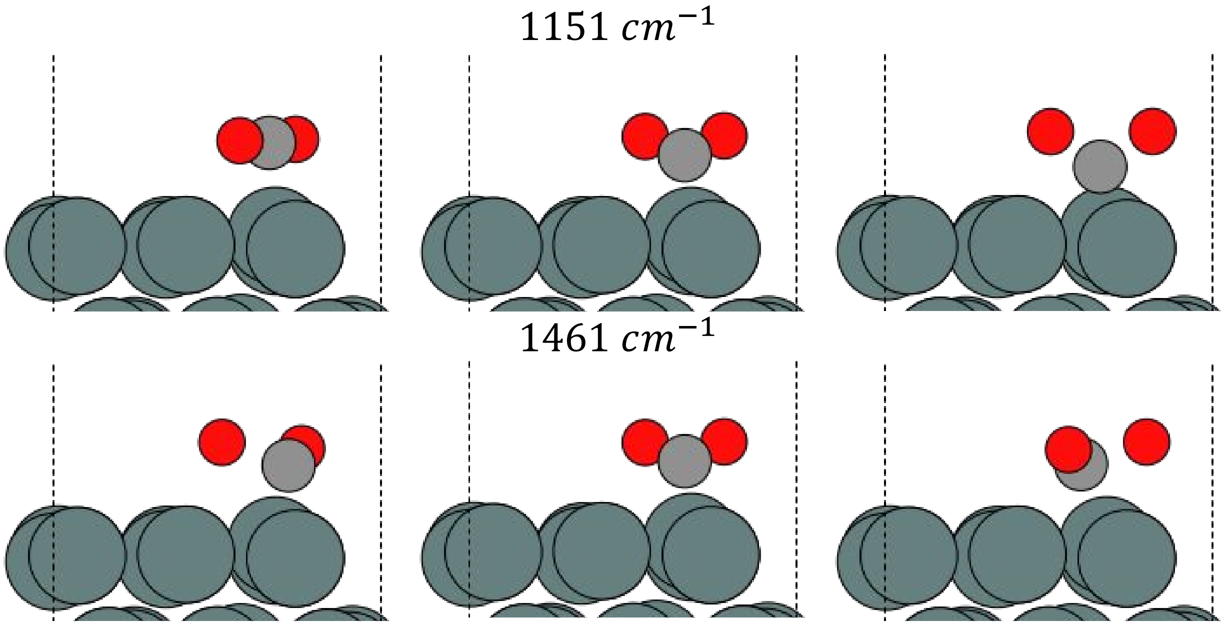

Figure S.14. Visualization of vibrational modes for CO_2 bound through carbon on metallic Sn

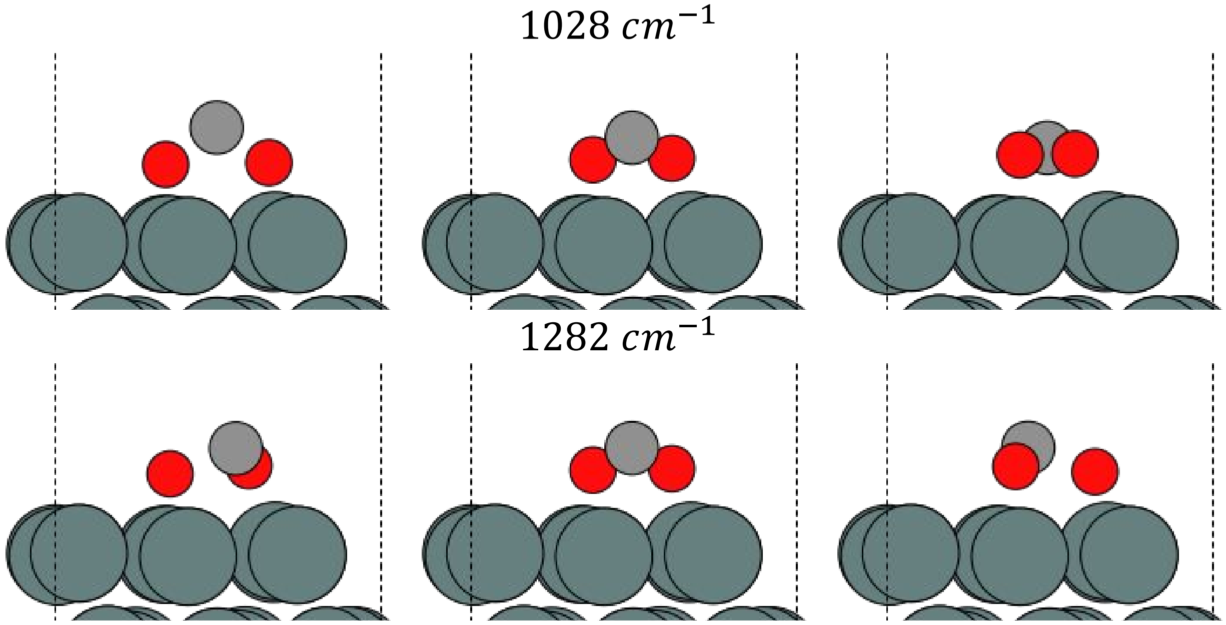

Figure S.15. Visualization of vibrational modes for CO_2 bound through oxygens on metallic Sn. Note, these are the vibrations at -1 V_{RHE} due to CO_2 spontaneously desorbing at -0.5 V_{RHE} for this structure.

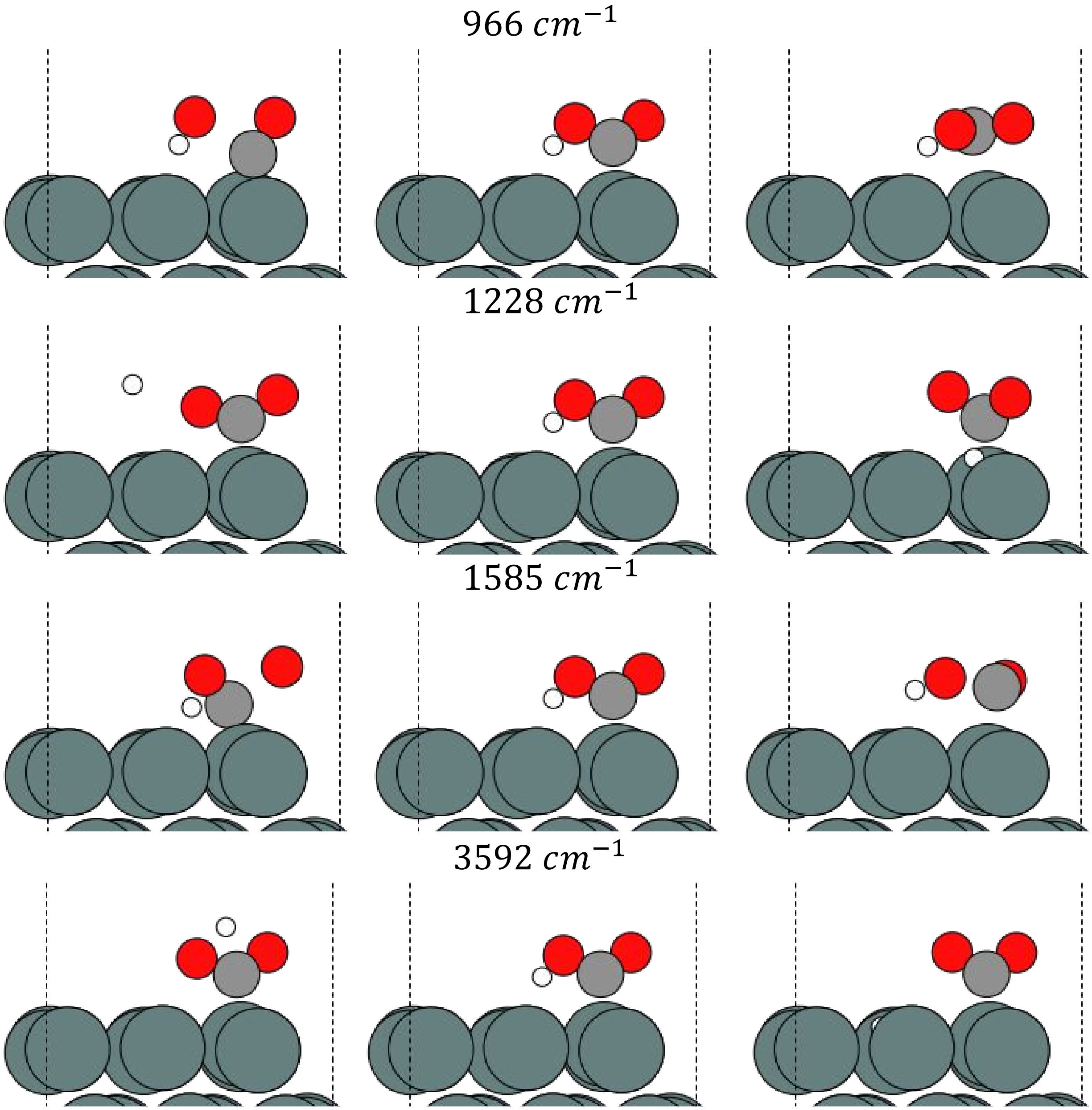

Figure S.16. Visualization of vibrational modes for COOH on metallic Sn

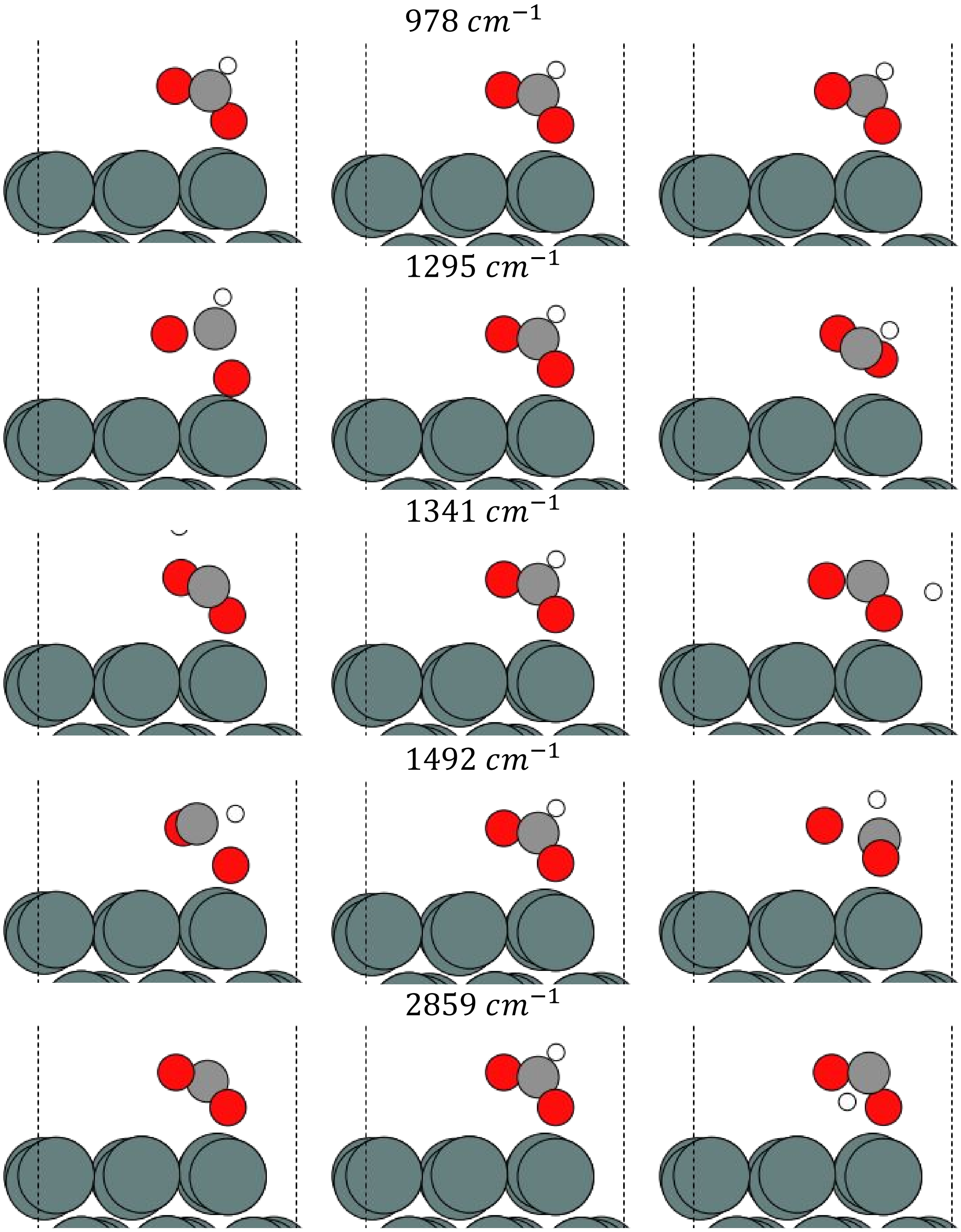

Figure S.17. Visualization of vibrational modes for monodentate OCHO on metallic Sn

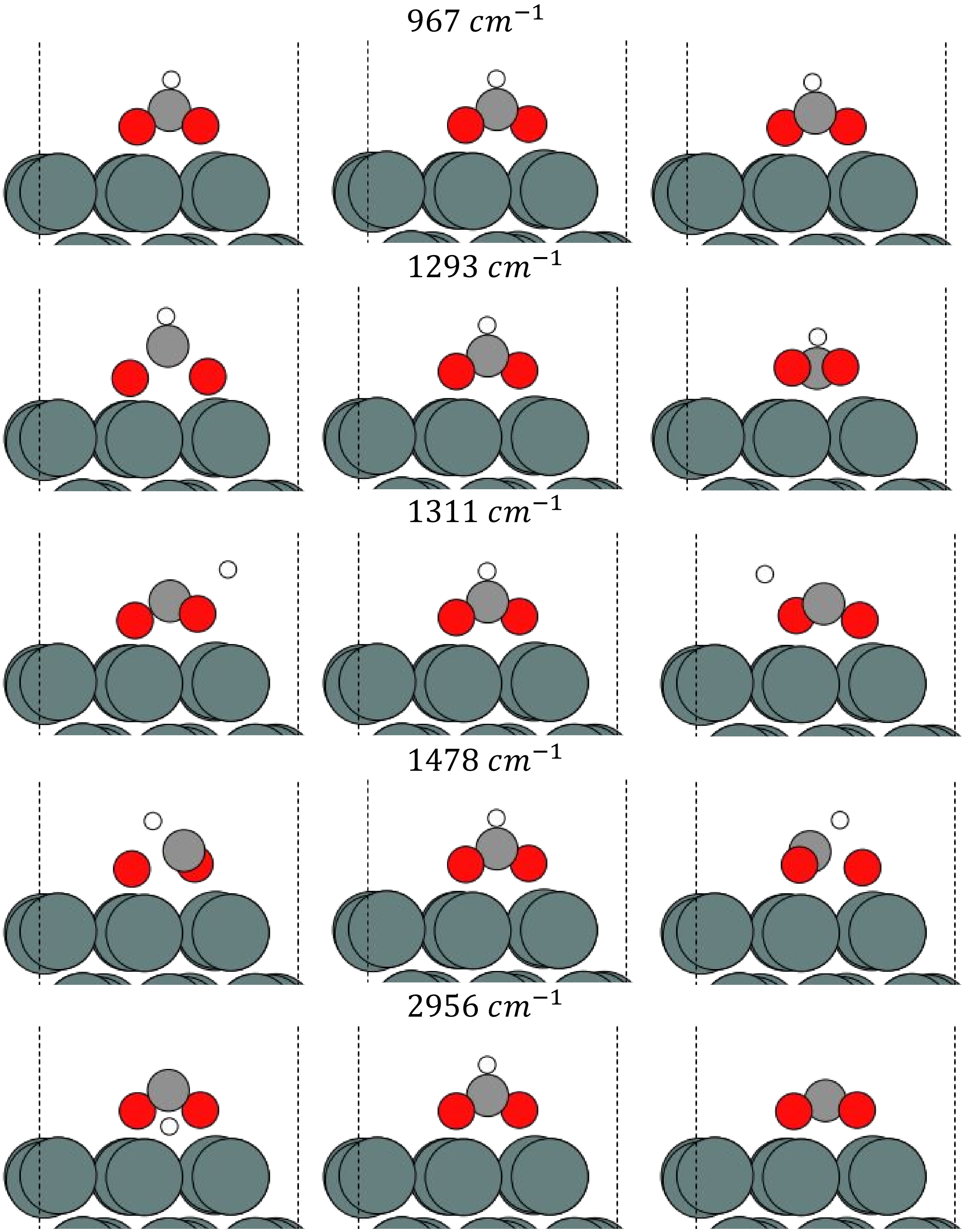

Figure S.18. Visualization of vibrational modes for bidentate OCHO on metallic Sn

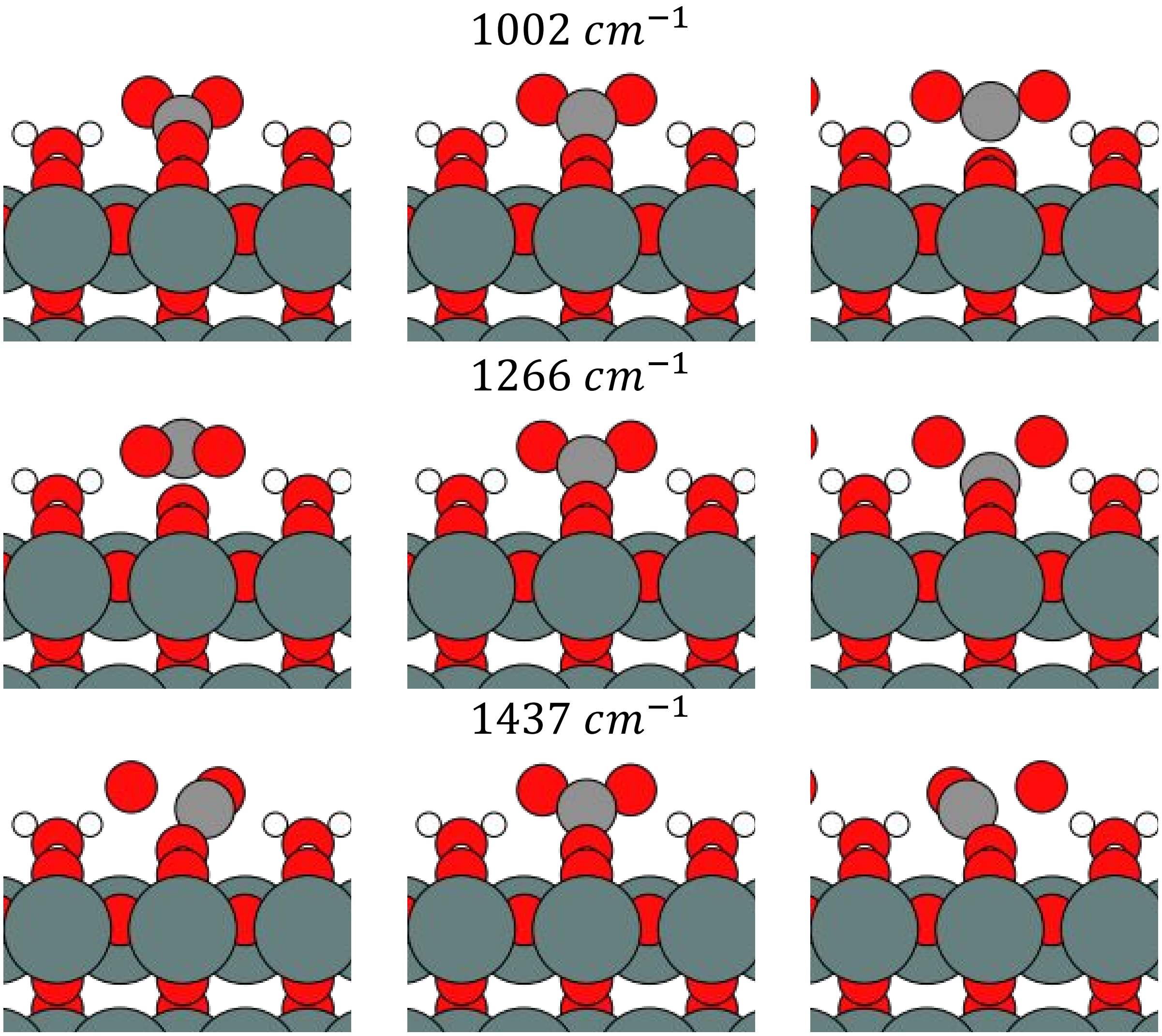

Figure S.19. Visualization of vibrational modes for monodentate CO_3 on SnO_2 with one cus-water and one cus-vacancy

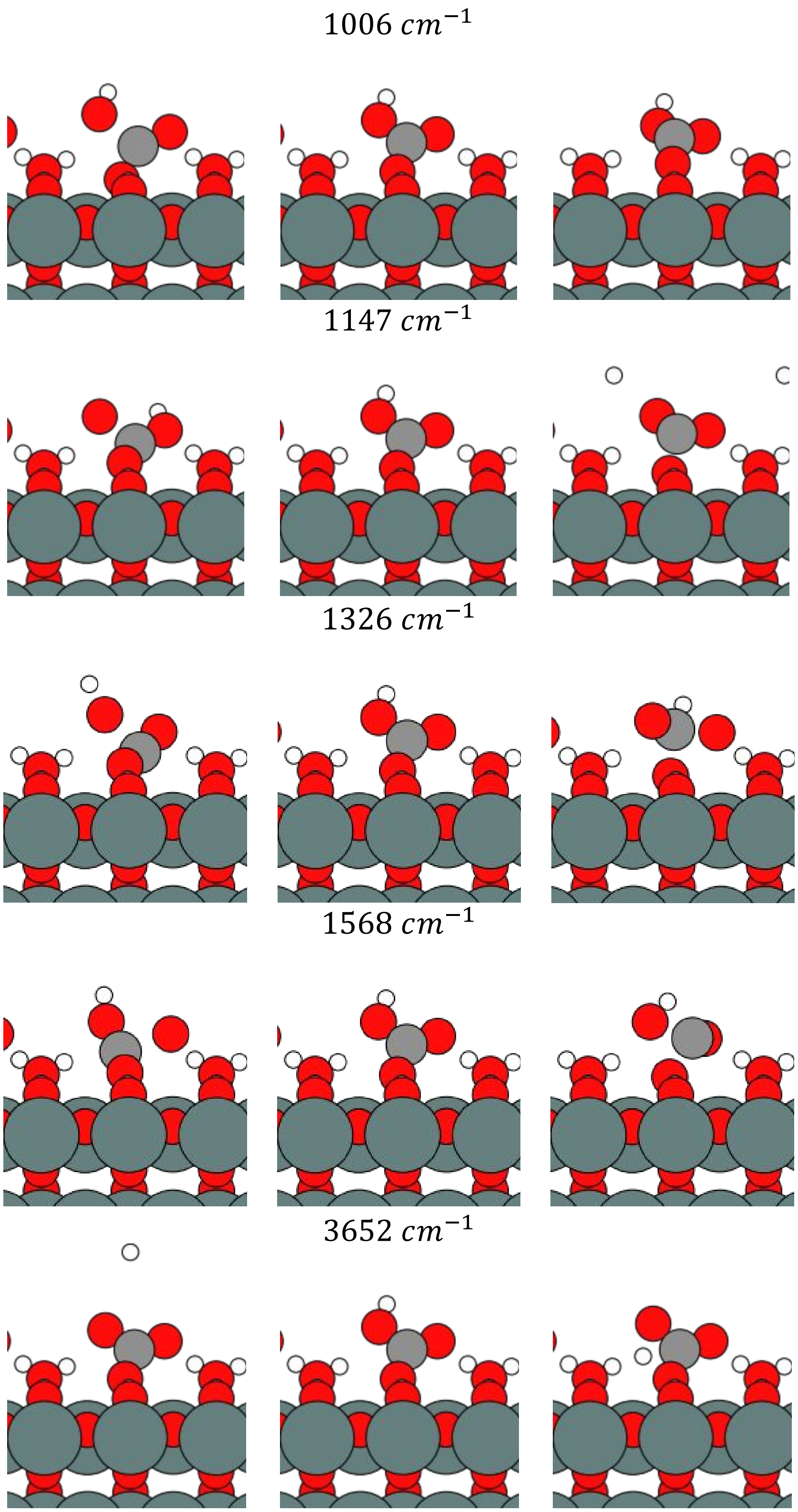

Figure S.20. Visualization of vibrational modes for monodentate HCO_3 on SnO_2 with one cus-water and one cus-vacancy

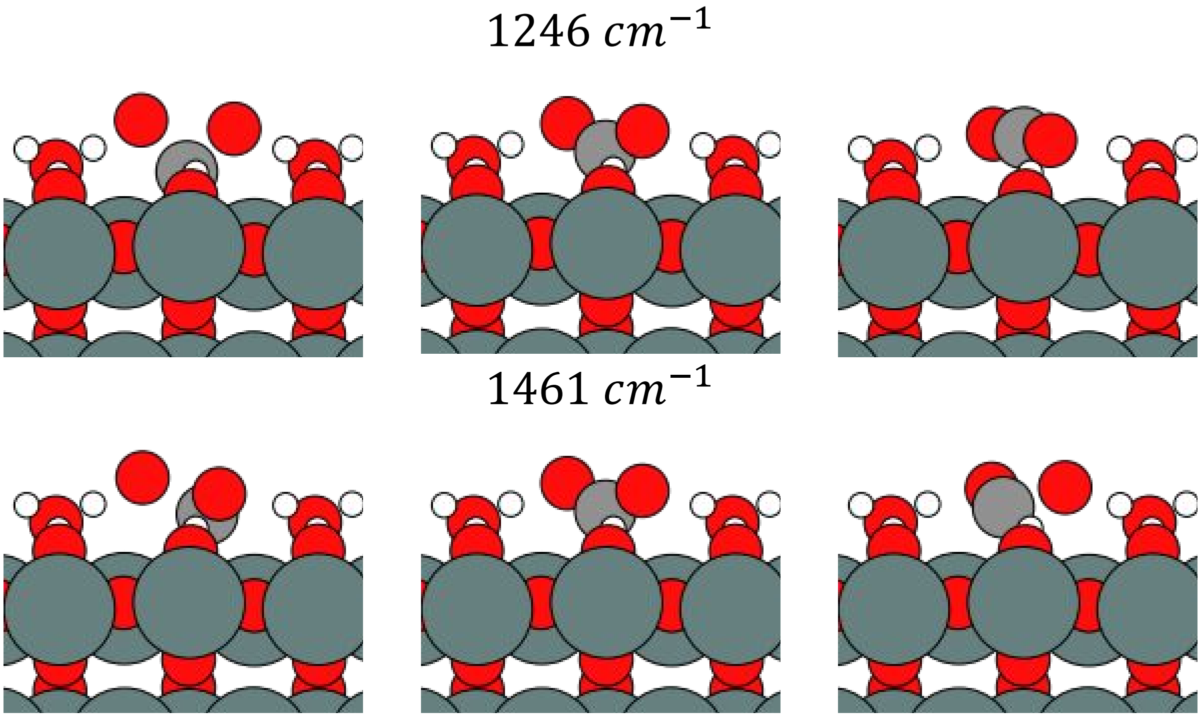

Figure S.21. Visualization of vibrational modes for CO_2 bound through carbon on SnO_2 with one cus-water and one cus-vacancy

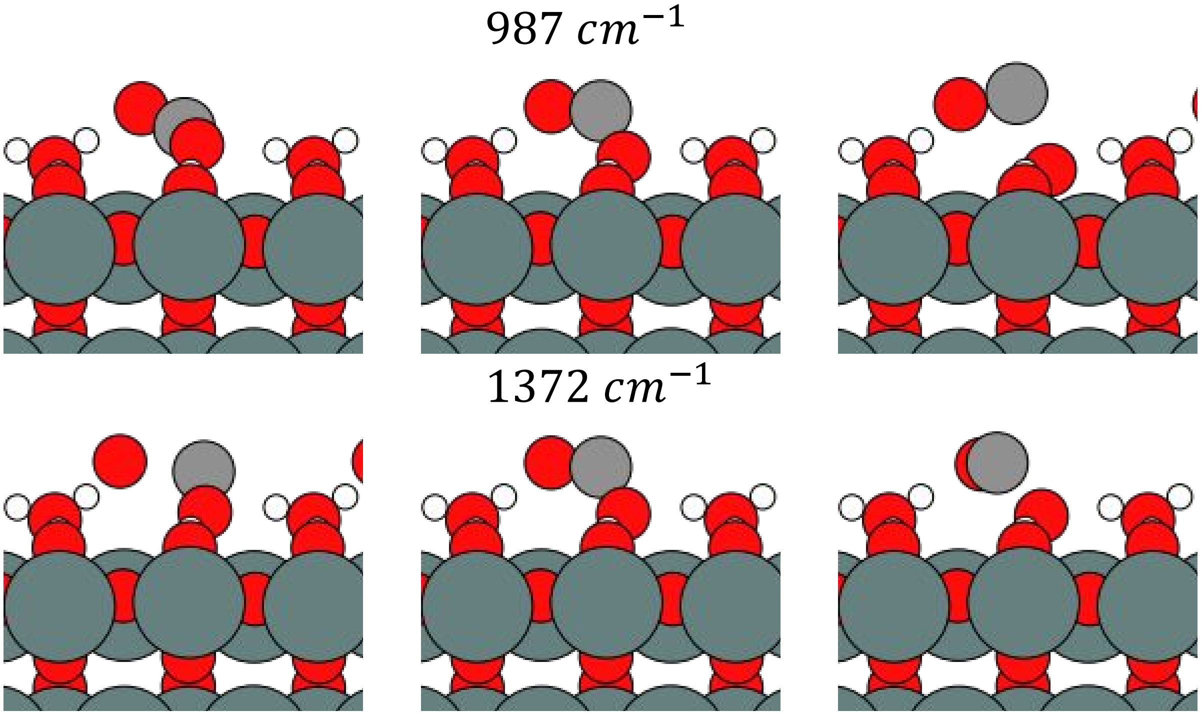

Figure S.22. Visualization of vibrational modes for CO_2 bound through oxygen on SnO_2 with one cus-water and one cus-vacancy

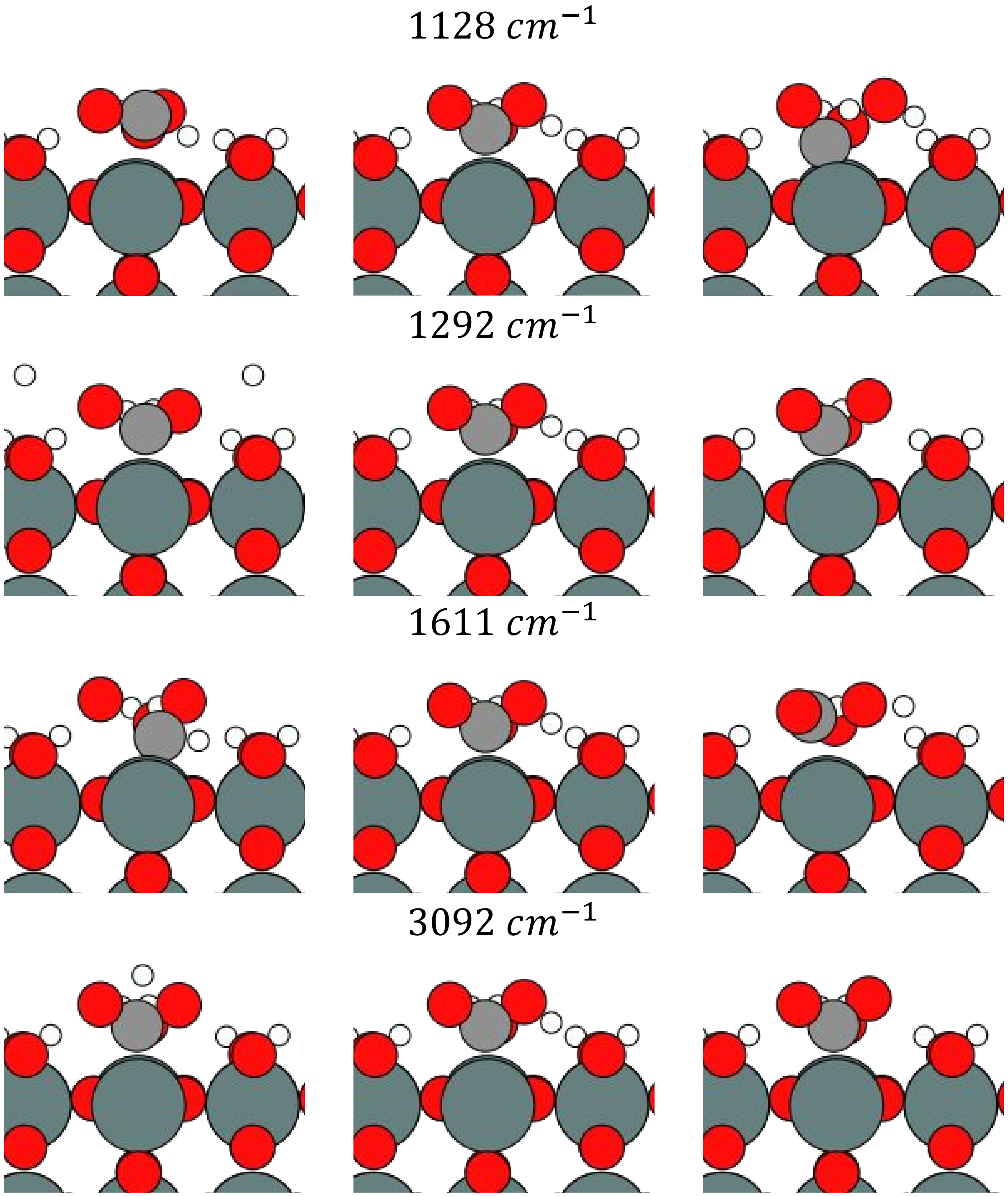

Figure S.23. Visualization of vibrational modes for COOH on SnO_2 with one cus-water and one cus-vacancy

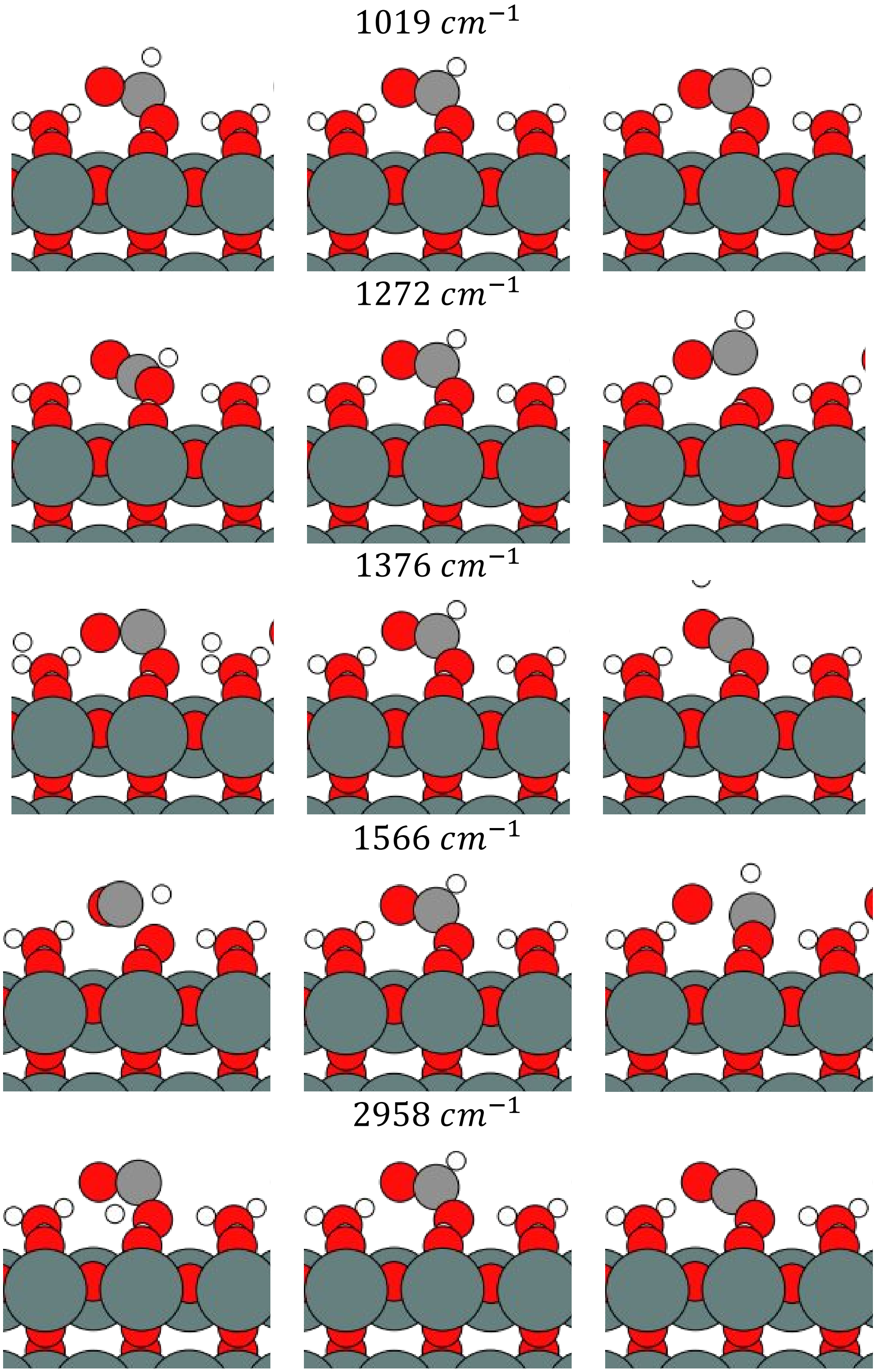

Figure S.24. Visualization of vibrational modes for monodentate OCHO on SnO_2 with one cus-water and one cus-vacancy

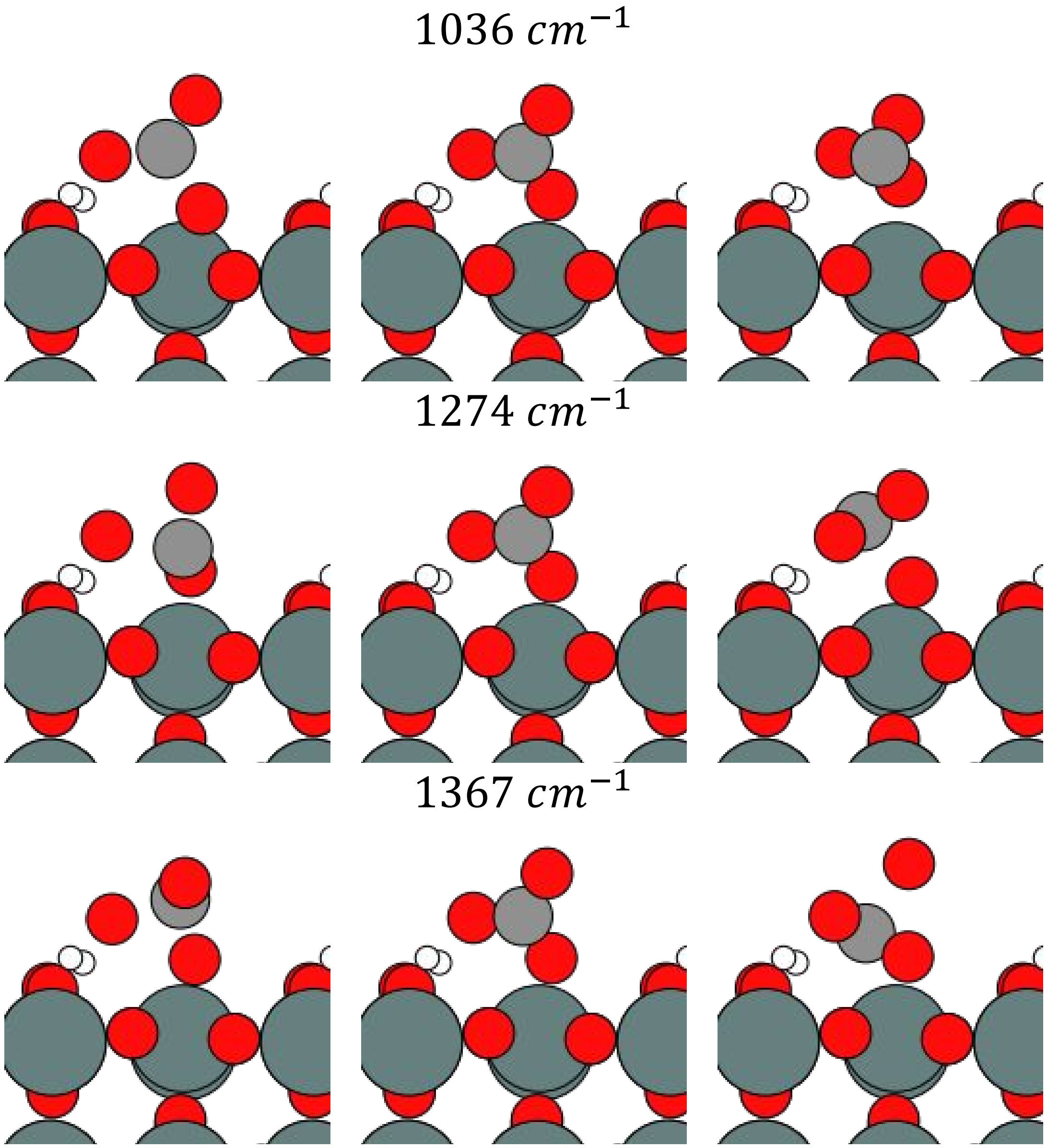

Figure S.25. Visualization of vibrational modes for monodentate CO_3 on SnO_2 with no cus-water and two cus-vacancies

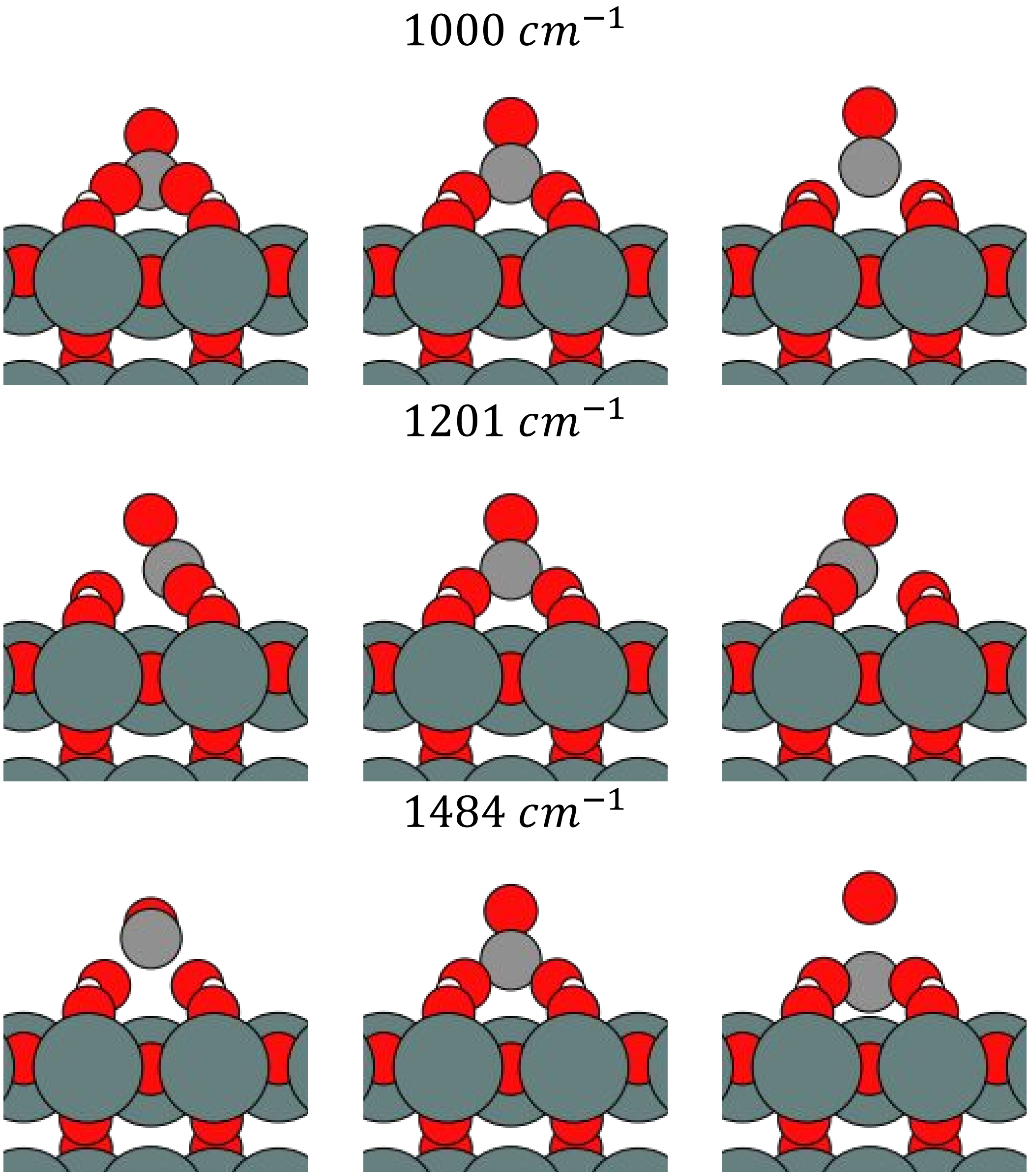

Figure S.26. Visualization of vibrational modes for bidentate CO_3 on SnO_2 with no cus-water and two cus-vacancies

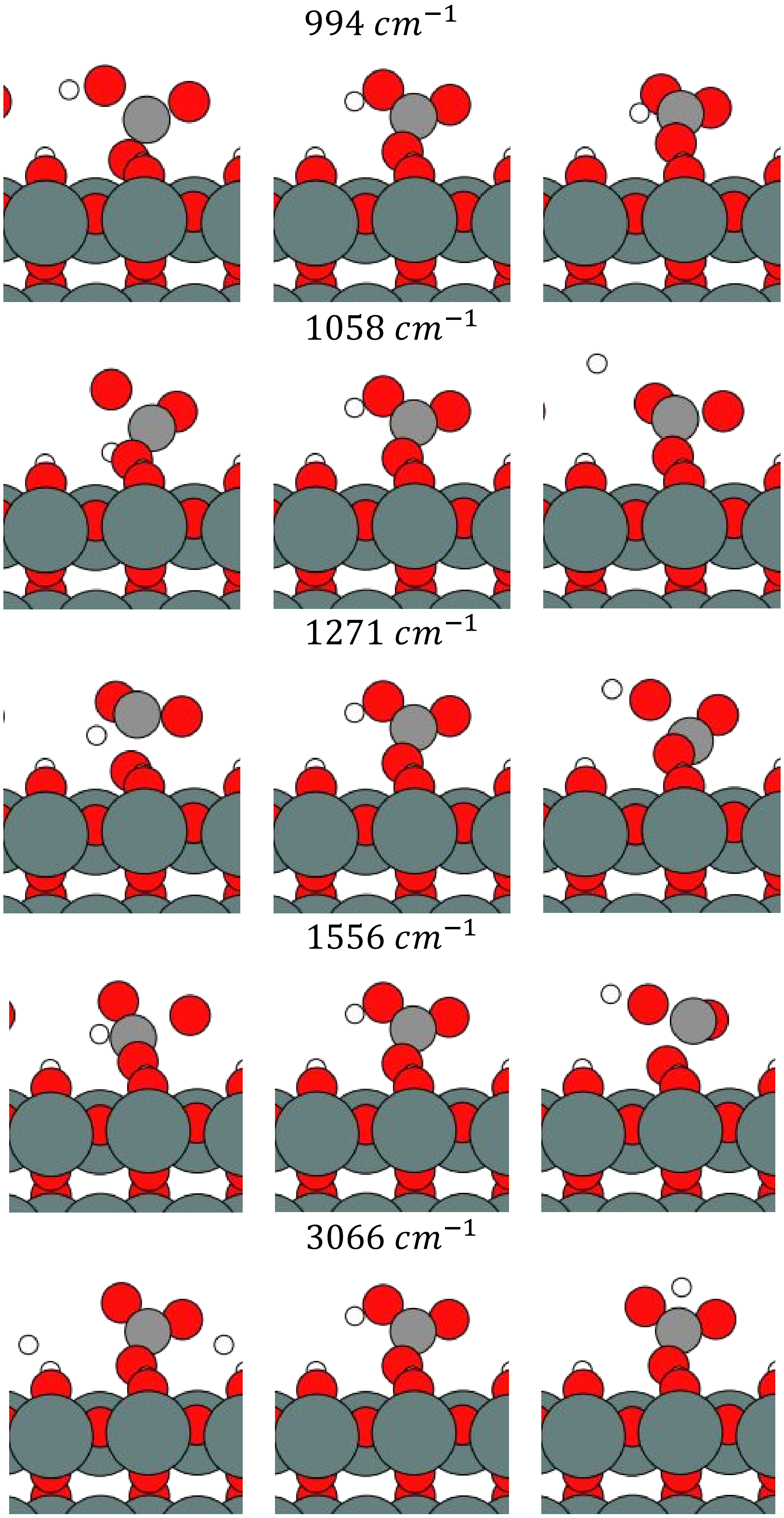

Figure S.27. Visualization of vibrational modes for monodentate HCO_3 on SnO_2 with no cus-water and two cus-vacancies

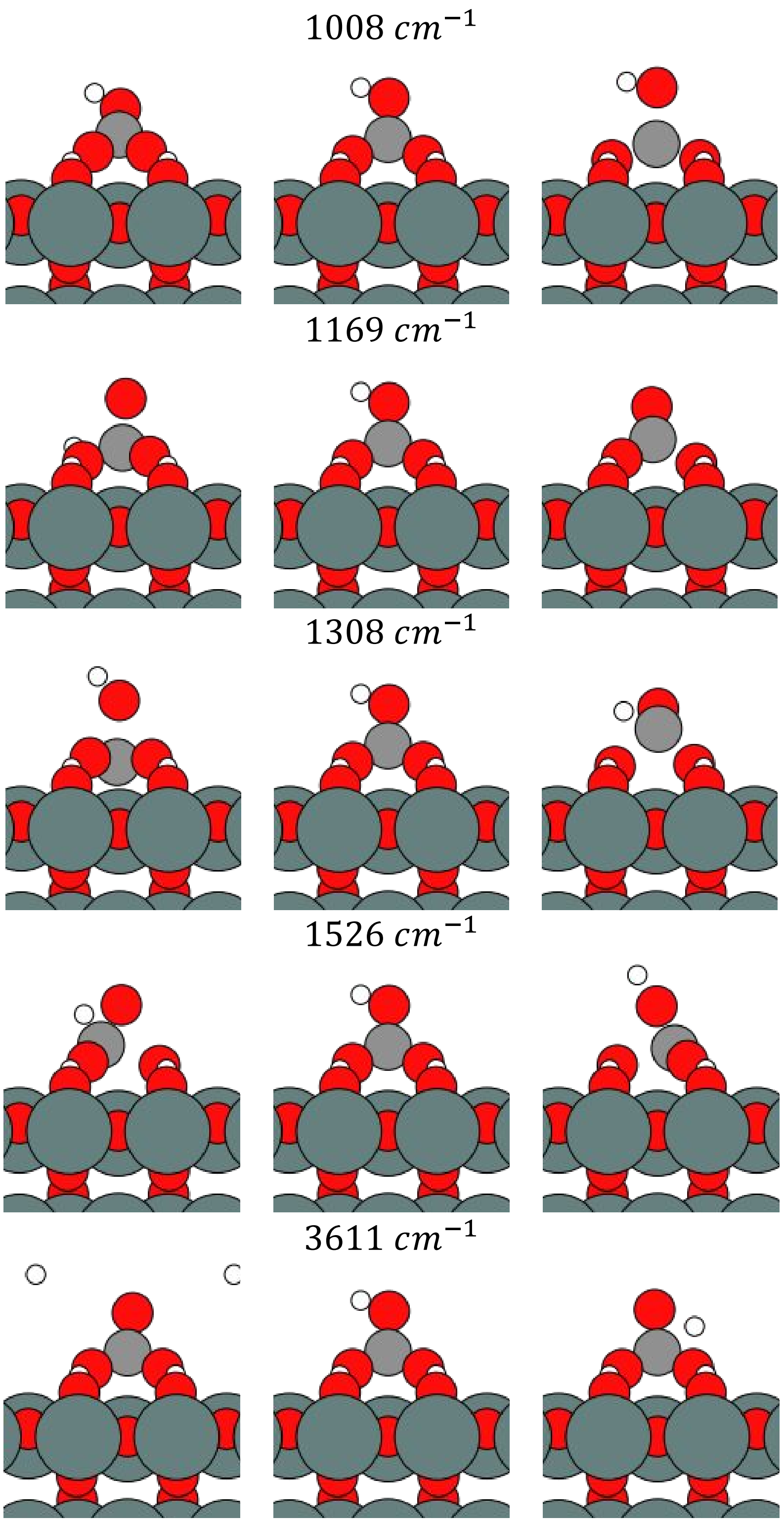

Figure S.28. Visualization of vibrational modes for bidentate HCO_3 on SnO_2 with no cus-water and two cus-vacancies

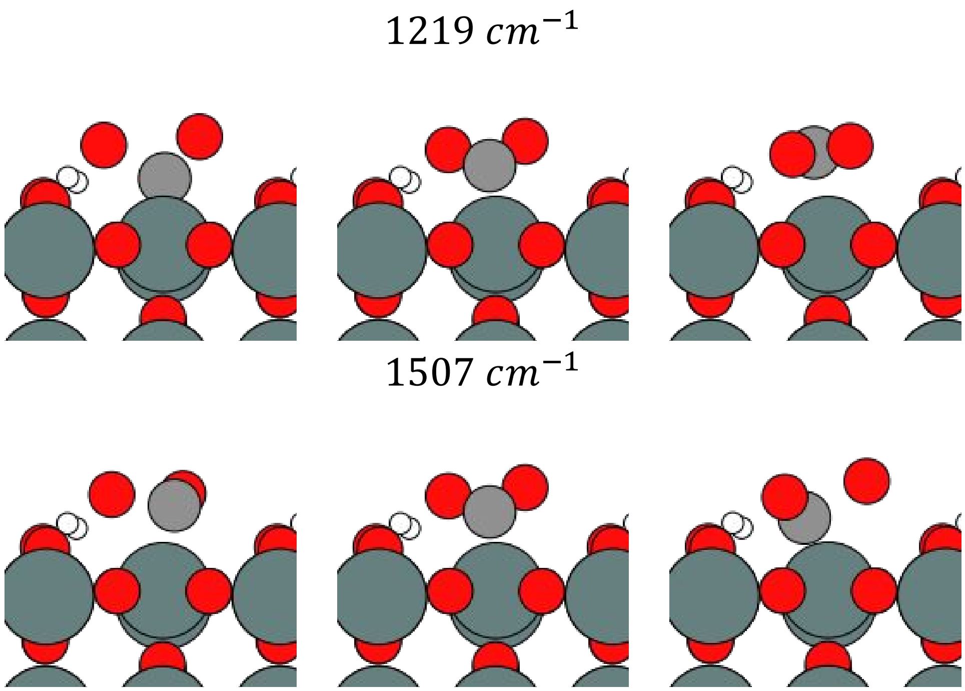

Figure S.29. Visualization of vibrational modes for CO_2 bound through carbon on SnO_2 with no cus-water and two cus-vacancies

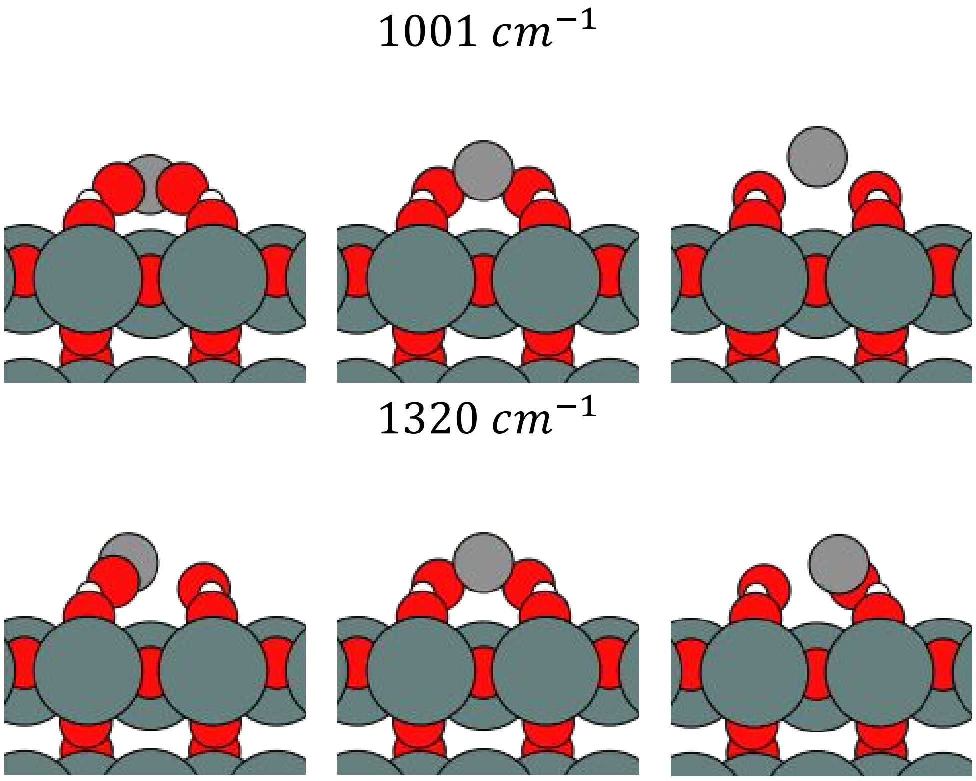

Figure S.30. Visualization of vibrational modes for CO_2 bound through oxygens on SnO_2 with no cus-water and two cus-vacancies

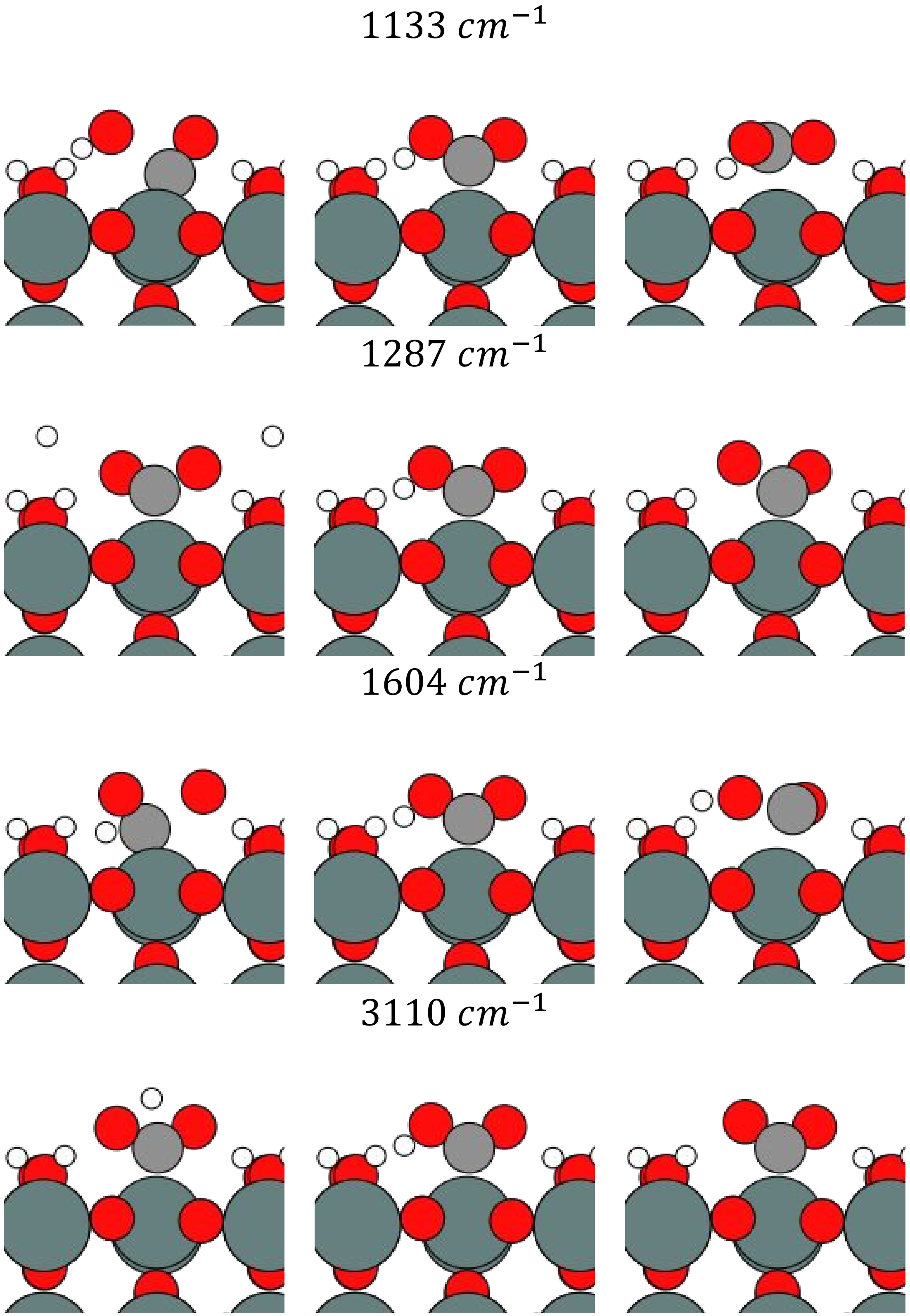

Figure S.31. Visualization of vibrational modes for COOH on SnO_2 with no cus-water and two cus-vacancies

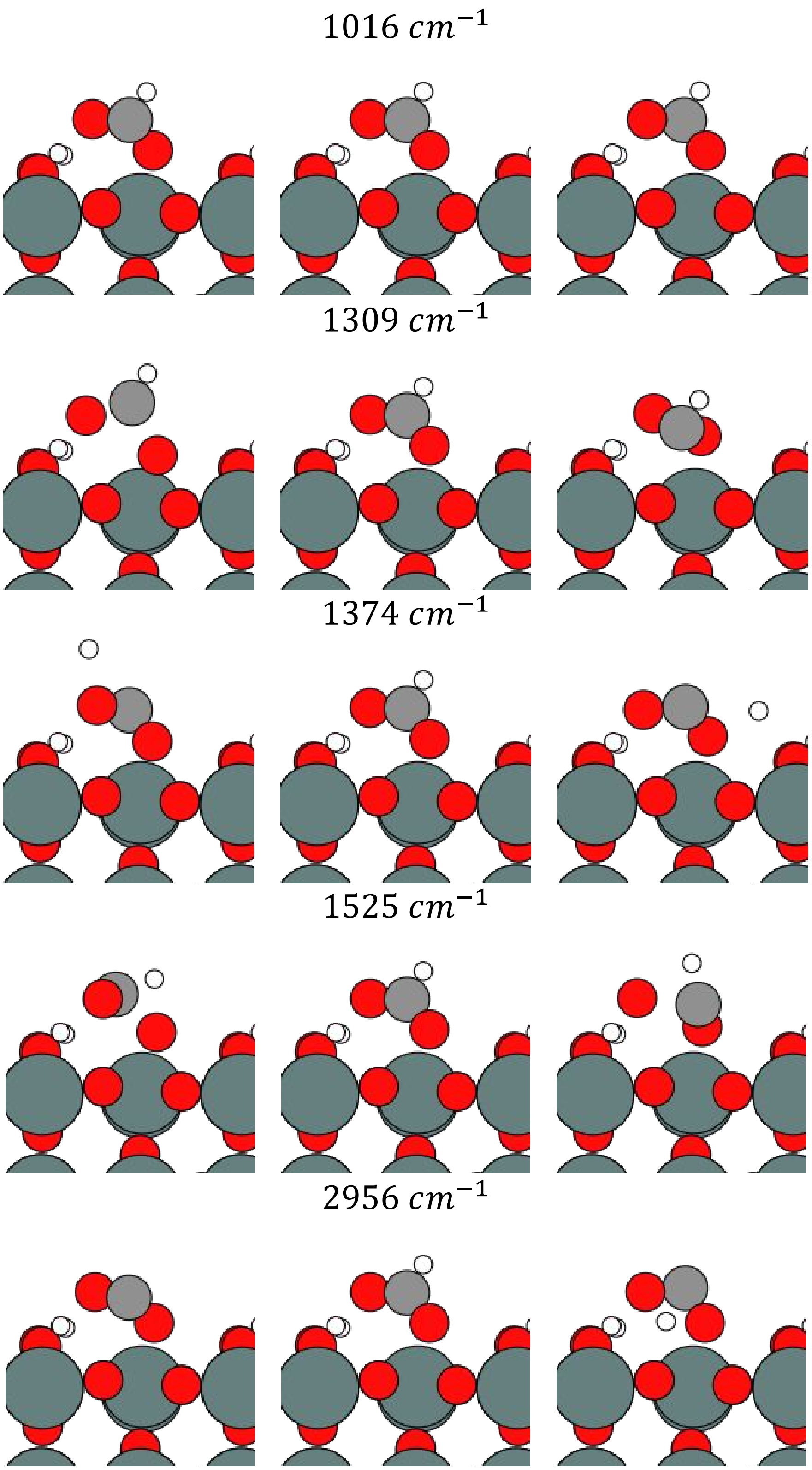

Figure S.32. Visualization of vibrational modes for monodentate OCHO on SnO_2 with no cus-water and two cus-vacancies

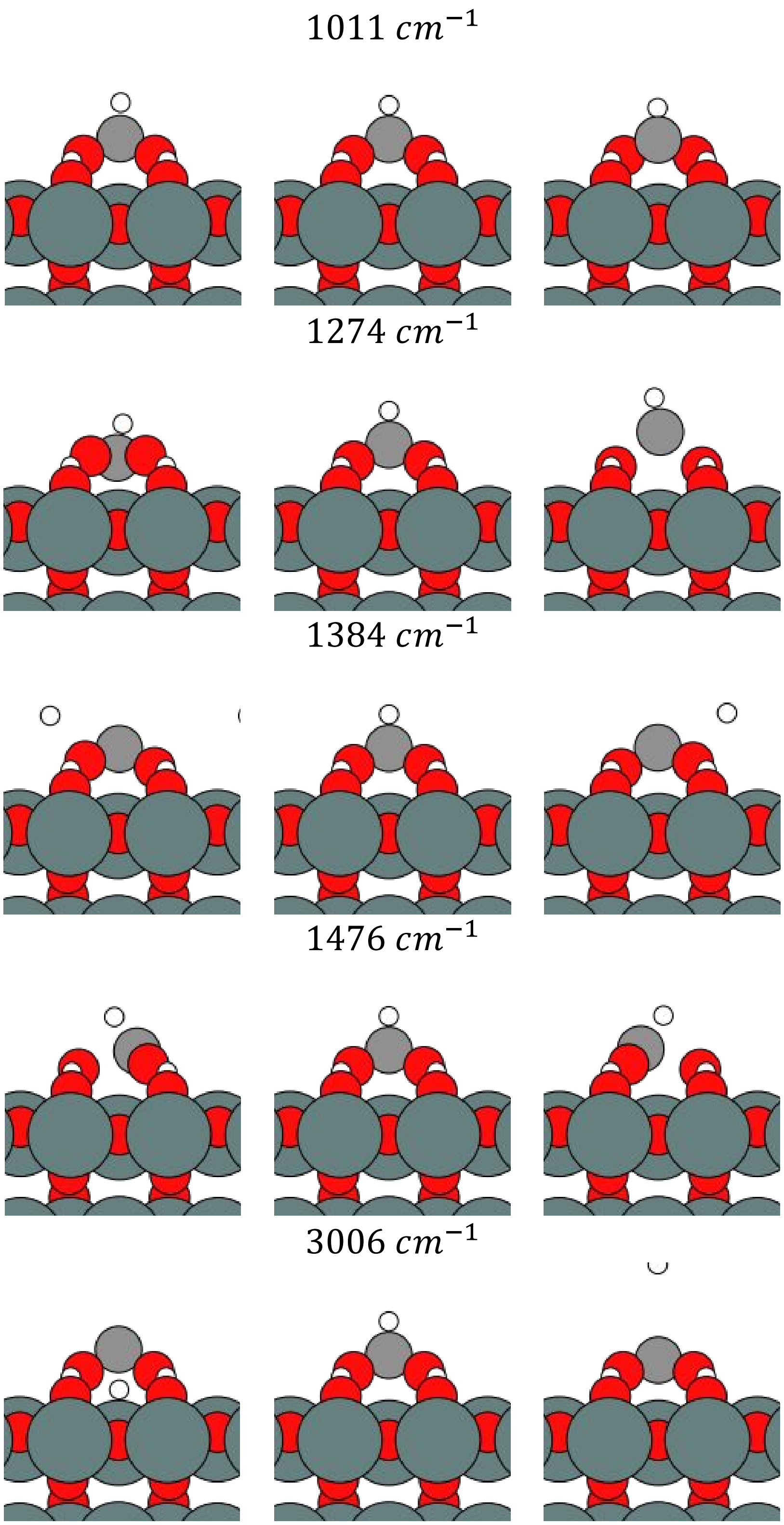

Figure S.33. Visualization of vibrational modes for bidentate OCHO on SnO_2 with no cus-water and two cus-vacancies

Table S.1. Calculated vibrational frequencies for all modes above 900 cm^{-1} for all adsorbates (units are cm^{-1}).

Surface	Adsorbate	0 V_{RHE}	-0.5 V_{RHE}	-1 V_{RHE}
Metallic Sn	CO_3 (mono)	1002	1047	1030
		1199	1230	1215
		1430	1421	1351
	CO_3 (bi)	999	1013	1010
		1232	1182	1233
		1464	1379	1348
	HCO_3 (mono)	975	954	877
		1164	1157	1113
		1269	1236	1257
		1531	1549	1575
		3714	3675	3773
	HCO_3 (bi)	1018	987	925
		1143	1190	1204
		1311	1260	1261
		1530	1550	1541
		3631	3655	3676
	CO_2 (carbon)	1098	1151	1172
		1489	1461	1398
	CO_2 (oxygens)	1230	1094	1028
		2192	1816	1282
	COOH	1012	996	965
		1180	1228	1158
		1633	1585	1577
		3543	3592	3411
	OCHO (mono)	1017	978	994
		1274	1295	1297
		1343	1341	1415
		1502	1492	1482
		2900	2859	2927
	OCHO (bi)	988	967	1110
		1265	1293	1267
		1334	1311	1308
		1513	1478	1443
		2963	2956	2831
SnO_2 – 1 cus-water	CO_3 (mono)	994	1002	1010
		1272	1266	1277
		1478	1437	1430
	HCO_3 (mono)	1013	1006	985
		1136	1147	1105
		1339	1326	1325
		1580	1568	1562

		3647	3652	3665
	CO_2 (carbon)	1249	1246	1278
		1487	1461	1442
	CO_2 (oxygens)	981	987	1038
		1474	1372	1329
	COOH	1137	1128	1118
		1283	1292	1318
		1623	1611	1593
		3192	3092	2963
	OCHO (mono)	1032	1019	1010
		1268	1272	1278
		1385	1376	1371
		1589	1566	1541
		2967	2958	2931
SnO_2 – 0 cus-water	CO_3 (mono)	1037	1036	1030
		1277	1274	1280
		1401	1367	1345
	CO_3 (bi)	993	1000	983
		1200	1201	1191
		1482	1484	1456
	HCO_3 (mono)	1009	994	995
		1070	1058	1053
		1282	1271	1313
		1613	1556	1606
		3060	3066	3026
	HCO_3 (bi)	1025	1008	1000
		1166	1169	1158
		1350	1308	1290
		1517	1526	1515
		3591	3611	3622
	CO_2 (carbon)	1202	1219	1228
		1547	1507	1475
	CO_2 (oxygens)	1000	1001	1040
		1422	1320	1233
	COOH	1146	1133	1109
		1289	1287	1289
		1611	1604	1598
		3178	3110	3034
	OCHO (mono)	1024	1016	1017
		1273	1309	1318
		1372	1374	1378
		1539	1525	1519
		2965	2956	2948
	OCHO (bi)	1009	1011	1003
		1282	1274	1278

	1392	1384	1387
	1480	1476	1454
	3014	3006	2959

Additional CO_2R SEIRAS information

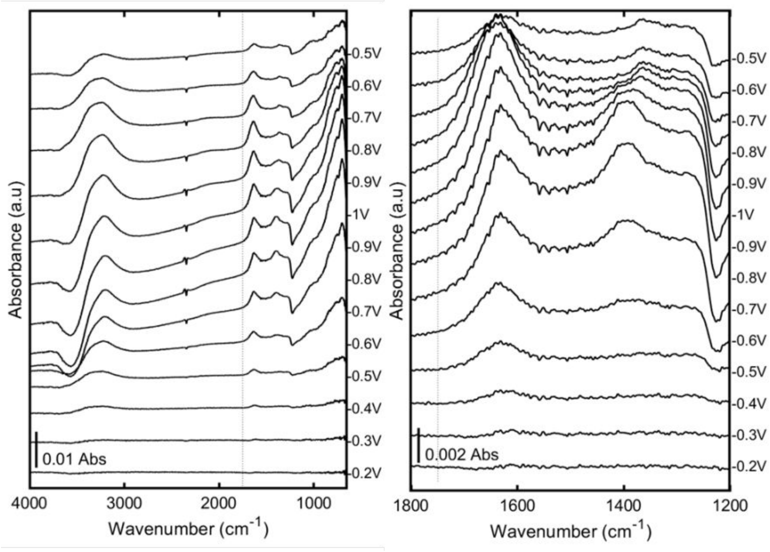

Figure S.34. Full (4000-800 cm^{-1}, left) and carbonaceous region (1800-1200 cm^{-1}, right) ATR-SEIRAS spectra on the metallic Sn electrode during CO_2R.

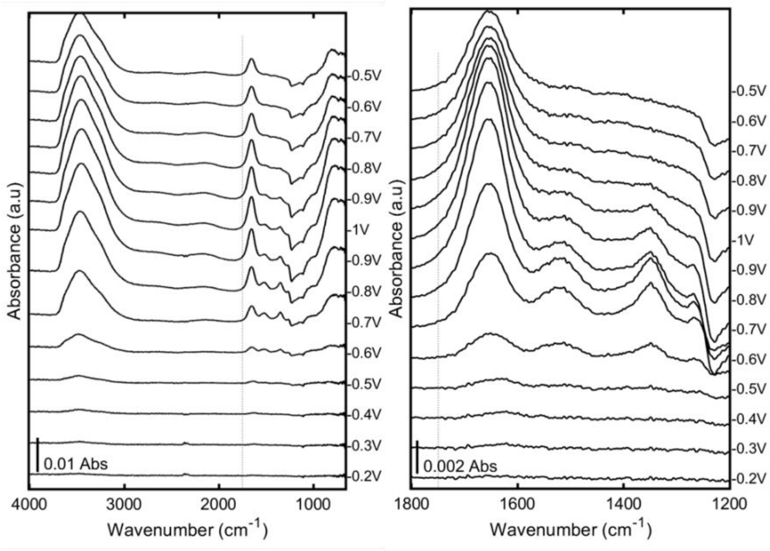

Figure S.35. Full (4000-800 cm^{-1}, left) and carbonaceous region (1800-1200 cm^{-1}, right) ATR-SEIRAS spectra on the oxidized Sn electrode during CO_2R.

Figure S.36 shows the cyclic voltammograms collected during the ATR-SEIRAS experiments above. The raw current and current densities are shown to compare the relative current pull and activity between the two different electrodes. The current drawn from the CO_2R experiment is greater than the Ar-sparged experiment on both materials, but the oxidized Sn has a

larger increase in current upon introduction of CO_2 relative to the metallic Sn. This is consistent with oxidized Sn's more facile activation of CO_2 than metallic Sn. We refrain from further interpretation of the electrochemical data due to the lack of quantification of products, which was outside the scope of the current study.

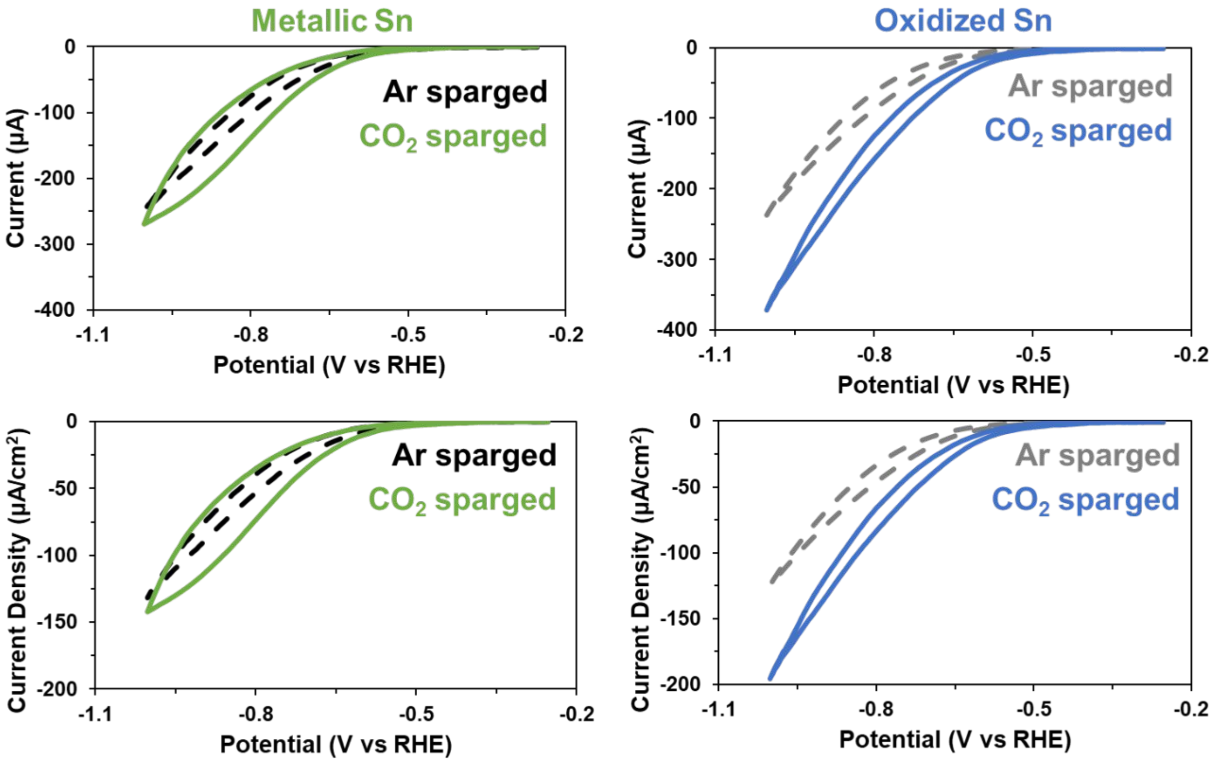

Figure S.36. Cyclic voltammograms, showing current (top row) and current density (bottom row), for metallic Sn (left column) and oxidized Sn (right column) corresponding to CO_2R (solid colored lines) and Ar-sparged (broken greyscale lines) ATR-SEIRAS experiments from **Figure 6** and **Figure S.38**.

Evidence for interfacial buffering by $SnO_2(110)$

$$H_2O^* + CO_3^{2-} \rightarrow OH^* + HCO_3^-$$

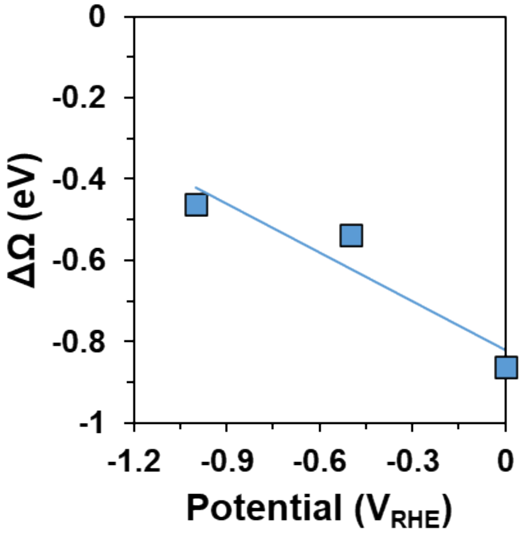

Figure S.37. Change in grand free energy for the transfer of a proton from the doubly protonated $SnO_2(110)$ surface to a free carbonate ion to form an adsorbed hydroxyl and a free bicarbonate ion as a function of potential.

SEIRAS in the absence of CO_2

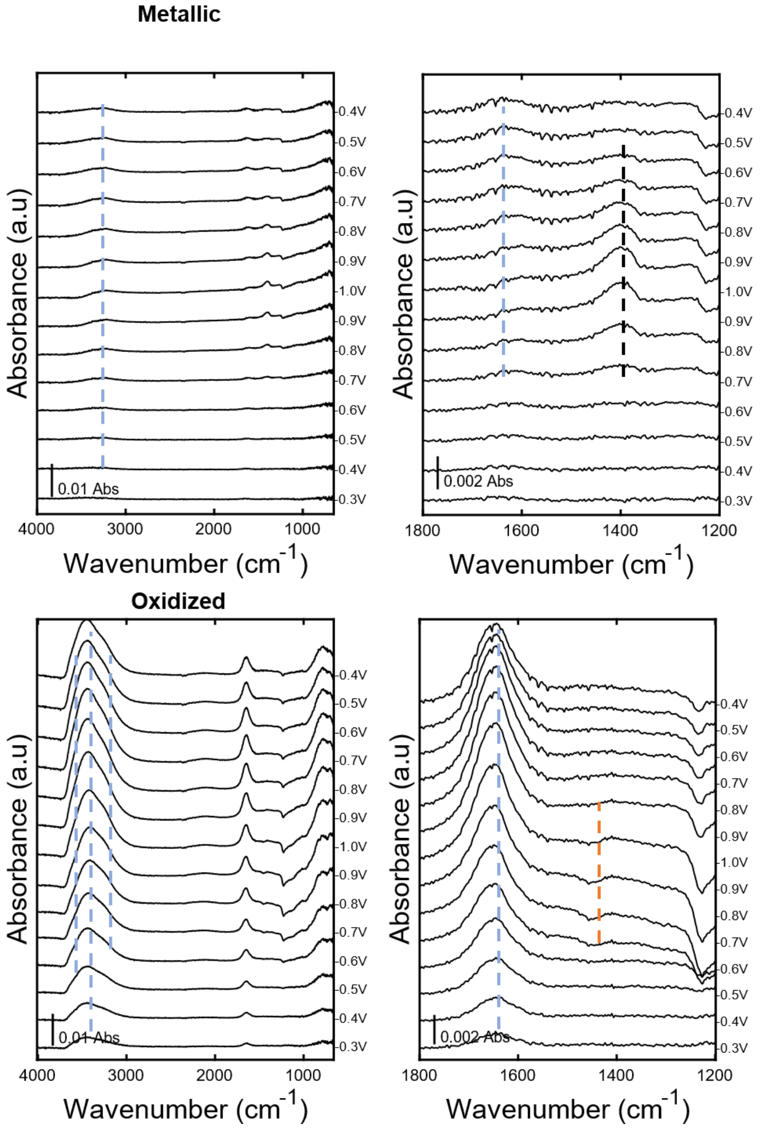

Figure S.38. Full (4000-800 cm^{-1}, top left) and carbonaceous region (1800-1200 cm^{-1}, top right) ATR-SEIRAS spectra on the metallic Sn electrode with no CO_2 present (sparged with Ar). Full (4000-800 cm^{-1}, bottom left) and carbonaceous region (1800-1200 cm^{-1}, bottom right) ATR-SEIRAS spectra on the oxidized Sn electrode electrode with no CO_2 present (sparged with Ar). Colored lines are drawn to guide the eye (spectra without these lines are available in **Figures S.39** and **S.40**).

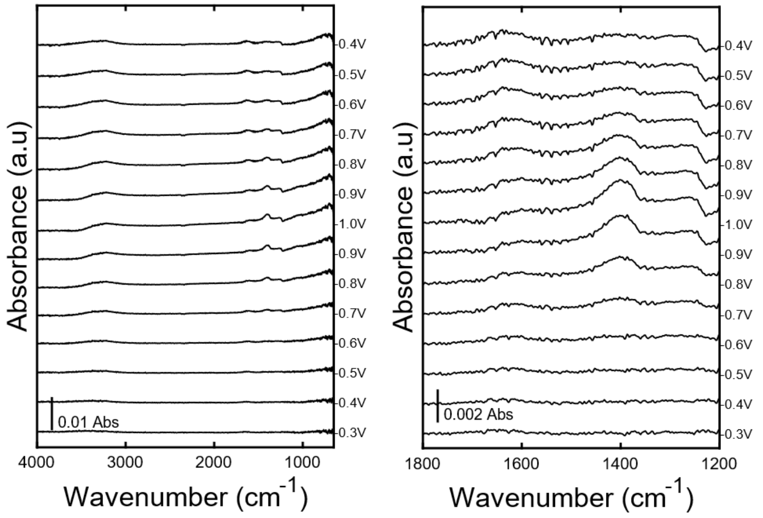

Figure S.39. Full (4000-800 cm^{-1}, left) and carbonaceous region (1800-1200 cm^{-1}, right) ATR-SEIRAS spectra on the metallic Sn electrode with no CO_2 present (sparged with Ar).

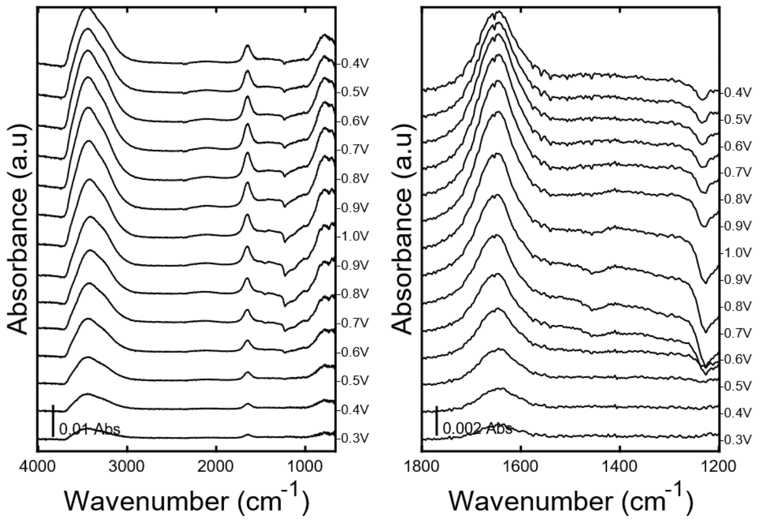

Figure S.40. Full (4000-800 cm^{-1}, left) and carbonaceous region (1800-1200 cm^{-1}, right) ATR-SEIRAS spectra on the oxidized Sn electrode with no CO_2 present (sparged with Ar).

Hydroxyl-mediated CO_2R on SnO_2

We considered the formation of bicarbonate through the nucleophilic attack of CO_2 by br-hydroxyl, which has been suggested as a potential mechanism on oxidized Sn electrodes.[1] We do not consider the hydroxyl mediated mechanism on the metallic Sn surface or at the cus site of the SnO_2 surfaces because the coverage of hydroxyls is expected to be very low in the potential range of CO_2R. In addition to the two SnO_2 surfaces considered for the other CO_2R intermediates ($SnO_2(110)$ with 1 and 0 cus-water groups), we also considered the surface with 2 cus-water groups because the hydroxyl-mediated mechanism doesn't require naked Sn sites. The intermediates are shown in **Figure S.41**.

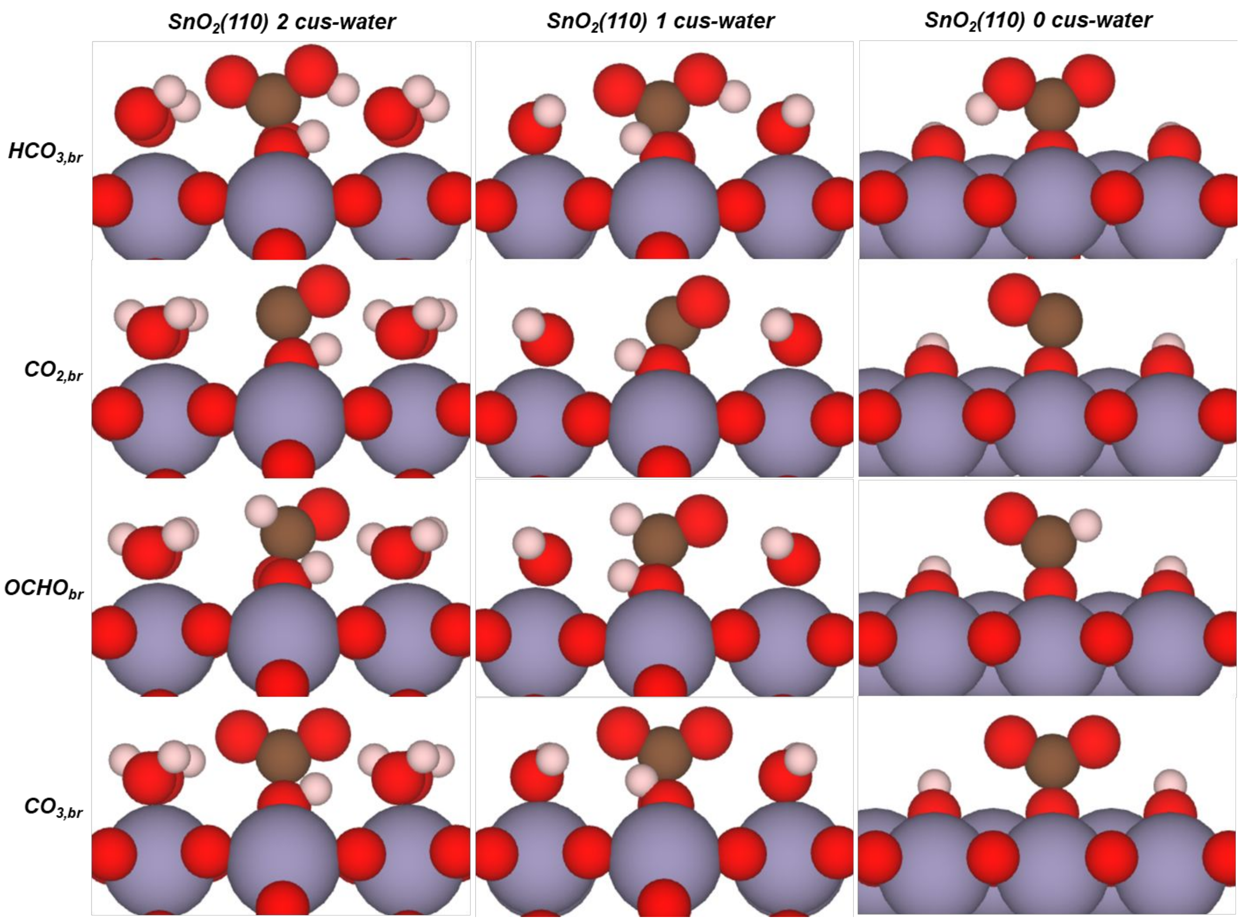

Figure S.41. Converged geometries of the br-hydroxyl-mediated CO_2R intermediates considered at -0.5 V_{RHE} across the three SnO_2 surface.

Before discussing the energetics of the hydroxyl-mediated pathway, it is worth noting that the $CO_{2,br}$ intermediate spontaneously dissociates to CO and O_{br} during geometry optimization at potentials less than -1 V_{RHE}. This is shown in **Figure S.42**. To determine the energetics of this intermediate to evaluate the hydroxyl-mediated pathway, we ran single-point calculations at the lower potentials.

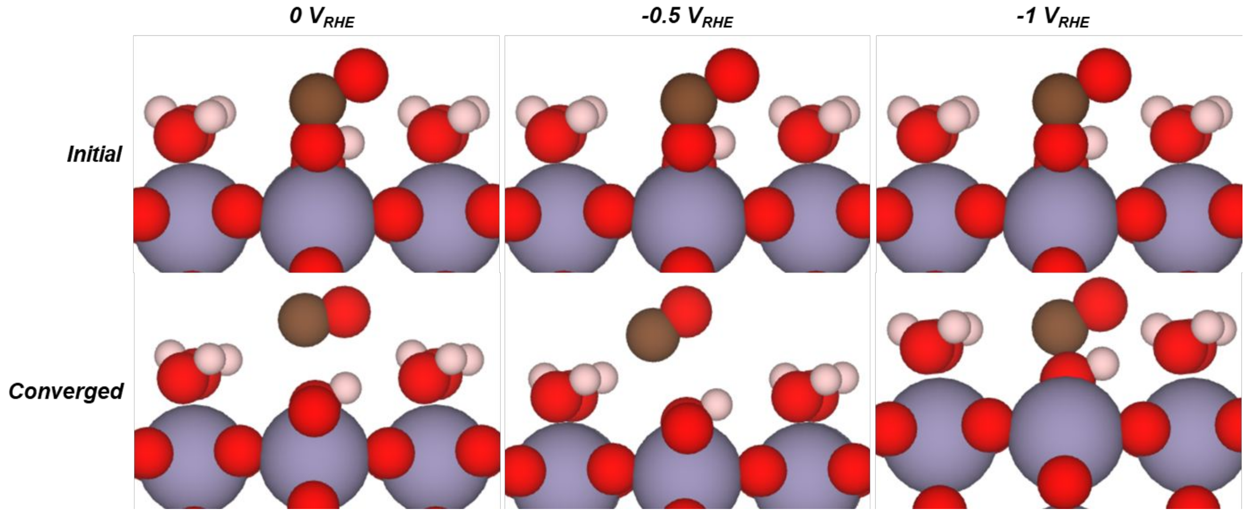

Figure S.42. GC-DFT adsorption geometries for $CO_{2,br}$ on SnO_2(110) with 2 cus-water groups. Top row is the initial guess for each calculation, second row is the converged structure. The other SnO_2 surfaces with 1 and 0 cus-water groups showed the same behavior.

The energetics of the hydroxyl-mediated pathway are shown in **Figure S.43**. The first step of the hydroxyl-mediated pathway is adsorption of CO_2 to form bicarbonate at the br-hydroxyl site. While this step is not formally electrochemical, the GC-DFT calculation predicts a partial charge transfer of ~0.5 electrons. As such, this step becomes more favorable at more negative potentials, but is still mildly uphill even at -1 V_{RHE}. Once bicarbonate is formed, we considered the reduction to $CO_{2,br}$ and H_2O via PCET as the next elementary step. This step is highly endothermic at low potential and becomes favorable at -1 V_{RHE}. This step has a much higher degree of charge transfer (~1.5 electrons) than would've been expected. We also considered the deprotonation of the bridge-bound bicarbonate to form adsorbed carbonate, which would be considered a parasitic reaction (that is, not yielding CO_2R products). This is a formal oxidation (calculated charge transfer of ~0.7 electrons), and as such becomes less favorable at more negative potentials. The deprotonation of bicarbonate is competitive with the reduction to $CO_{2,br}$ at -1 V_{RHE} on the SnO_2 surface with 2 cus-water groups, whereas it is much less favorable when cus-water groups are removed. This indicates that these groups may have lateral interactions that stabilize the carbonate adsorbate. We expect the coverage of these groups to decrease at more negative potentials, so we do not expect the flux of CO_2 to $CO_{3,br}$ to be large under CO_2R operating potentials, and because CO_2 adsorption to $HCO_{3,br}$ is unfavorable at low potential, the surface that would lead to $CO_{3,br}$ is unlikely to form $HCO_{3,br}$ in appreciable quantities.

Once $CO_{2,br}$ is formed, it can either be further reduced to form $OCHO_{br}$ or disproportionate to make CO and O_{br}. Formation of $OCHO_{br}$ is more favorable than CO and O_{br}, and because this is a ~0.5 electron reduction step and forming CO and O_{br} is a ~0.3 electron oxidation step, the gap in favorability grows at more negative potentials. However, because the formation of $CO_{2,br}$ isn't favorable until ~ -1 V_{RHE}, this pathway is only expected to form $OCHO_{br}$ (unless there is a much higher kinetic barrier for this step than the formation of CO). Once $OCHO_{br}$ is formed, the reductive desorption to release formate is the next step. Once again, this step is forced downhill at

more negative potentials due to the transfer of ~1 electron upon desorption. Formate is bound slightly less strongly at the br-site when compared to the cus-sites in the main text, but the desorption only becomes favorable at reasonably negative potentials.

Once either formate or CO has been released, to close the catalytic cycle the br-hydroxyl must be regenerated. In the case of the formate pathway, this must be from the oxidative adsorption of hydroxyl in the solution. It is easily seen that this step must be an oxidation because the sum of the electrons transferred to form formate prior to this step is 2.9-3.2, and only two electrons are required to reduce CO_2 to formate. Indeed, GC-DFT predicts this step to be a ~1 electron oxidation, but because oxygen vacancies at the br-site are highly unstable, this step is still thermodynamically downhill even at -1 V_{RHE}. In the case of the CO pathway, O_{br} must be reduced back to br-hydroxyl via PCET. Interestingly, this step is less favorable on the SnO_2 surface with 2 cus-water groups than on the other two surfaces considered. However, as this is less likely to be the operant surface under the CO_2R potentials, and the reduction of O_{br} is favorable at all potentials on the other surfaces, we do not expect this step to be limiting in the hydroxyl-mediated pathway.

While this pathway is perfectly feasible and may even contribute to the overall CO_2R rate at large negative potentials, we considered it to be a minority pathway. This conclusion is mainly derived from the fact that the steps that lead to formate (or CO) only become favorable at potentials approaching -1 V_{RHE}, while experimentally we, and many others, have shown that Sn based catalysts form CO_2R products at potentials as low as ~ -0.5 V_{RHE}. This analysis also does not include the presence of any potentially large kinetic barriers, which would only increase the overpotential required for measurable rates. Another reason why this mechanism is not expected to majorly contribute to the rate is because there is a narrow potential window where the CO_2R steps and the site regeneration (oxidative re-adsorption of br-hydroxyl) are simultaneously favorable. That is, if the potential is pushed too negative, it is expected that the coverage of br-hydroxyl would decrease and regeneration of this site would be rate limiting. Lastly, our SEIRAS experiments indicate that a stable formate adsorbate is measured in the CO_2R potential window on the oxidized, but not the metallic, Sn surface. The GC-DFT predicted binding energy of formate at the br-site is not quite as weak as on the metallic Sn(200) surface, but it is much weaker than on the cus-site. The presence of a peak assigned to formate in the SEIRAS spectra indicates that the rate of desorption of formate is slow enough to measure a steady coverage. Therefore, because the hydroxyl-mediated pathway forms formate at the br-site, which desorbs more readily than at the cus-site, we believe this pathway may only have a minority contribution to the rate. To authoritatively rule this out, more detailed kinetic measurements are required.

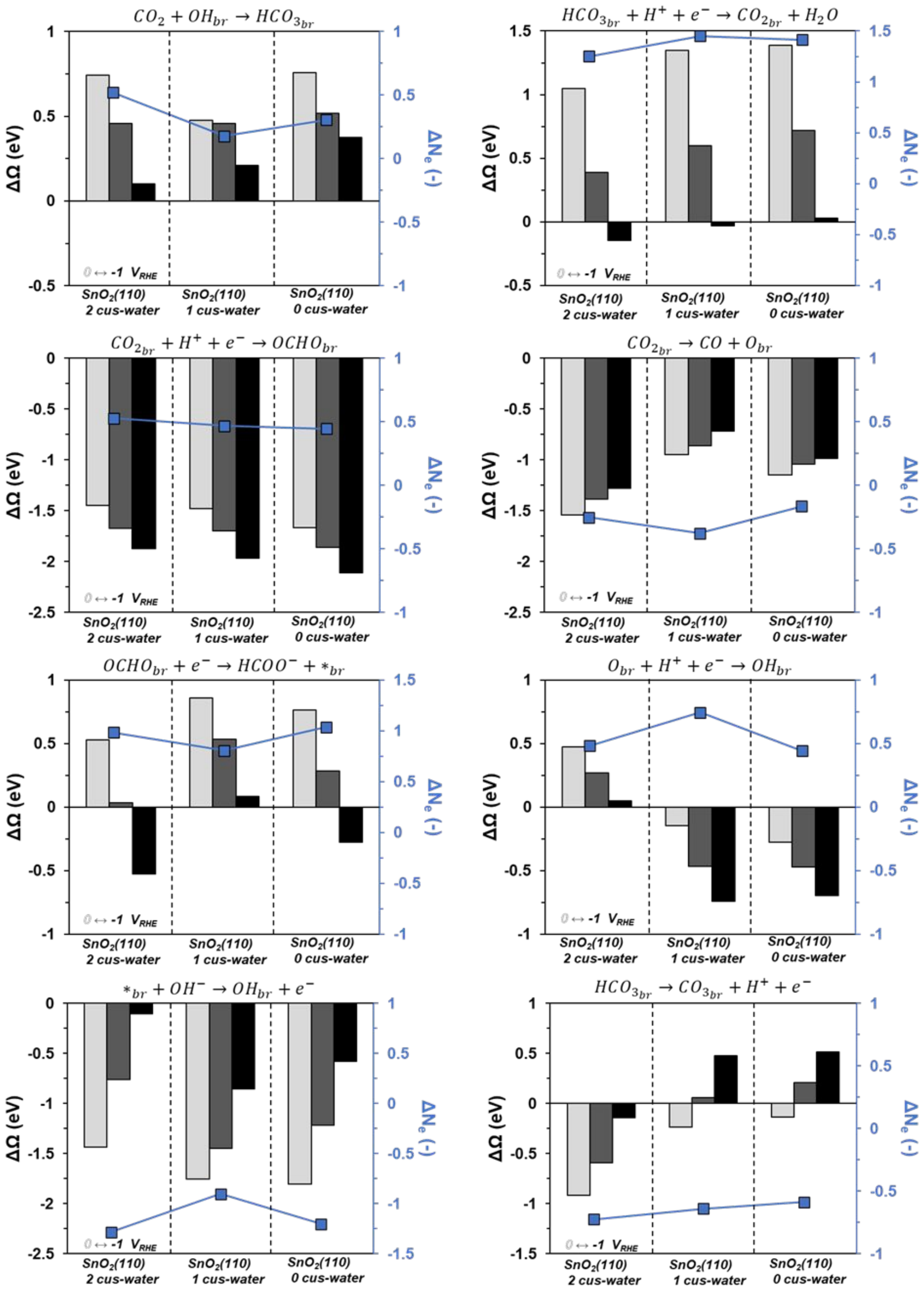

Figure S.43. Change in grand free energy as a function of potential for CO_2R elementary steps on br-hydroxyl sites. The potentials are 0 (light grey), -0.5 (dark grey) and -1 (black) V_{RHE}. The reactions represented are formation of adsorbed bicarbonate via nucleophilic attack of CO_2 by br-hydroxyl (top left), reduction of bicarbonate to adsorbed CO_2 via PCET (top right), formation of adsorbed formate by PCET (upper middle left), formation of CO and br-O (upper middle right), desorption of formate (lower bottom left) re-formation of br-hydroxyl by PCET (lower bottom

right), re-formation of br-hydroxyl by adsorption of free hydroxyl (bottom left) and deprotonation of adsorbed bicarbonate to form adsorbed carbonate (bottom right). The average number of electrons transferred across the three potentials is also shown (in blue).

References

(1) Baruch, M. F.; Pander, J. E.; White, J. L.; Bocarsly, A. B. Mechanistic Insights into the Reduction of CO2 on Tin Electrodes Using in Situ ATR-IR Spectroscopy. *ACS Catal.* **2015**, *5* (5), 3148–3156. https://doi.org/10.1021/acscatal.5b00402.

(2) Holewinski, A.; Linic, S. Elementary Mechanisms in Electrocatalysis: Revisiting the ORR Tafel Slope. *J. Electrochem. Soc.* **2012**, *159* (11), H864. https://doi.org/10.1149/2.022211jes.

(3) He, M.; Xu, B.; Lu, Q. Probing the Role of Surface Speciation of Tin Oxide and Tin Catalysts on CO2 Electroreduction Combining in Situ Raman Spectroscopy and Reactivity Investigations. *Chin. J. Catal.* **2022**, *43* (6), 1473–1477. https://doi.org/10.1016/S1872-2067(21)64014-7.
