# Supplementary figures and images for: Insights into Electrochemical CO2 Reduction on Metallic and Oxidized Tin Using Grand-Canonical DFT and In Situ ATR-SEIRA Spectroscopy (part 2 of 2)
Source: ACS Catal. 2024 May 14;14(11):8353–65. doi: 10.1021/acscatal.4c01290 (PMC11165454; doi:10.1021/acscatal.4c01290)

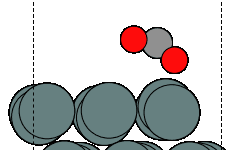

Supplement: Supplementary file 2 — cs4c01290_si_002.zip [file cs4c01290_si_002.zip › vibration animations/Sn(200)/OCHO/monodentate/-1 V_RHE/2927cm-1.gif]

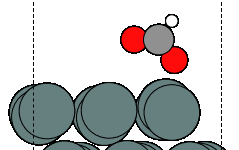

Supplement: Supplementary file 2 — cs4c01290_si_002.zip [file cs4c01290_si_002.zip › vibration animations/Sn(200)/OCHO/monodentate/-1 V_RHE/994cm-1.gif]

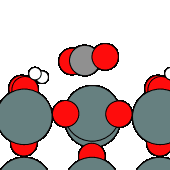

Supplement: Supplementary file 2 — cs4c01290_si_002.zip [file cs4c01290_si_002.zip › vibration animations/SnO2(110)-0cus-water/CO2/bound through carbon/0 V_RHE/1202cm-1.gif]

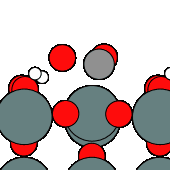

Supplement: Supplementary file 2 — cs4c01290_si_002.zip [file cs4c01290_si_002.zip › vibration animations/SnO2(110)-0cus-water/CO2/bound through carbon/0 V_RHE/1547cm-1.gif]

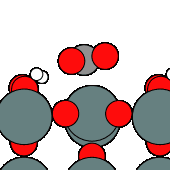

Supplement: Supplementary file 2 — cs4c01290_si_002.zip [file cs4c01290_si_002.zip › vibration animations/SnO2(110)-0cus-water/CO2/bound through carbon/-0.5 V_RHE/1219cm-1.gif]

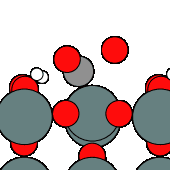

Supplement: Supplementary file 2 — cs4c01290_si_002.zip [file cs4c01290_si_002.zip › vibration animations/SnO2(110)-0cus-water/CO2/bound through carbon/-0.5 V_RHE/1507cm-1.gif]

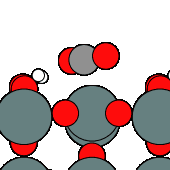

Supplement: Supplementary file 2 — cs4c01290_si_002.zip [file cs4c01290_si_002.zip › vibration animations/SnO2(110)-0cus-water/CO2/bound through carbon/-1 V_RHE/1228cm-1.gif]

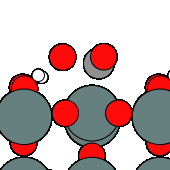

Supplement: Supplementary file 2 — cs4c01290_si_002.zip [file cs4c01290_si_002.zip › vibration animations/SnO2(110)-0cus-water/CO2/bound through carbon/-1 V_RHE/1475cm-1.gif]

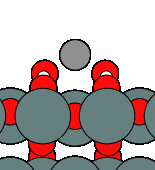

Supplement: Supplementary file 2 — cs4c01290_si_002.zip [file cs4c01290_si_002.zip › vibration animations/SnO2(110)-0cus-water/CO2/bound through oxygen(s)/0 V_RHE/1000cm-1.gif]

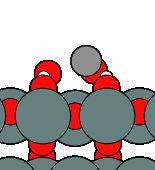

Supplement: Supplementary file 2 — cs4c01290_si_002.zip [file cs4c01290_si_002.zip › vibration animations/SnO2(110)-0cus-water/CO2/bound through oxygen(s)/0 V_RHE/1834cm-1.gif]

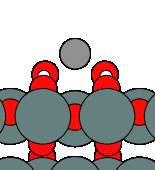

Supplement: Supplementary file 2 — cs4c01290_si_002.zip [file cs4c01290_si_002.zip › vibration animations/SnO2(110)-0cus-water/CO2/bound through oxygen(s)/-0.5 V_RHE/1001cm-1.gif]

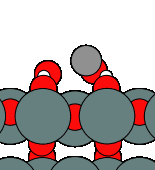

Supplement: Supplementary file 2 — cs4c01290_si_002.zip [file cs4c01290_si_002.zip › vibration animations/SnO2(110)-0cus-water/CO2/bound through oxygen(s)/-0.5 V_RHE/1422cm-1.gif]

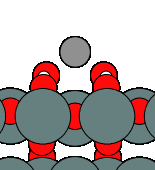

Supplement: Supplementary file 2 — cs4c01290_si_002.zip [file cs4c01290_si_002.zip › vibration animations/SnO2(110)-0cus-water/CO2/bound through oxygen(s)/-1 V_RHE/1040cm-1.gif]

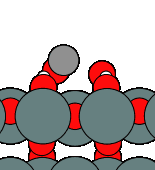

Supplement: Supplementary file 2 — cs4c01290_si_002.zip [file cs4c01290_si_002.zip › vibration animations/SnO2(110)-0cus-water/CO2/bound through oxygen(s)/-1 V_RHE/1233cm-1.gif]

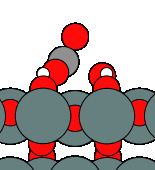

Supplement: Supplementary file 2 — cs4c01290_si_002.zip [file cs4c01290_si_002.zip › vibration animations/SnO2(110)-0cus-water/CO3/bidentate/0 V_RHE/1200cm-1.gif]

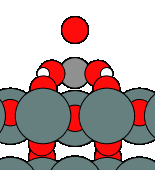

Supplement: Supplementary file 2 — cs4c01290_si_002.zip [file cs4c01290_si_002.zip › vibration animations/SnO2(110)-0cus-water/CO3/bidentate/0 V_RHE/1482cm-1.gif]

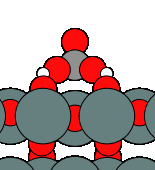

Supplement: Supplementary file 2 — cs4c01290_si_002.zip [file cs4c01290_si_002.zip › vibration animations/SnO2(110)-0cus-water/CO3/bidentate/0 V_RHE/993cm-1.gif]

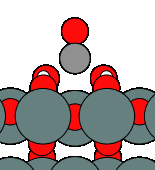

Supplement: Supplementary file 2 — cs4c01290_si_002.zip [file cs4c01290_si_002.zip › vibration animations/SnO2(110)-0cus-water/CO3/bidentate/-0.5 V_RHE/1000cm-1.gif]

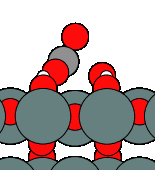

Supplement: Supplementary file 2 — cs4c01290_si_002.zip [file cs4c01290_si_002.zip › vibration animations/SnO2(110)-0cus-water/CO3/bidentate/-0.5 V_RHE/1201cm-1.gif]

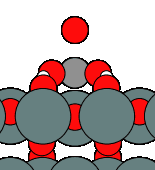

Supplement: Supplementary file 2 — cs4c01290_si_002.zip [file cs4c01290_si_002.zip › vibration animations/SnO2(110)-0cus-water/CO3/bidentate/-0.5 V_RHE/1484cm-1.gif]

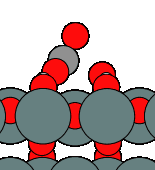

Supplement: Supplementary file 2 — cs4c01290_si_002.zip [file cs4c01290_si_002.zip › vibration animations/SnO2(110)-0cus-water/CO3/bidentate/-1 V_RHE/1191cm-1.gif]

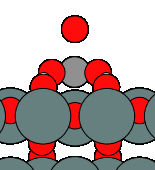

Supplement: Supplementary file 2 — cs4c01290_si_002.zip [file cs4c01290_si_002.zip › vibration animations/SnO2(110)-0cus-water/CO3/bidentate/-1 V_RHE/1456cm-1.gif]

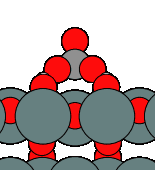

Supplement: Supplementary file 2 — cs4c01290_si_002.zip [file cs4c01290_si_002.zip › vibration animations/SnO2(110)-0cus-water/CO3/bidentate/-1 V_RHE/983cm-1.gif]

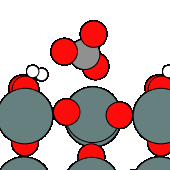

Supplement: Supplementary file 2 — cs4c01290_si_002.zip [file cs4c01290_si_002.zip › vibration animations/SnO2(110)-0cus-water/CO3/monodentate/0 V_RHE/1037cm-1.gif]

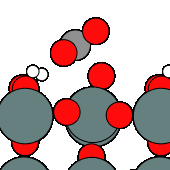

Supplement: Supplementary file 2 — cs4c01290_si_002.zip [file cs4c01290_si_002.zip › vibration animations/SnO2(110)-0cus-water/CO3/monodentate/0 V_RHE/1277cm-1.gif]

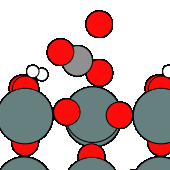

Supplement: Supplementary file 2 — cs4c01290_si_002.zip [file cs4c01290_si_002.zip › vibration animations/SnO2(110)-0cus-water/CO3/monodentate/0 V_RHE/1401cm-1.gif]

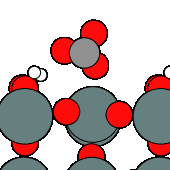

Supplement: Supplementary file 2 — cs4c01290_si_002.zip [file cs4c01290_si_002.zip › vibration animations/SnO2(110)-0cus-water/CO3/monodentate/-0.5 V_RHE/1036cm-1.gif]

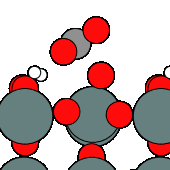

Supplement: Supplementary file 2 — cs4c01290_si_002.zip [file cs4c01290_si_002.zip › vibration animations/SnO2(110)-0cus-water/CO3/monodentate/-0.5 V_RHE/1274cm-1.gif]

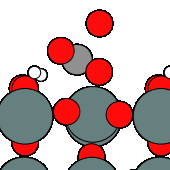

Supplement: Supplementary file 2 — cs4c01290_si_002.zip [file cs4c01290_si_002.zip › vibration animations/SnO2(110)-0cus-water/CO3/monodentate/-0.5 V_RHE/1367cm-1.gif]

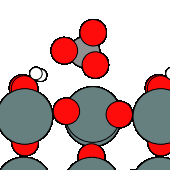

Supplement: Supplementary file 2 — cs4c01290_si_002.zip [file cs4c01290_si_002.zip › vibration animations/SnO2(110)-0cus-water/CO3/monodentate/-1 V_RHE/1030cm-1.gif]

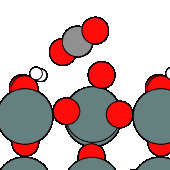

Supplement: Supplementary file 2 — cs4c01290_si_002.zip [file cs4c01290_si_002.zip › vibration animations/SnO2(110)-0cus-water/CO3/monodentate/-1 V_RHE/1280cm-1.gif]

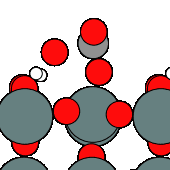

Supplement: Supplementary file 2 — cs4c01290_si_002.zip [file cs4c01290_si_002.zip › vibration animations/SnO2(110)-0cus-water/CO3/monodentate/-1 V_RHE/1345cm-1.gif]

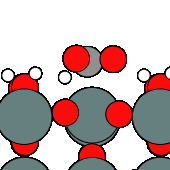

Supplement: Supplementary file 2 — cs4c01290_si_002.zip [file cs4c01290_si_002.zip › vibration animations/SnO2(110)-0cus-water/COOH/0 V_RHE/1146cm-1.gif]

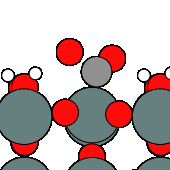

Supplement: Supplementary file 2 — cs4c01290_si_002.zip [file cs4c01290_si_002.zip › vibration animations/SnO2(110)-0cus-water/COOH/0 V_RHE/1289cm-1.gif]

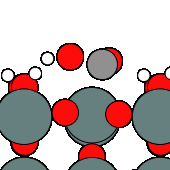

Supplement: Supplementary file 2 — cs4c01290_si_002.zip [file cs4c01290_si_002.zip › vibration animations/SnO2(110)-0cus-water/COOH/0 V_RHE/1611cm-1.gif]

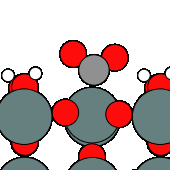

Supplement: Supplementary file 2 — cs4c01290_si_002.zip [file cs4c01290_si_002.zip › vibration animations/SnO2(110)-0cus-water/COOH/0 V_RHE/3178cm-1.gif]

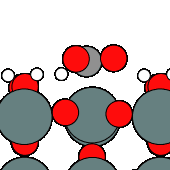

Supplement: Supplementary file 2 — cs4c01290_si_002.zip [file cs4c01290_si_002.zip › vibration animations/SnO2(110)-0cus-water/COOH/-0.5 V_RHE/1133cm-1.gif]

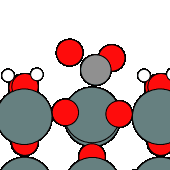

Supplement: Supplementary file 2 — cs4c01290_si_002.zip [file cs4c01290_si_002.zip › vibration animations/SnO2(110)-0cus-water/COOH/-0.5 V_RHE/1287cm-1.gif]

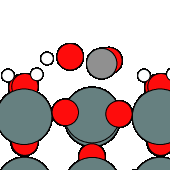

Supplement: Supplementary file 2 — cs4c01290_si_002.zip [file cs4c01290_si_002.zip › vibration animations/SnO2(110)-0cus-water/COOH/-0.5 V_RHE/1604cm-1.gif]

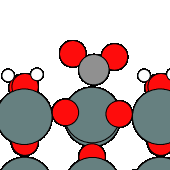

Supplement: Supplementary file 2 — cs4c01290_si_002.zip [file cs4c01290_si_002.zip › vibration animations/SnO2(110)-0cus-water/COOH/-0.5 V_RHE/3110cm-1.gif]

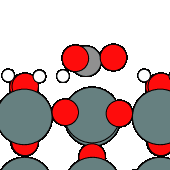

Supplement: Supplementary file 2 — cs4c01290_si_002.zip [file cs4c01290_si_002.zip › vibration animations/SnO2(110)-0cus-water/COOH/-1 V_RHE/1109cm-1.gif]

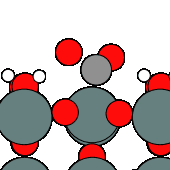

Supplement: Supplementary file 2 — cs4c01290_si_002.zip [file cs4c01290_si_002.zip › vibration animations/SnO2(110)-0cus-water/COOH/-1 V_RHE/1289cm-1.gif]

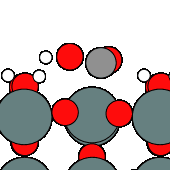

Supplement: Supplementary file 2 — cs4c01290_si_002.zip [file cs4c01290_si_002.zip › vibration animations/SnO2(110)-0cus-water/COOH/-1 V_RHE/1598cm-1.gif]

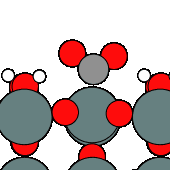

Supplement: Supplementary file 2 — cs4c01290_si_002.zip [file cs4c01290_si_002.zip › vibration animations/SnO2(110)-0cus-water/COOH/-1 V_RHE/3034cm-1.gif]

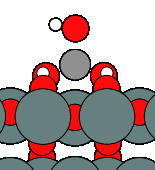

Supplement: Supplementary file 2 — cs4c01290_si_002.zip [file cs4c01290_si_002.zip › vibration animations/SnO2(110)-0cus-water/HCO3/bidentate/0 V_RHE/1025cm-1.gif]

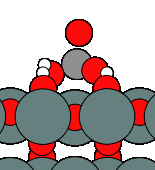

Supplement: Supplementary file 2 — cs4c01290_si_002.zip [file cs4c01290_si_002.zip › vibration animations/SnO2(110)-0cus-water/HCO3/bidentate/0 V_RHE/1166cm-1.gif]

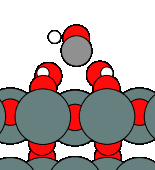

Supplement: Supplementary file 2 — cs4c01290_si_002.zip [file cs4c01290_si_002.zip › vibration animations/SnO2(110)-0cus-water/HCO3/bidentate/0 V_RHE/1350cm-1.gif]

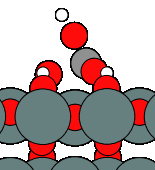

Supplement: Supplementary file 2 — cs4c01290_si_002.zip [file cs4c01290_si_002.zip › vibration animations/SnO2(110)-0cus-water/HCO3/bidentate/0 V_RHE/1517cm-1.gif]

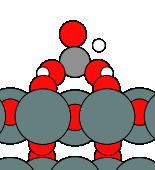

Supplement: Supplementary file 2 — cs4c01290_si_002.zip [file cs4c01290_si_002.zip › vibration animations/SnO2(110)-0cus-water/HCO3/bidentate/0 V_RHE/3591cm-1.gif]

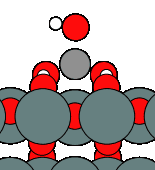

Supplement: Supplementary file 2 — cs4c01290_si_002.zip [file cs4c01290_si_002.zip › vibration animations/SnO2(110)-0cus-water/HCO3/bidentate/-0.5 V_RHE/1008cm-1.gif]

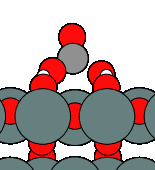

Supplement: Supplementary file 2 — cs4c01290_si_002.zip [file cs4c01290_si_002.zip › vibration animations/SnO2(110)-0cus-water/HCO3/bidentate/-0.5 V_RHE/1169cm-1.gif]

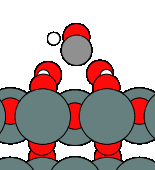

Supplement: Supplementary file 2 — cs4c01290_si_002.zip [file cs4c01290_si_002.zip › vibration animations/SnO2(110)-0cus-water/HCO3/bidentate/-0.5 V_RHE/1308cm-1.gif]

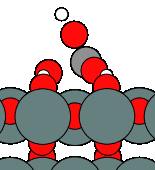

Supplement: Supplementary file 2 — cs4c01290_si_002.zip [file cs4c01290_si_002.zip › vibration animations/SnO2(110)-0cus-water/HCO3/bidentate/-0.5 V_RHE/1526cm-1.gif]

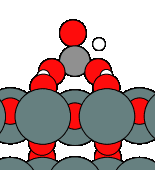

Supplement: Supplementary file 2 — cs4c01290_si_002.zip [file cs4c01290_si_002.zip › vibration animations/SnO2(110)-0cus-water/HCO3/bidentate/-0.5 V_RHE/3611cm-1.gif]

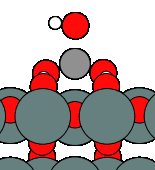

Supplement: Supplementary file 2 — cs4c01290_si_002.zip [file cs4c01290_si_002.zip › vibration animations/SnO2(110)-0cus-water/HCO3/bidentate/-1 V_RHE/1000cm-1.gif]

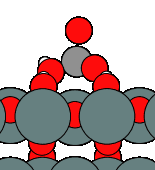

Supplement: Supplementary file 2 — cs4c01290_si_002.zip [file cs4c01290_si_002.zip › vibration animations/SnO2(110)-0cus-water/HCO3/bidentate/-1 V_RHE/1158cm-1.gif]

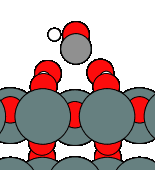

Supplement: Supplementary file 2 — cs4c01290_si_002.zip [file cs4c01290_si_002.zip › vibration animations/SnO2(110)-0cus-water/HCO3/bidentate/-1 V_RHE/1290cm-1.gif]

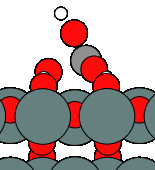

Supplement: Supplementary file 2 — cs4c01290_si_002.zip [file cs4c01290_si_002.zip › vibration animations/SnO2(110)-0cus-water/HCO3/bidentate/-1 V_RHE/1515cm-1.gif]

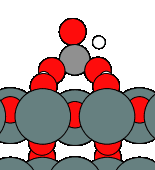

Supplement: Supplementary file 2 — cs4c01290_si_002.zip [file cs4c01290_si_002.zip › vibration animations/SnO2(110)-0cus-water/HCO3/bidentate/-1 V_RHE/3622cm-1.gif]

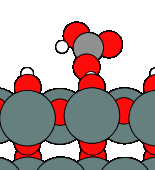

Supplement: Supplementary file 2 — cs4c01290_si_002.zip [file cs4c01290_si_002.zip › vibration animations/SnO2(110)-0cus-water/HCO3/monodentate/0 V_RHE/1009cm-1.gif]

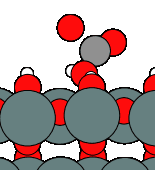

Supplement: Supplementary file 2 — cs4c01290_si_002.zip [file cs4c01290_si_002.zip › vibration animations/SnO2(110)-0cus-water/HCO3/monodentate/0 V_RHE/1070cm-1.gif]

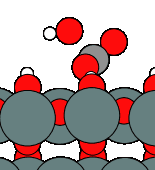

Supplement: Supplementary file 2 — cs4c01290_si_002.zip [file cs4c01290_si_002.zip › vibration animations/SnO2(110)-0cus-water/HCO3/monodentate/0 V_RHE/1282cm-1.gif]

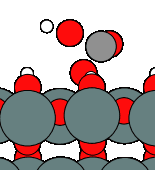

Supplement: Supplementary file 2 — cs4c01290_si_002.zip [file cs4c01290_si_002.zip › vibration animations/SnO2(110)-0cus-water/HCO3/monodentate/0 V_RHE/1613cm-1.gif]

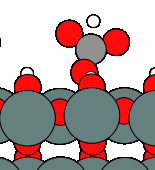

Supplement: Supplementary file 2 — cs4c01290_si_002.zip [file cs4c01290_si_002.zip › vibration animations/SnO2(110)-0cus-water/HCO3/monodentate/0 V_RHE/3060cm-1.gif]

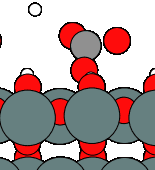

Supplement: Supplementary file 2 — cs4c01290_si_002.zip [file cs4c01290_si_002.zip › vibration animations/SnO2(110)-0cus-water/HCO3/monodentate/-0.5 V_RHE/1058cm-1.gif]

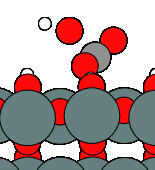

Supplement: Supplementary file 2 — cs4c01290_si_002.zip [file cs4c01290_si_002.zip › vibration animations/SnO2(110)-0cus-water/HCO3/monodentate/-0.5 V_RHE/1271cm-1.gif]

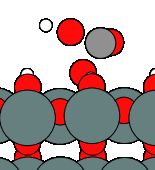

Supplement: Supplementary file 2 — cs4c01290_si_002.zip [file cs4c01290_si_002.zip › vibration animations/SnO2(110)-0cus-water/HCO3/monodentate/-0.5 V_RHE/1556cm-1.gif]

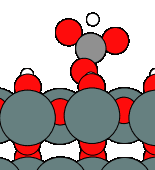

Supplement: Supplementary file 2 — cs4c01290_si_002.zip [file cs4c01290_si_002.zip › vibration animations/SnO2(110)-0cus-water/HCO3/monodentate/-0.5 V_RHE/3066cm-1.gif]

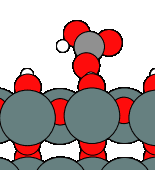

Supplement: Supplementary file 2 — cs4c01290_si_002.zip [file cs4c01290_si_002.zip › vibration animations/SnO2(110)-0cus-water/HCO3/monodentate/-0.5 V_RHE/994cm-1.gif]

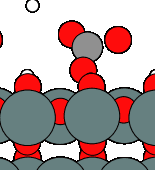

Supplement: Supplementary file 2 — cs4c01290_si_002.zip [file cs4c01290_si_002.zip › vibration animations/SnO2(110)-0cus-water/HCO3/monodentate/-1 V_RHE/1053cm-1.gif]

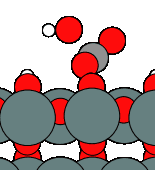

Supplement: Supplementary file 2 — cs4c01290_si_002.zip [file cs4c01290_si_002.zip › vibration animations/SnO2(110)-0cus-water/HCO3/monodentate/-1 V_RHE/1313cm-1.gif]

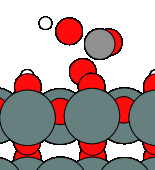

Supplement: Supplementary file 2 — cs4c01290_si_002.zip [file cs4c01290_si_002.zip › vibration animations/SnO2(110)-0cus-water/HCO3/monodentate/-1 V_RHE/1606cm-1.gif]

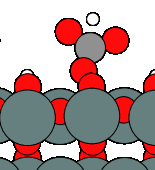

Supplement: Supplementary file 2 — cs4c01290_si_002.zip [file cs4c01290_si_002.zip › vibration animations/SnO2(110)-0cus-water/HCO3/monodentate/-1 V_RHE/3026cm-1.gif]

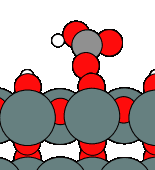

Supplement: Supplementary file 2 — cs4c01290_si_002.zip [file cs4c01290_si_002.zip › vibration animations/SnO2(110)-0cus-water/HCO3/monodentate/-1 V_RHE/995cm-1.gif]

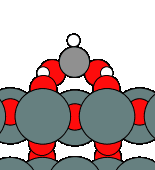

Supplement: Supplementary file 2 — cs4c01290_si_002.zip [file cs4c01290_si_002.zip › vibration animations/SnO2(110)-0cus-water/OCHO/bidentate/0 V_RHE/1009cm-1.gif]

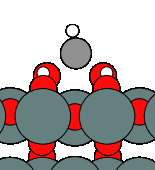

Supplement: Supplementary file 2 — cs4c01290_si_002.zip [file cs4c01290_si_002.zip › vibration animations/SnO2(110)-0cus-water/OCHO/bidentate/0 V_RHE/1282cm-1.gif]

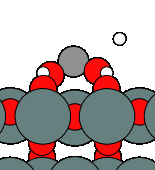

Supplement: Supplementary file 2 — cs4c01290_si_002.zip [file cs4c01290_si_002.zip › vibration animations/SnO2(110)-0cus-water/OCHO/bidentate/0 V_RHE/1392cm-1.gif]

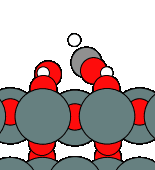

Supplement: Supplementary file 2 — cs4c01290_si_002.zip [file cs4c01290_si_002.zip › vibration animations/SnO2(110)-0cus-water/OCHO/bidentate/0 V_RHE/1480cm-1.gif]

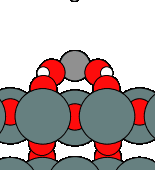

Supplement: Supplementary file 2 — cs4c01290_si_002.zip [file cs4c01290_si_002.zip › vibration animations/SnO2(110)-0cus-water/OCHO/bidentate/0 V_RHE/3014cm-1.gif]

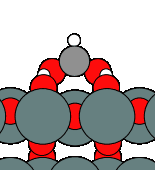

Supplement: Supplementary file 2 — cs4c01290_si_002.zip [file cs4c01290_si_002.zip › vibration animations/SnO2(110)-0cus-water/OCHO/bidentate/-0.5 V_RHE/1011cm-1.gif]

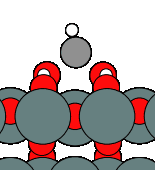

Supplement: Supplementary file 2 — cs4c01290_si_002.zip [file cs4c01290_si_002.zip › vibration animations/SnO2(110)-0cus-water/OCHO/bidentate/-0.5 V_RHE/1274cm-1.gif]

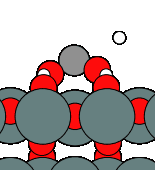

Supplement: Supplementary file 2 — cs4c01290_si_002.zip [file cs4c01290_si_002.zip › vibration animations/SnO2(110)-0cus-water/OCHO/bidentate/-0.5 V_RHE/1384cm-1.gif]

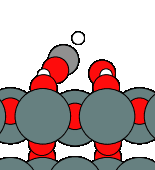

Supplement: Supplementary file 2 — cs4c01290_si_002.zip [file cs4c01290_si_002.zip › vibration animations/SnO2(110)-0cus-water/OCHO/bidentate/-0.5 V_RHE/1476cm-1.gif]

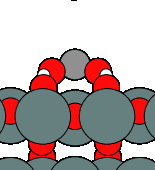

Supplement: Supplementary file 2 — cs4c01290_si_002.zip [file cs4c01290_si_002.zip › vibration animations/SnO2(110)-0cus-water/OCHO/bidentate/-0.5 V_RHE/3006cm-1.gif]

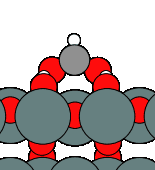

Supplement: Supplementary file 2 — cs4c01290_si_002.zip [file cs4c01290_si_002.zip › vibration animations/SnO2(110)-0cus-water/OCHO/bidentate/-1 V_RHE/1003cm-1.gif]

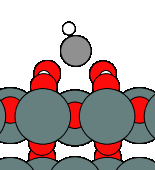

Supplement: Supplementary file 2 — cs4c01290_si_002.zip [file cs4c01290_si_002.zip › vibration animations/SnO2(110)-0cus-water/OCHO/bidentate/-1 V_RHE/1278cm-1.gif]

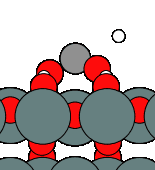

Supplement: Supplementary file 2 — cs4c01290_si_002.zip [file cs4c01290_si_002.zip › vibration animations/SnO2(110)-0cus-water/OCHO/bidentate/-1 V_RHE/1387cm-1.gif]

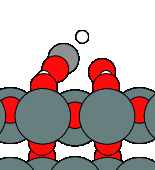

Supplement: Supplementary file 2 — cs4c01290_si_002.zip [file cs4c01290_si_002.zip › vibration animations/SnO2(110)-0cus-water/OCHO/bidentate/-1 V_RHE/1454cm-1.gif]

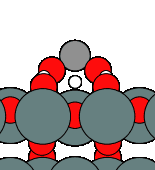

Supplement: Supplementary file 2 — cs4c01290_si_002.zip [file cs4c01290_si_002.zip › vibration animations/SnO2(110)-0cus-water/OCHO/bidentate/-1 V_RHE/2959cm-1.gif]

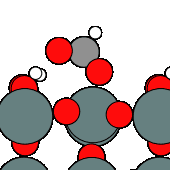

Supplement: Supplementary file 2 — cs4c01290_si_002.zip [file cs4c01290_si_002.zip › vibration animations/SnO2(110)-0cus-water/OCHO/monodentate/0 V_RHE/1024cm-1.gif]

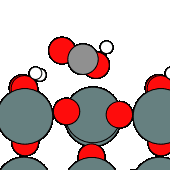

Supplement: Supplementary file 2 — cs4c01290_si_002.zip [file cs4c01290_si_002.zip › vibration animations/SnO2(110)-0cus-water/OCHO/monodentate/0 V_RHE/1273cm-1.gif]

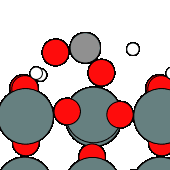

Supplement: Supplementary file 2 — cs4c01290_si_002.zip [file cs4c01290_si_002.zip › vibration animations/SnO2(110)-0cus-water/OCHO/monodentate/0 V_RHE/1372cm-1.gif]

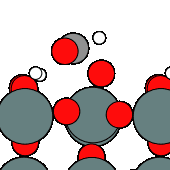

Supplement: Supplementary file 2 — cs4c01290_si_002.zip [file cs4c01290_si_002.zip › vibration animations/SnO2(110)-0cus-water/OCHO/monodentate/0 V_RHE/1539cm-1.gif]

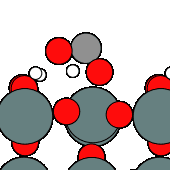

Supplement: Supplementary file 2 — cs4c01290_si_002.zip [file cs4c01290_si_002.zip › vibration animations/SnO2(110)-0cus-water/OCHO/monodentate/0 V_RHE/2965cm-1.gif]

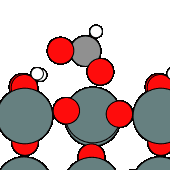

Supplement: Supplementary file 2 — cs4c01290_si_002.zip [file cs4c01290_si_002.zip › vibration animations/SnO2(110)-0cus-water/OCHO/monodentate/-0.5 V_RHE/1016cm-1.gif]

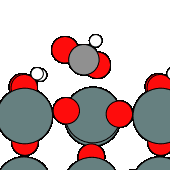

Supplement: Supplementary file 2 — cs4c01290_si_002.zip [file cs4c01290_si_002.zip › vibration animations/SnO2(110)-0cus-water/OCHO/monodentate/-0.5 V_RHE/1309cm-1.gif]

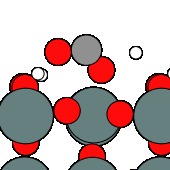

Supplement: Supplementary file 2 — cs4c01290_si_002.zip [file cs4c01290_si_002.zip › vibration animations/SnO2(110)-0cus-water/OCHO/monodentate/-0.5 V_RHE/1374cm-1.gif]

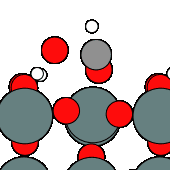

Supplement: Supplementary file 2 — cs4c01290_si_002.zip [file cs4c01290_si_002.zip › vibration animations/SnO2(110)-0cus-water/OCHO/monodentate/-0.5 V_RHE/1525cm-1.gif]

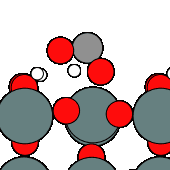

Supplement: Supplementary file 2 — cs4c01290_si_002.zip [file cs4c01290_si_002.zip › vibration animations/SnO2(110)-0cus-water/OCHO/monodentate/-0.5 V_RHE/2956cm-1.gif]

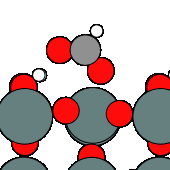

Supplement: Supplementary file 2 — cs4c01290_si_002.zip [file cs4c01290_si_002.zip › vibration animations/SnO2(110)-0cus-water/OCHO/monodentate/-1 V_RHE/1017cm-1.gif]
