# Supplementary figures and images for: Insights into Electrochemical CO2 Reduction on Metallic and Oxidized Tin Using Grand-Canonical DFT and In Situ ATR-SEIRA Spectroscopy (part 1 of 2)
Source: ACS Catal. 2024 May 14;14(11):8353–65. doi: 10.1021/acscatal.4c01290 (PMC11165454; doi:10.1021/acscatal.4c01290)

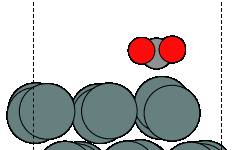

Supplement: Supplementary file 2 — cs4c01290_si_002.zip [file cs4c01290_si_002.zip › vibration animations/Sn(200)/CO2/bound through carbon/0 V_RHE/1098cm-1.gif]

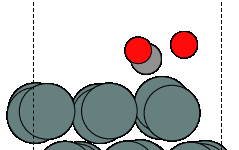

Supplement: Supplementary file 2 — cs4c01290_si_002.zip [file cs4c01290_si_002.zip › vibration animations/Sn(200)/CO2/bound through carbon/0 V_RHE/1489cm-1.gif]

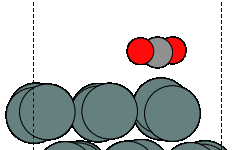

Supplement: Supplementary file 2 — cs4c01290_si_002.zip [file cs4c01290_si_002.zip › vibration animations/Sn(200)/CO2/bound through carbon/-0.5 V_RHE/1151cm-1.gif]

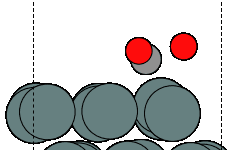

Supplement: Supplementary file 2 — cs4c01290_si_002.zip [file cs4c01290_si_002.zip › vibration animations/Sn(200)/CO2/bound through carbon/-0.5 V_RHE/1461cm-1.gif]

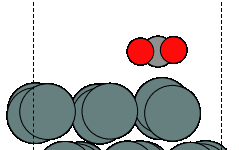

Supplement: Supplementary file 2 — cs4c01290_si_002.zip [file cs4c01290_si_002.zip › vibration animations/Sn(200)/CO2/bound through carbon/-1 V_RHE/1172cm-1.gif]

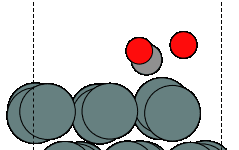

Supplement: Supplementary file 2 — cs4c01290_si_002.zip [file cs4c01290_si_002.zip › vibration animations/Sn(200)/CO2/bound through carbon/-1 V_RHE/1398cm-1.gif]

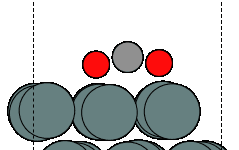

Supplement: Supplementary file 2 — cs4c01290_si_002.zip [file cs4c01290_si_002.zip › vibration animations/Sn(200)/CO2/bound through oxygen(s)/0 V_RHE/1230cm-1.gif]

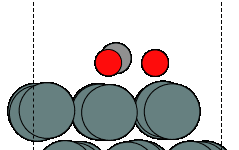

Supplement: Supplementary file 2 — cs4c01290_si_002.zip [file cs4c01290_si_002.zip › vibration animations/Sn(200)/CO2/bound through oxygen(s)/0 V_RHE/2192cm-1.gif]

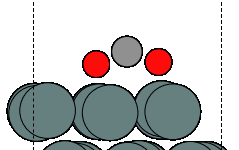

Supplement: Supplementary file 2 — cs4c01290_si_002.zip [file cs4c01290_si_002.zip › vibration animations/Sn(200)/CO2/bound through oxygen(s)/-0.5 V_RHE/1094cm-1.gif]

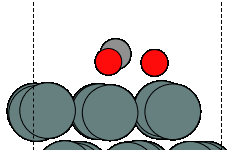

Supplement: Supplementary file 2 — cs4c01290_si_002.zip [file cs4c01290_si_002.zip › vibration animations/Sn(200)/CO2/bound through oxygen(s)/-0.5 V_RHE/1816cm-1.gif]

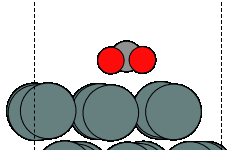

Supplement: Supplementary file 2 — cs4c01290_si_002.zip [file cs4c01290_si_002.zip › vibration animations/Sn(200)/CO2/bound through oxygen(s)/-1 V_RHE/1028cm-1.gif]

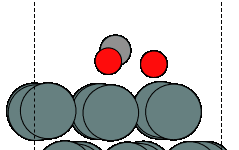

Supplement: Supplementary file 2 — cs4c01290_si_002.zip [file cs4c01290_si_002.zip › vibration animations/Sn(200)/CO2/bound through oxygen(s)/-1 V_RHE/1282cm-1.gif]

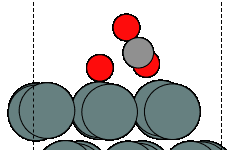

Supplement: Supplementary file 2 — cs4c01290_si_002.zip [file cs4c01290_si_002.zip › vibration animations/Sn(200)/CO3/bidentate/0 V_RHE/1232cm-1.gif]

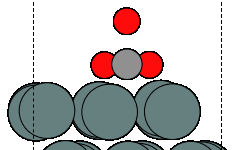

Supplement: Supplementary file 2 — cs4c01290_si_002.zip [file cs4c01290_si_002.zip › vibration animations/Sn(200)/CO3/bidentate/0 V_RHE/1464cm-1.gif]

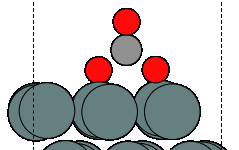

Supplement: Supplementary file 2 — cs4c01290_si_002.zip [file cs4c01290_si_002.zip › vibration animations/Sn(200)/CO3/bidentate/0 V_RHE/999cm-1.gif]

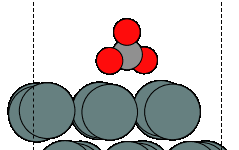

Supplement: Supplementary file 2 — cs4c01290_si_002.zip [file cs4c01290_si_002.zip › vibration animations/Sn(200)/CO3/bidentate/-0.5 V_RHE/1013cm-1.gif]

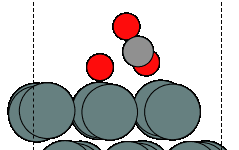

Supplement: Supplementary file 2 — cs4c01290_si_002.zip [file cs4c01290_si_002.zip › vibration animations/Sn(200)/CO3/bidentate/-0.5 V_RHE/1182cm-1.gif]

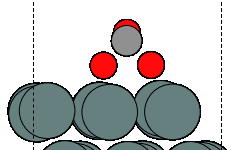

Supplement: Supplementary file 2 — cs4c01290_si_002.zip [file cs4c01290_si_002.zip › vibration animations/Sn(200)/CO3/bidentate/-0.5 V_RHE/1379cm-1.gif]

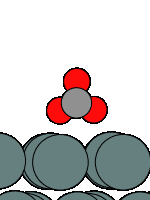

Supplement: Supplementary file 2 — cs4c01290_si_002.zip [file cs4c01290_si_002.zip › vibration animations/Sn(200)/CO3/bidentate/-1 V_RHE/1010cm-1.gif]

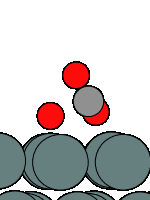

Supplement: Supplementary file 2 — cs4c01290_si_002.zip [file cs4c01290_si_002.zip › vibration animations/Sn(200)/CO3/bidentate/-1 V_RHE/1233cm-1.gif]

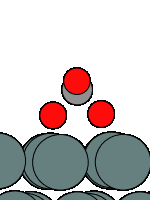

Supplement: Supplementary file 2 — cs4c01290_si_002.zip [file cs4c01290_si_002.zip › vibration animations/Sn(200)/CO3/bidentate/-1 V_RHE/1348cm-1.gif]

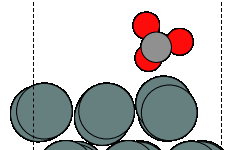

Supplement: Supplementary file 2 — cs4c01290_si_002.zip [file cs4c01290_si_002.zip › vibration animations/Sn(200)/CO3/monodentate/0 V_RHE/1002cm-1.gif]

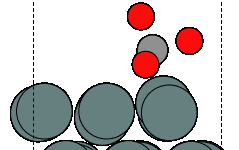

Supplement: Supplementary file 2 — cs4c01290_si_002.zip [file cs4c01290_si_002.zip › vibration animations/Sn(200)/CO3/monodentate/0 V_RHE/1199cm-1.gif]

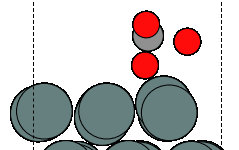

Supplement: Supplementary file 2 — cs4c01290_si_002.zip [file cs4c01290_si_002.zip › vibration animations/Sn(200)/CO3/monodentate/0 V_RHE/1430cm-1.gif]

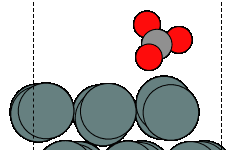

Supplement: Supplementary file 2 — cs4c01290_si_002.zip [file cs4c01290_si_002.zip › vibration animations/Sn(200)/CO3/monodentate/-0.5 V_RHE/1047cm-1.gif]

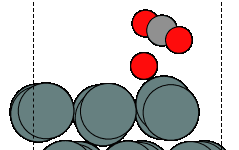

Supplement: Supplementary file 2 — cs4c01290_si_002.zip [file cs4c01290_si_002.zip › vibration animations/Sn(200)/CO3/monodentate/-0.5 V_RHE/1230cm-1.gif]

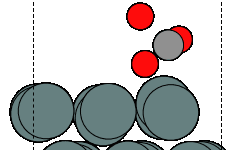

Supplement: Supplementary file 2 — cs4c01290_si_002.zip [file cs4c01290_si_002.zip › vibration animations/Sn(200)/CO3/monodentate/-0.5 V_RHE/1421cm-1.gif]

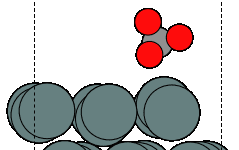

Supplement: Supplementary file 2 — cs4c01290_si_002.zip [file cs4c01290_si_002.zip › vibration animations/Sn(200)/CO3/monodentate/-1 V_RHE/1030cm-1.gif]

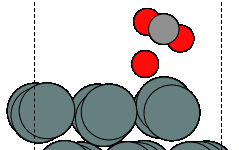

Supplement: Supplementary file 2 — cs4c01290_si_002.zip [file cs4c01290_si_002.zip › vibration animations/Sn(200)/CO3/monodentate/-1 V_RHE/1215cm-1.gif]

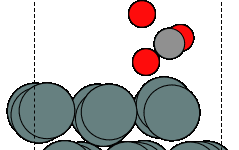

Supplement: Supplementary file 2 — cs4c01290_si_002.zip [file cs4c01290_si_002.zip › vibration animations/Sn(200)/CO3/monodentate/-1 V_RHE/1351cm-1.gif]

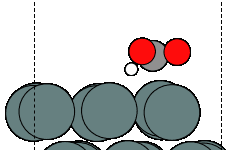

Supplement: Supplementary file 2 — cs4c01290_si_002.zip [file cs4c01290_si_002.zip › vibration animations/Sn(200)/COOH/0 V_RHE/1012cm-1.gif]

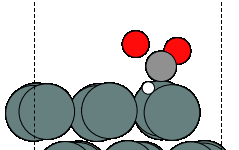

Supplement: Supplementary file 2 — cs4c01290_si_002.zip [file cs4c01290_si_002.zip › vibration animations/Sn(200)/COOH/0 V_RHE/1180cm-1.gif]

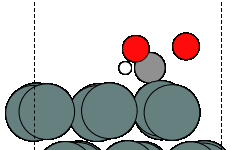

Supplement: Supplementary file 2 — cs4c01290_si_002.zip [file cs4c01290_si_002.zip › vibration animations/Sn(200)/COOH/0 V_RHE/1633cm-1.gif]

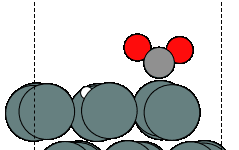

Supplement: Supplementary file 2 — cs4c01290_si_002.zip [file cs4c01290_si_002.zip › vibration animations/Sn(200)/COOH/0 V_RHE/3543cm-1.gif]

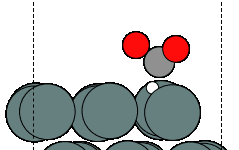

Supplement: Supplementary file 2 — cs4c01290_si_002.zip [file cs4c01290_si_002.zip › vibration animations/Sn(200)/COOH/-0.5 V_RHE/1228cm-1.gif]

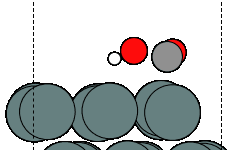

Supplement: Supplementary file 2 — cs4c01290_si_002.zip [file cs4c01290_si_002.zip › vibration animations/Sn(200)/COOH/-0.5 V_RHE/1585cm-1.gif]

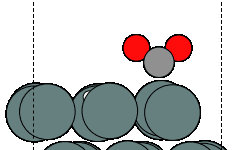

Supplement: Supplementary file 2 — cs4c01290_si_002.zip [file cs4c01290_si_002.zip › vibration animations/Sn(200)/COOH/-0.5 V_RHE/3592cm-1.gif]

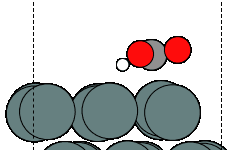

Supplement: Supplementary file 2 — cs4c01290_si_002.zip [file cs4c01290_si_002.zip › vibration animations/Sn(200)/COOH/-0.5 V_RHE/996cm-1.gif]

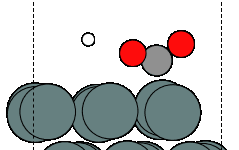

Supplement: Supplementary file 2 — cs4c01290_si_002.zip [file cs4c01290_si_002.zip › vibration animations/Sn(200)/COOH/-1 V_RHE/1158cm-1.gif]

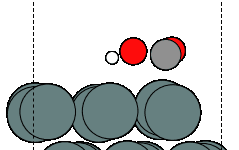

Supplement: Supplementary file 2 — cs4c01290_si_002.zip [file cs4c01290_si_002.zip › vibration animations/Sn(200)/COOH/-1 V_RHE/1577cm-1.gif]

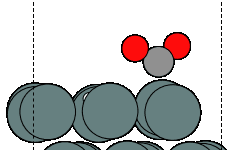

Supplement: Supplementary file 2 — cs4c01290_si_002.zip [file cs4c01290_si_002.zip › vibration animations/Sn(200)/COOH/-1 V_RHE/3411cm-1.gif]

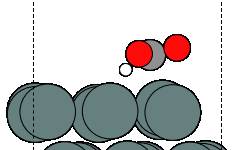

Supplement: Supplementary file 2 — cs4c01290_si_002.zip [file cs4c01290_si_002.zip › vibration animations/Sn(200)/COOH/-1 V_RHE/965cm-1.gif]

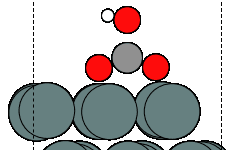

Supplement: Supplementary file 2 — cs4c01290_si_002.zip [file cs4c01290_si_002.zip › vibration animations/Sn(200)/HCO3/bidentate/0 V_RHE/1018cm-1.gif]

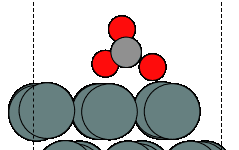

Supplement: Supplementary file 2 — cs4c01290_si_002.zip [file cs4c01290_si_002.zip › vibration animations/Sn(200)/HCO3/bidentate/0 V_RHE/1143cm-1.gif]

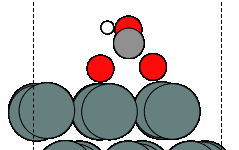

Supplement: Supplementary file 2 — cs4c01290_si_002.zip [file cs4c01290_si_002.zip › vibration animations/Sn(200)/HCO3/bidentate/0 V_RHE/1311cm-1.gif]

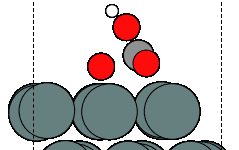

Supplement: Supplementary file 2 — cs4c01290_si_002.zip [file cs4c01290_si_002.zip › vibration animations/Sn(200)/HCO3/bidentate/0 V_RHE/1530cm-1.gif]

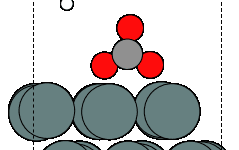

Supplement: Supplementary file 2 — cs4c01290_si_002.zip [file cs4c01290_si_002.zip › vibration animations/Sn(200)/HCO3/bidentate/0 V_RHE/3631cm-1.gif]

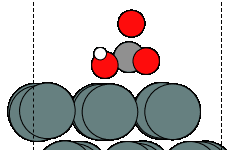

Supplement: Supplementary file 2 — cs4c01290_si_002.zip [file cs4c01290_si_002.zip › vibration animations/Sn(200)/HCO3/bidentate/-0.5 V_RHE/1190cm-1.gif]

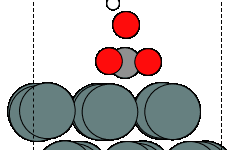

Supplement: Supplementary file 2 — cs4c01290_si_002.zip [file cs4c01290_si_002.zip › vibration animations/Sn(200)/HCO3/bidentate/-0.5 V_RHE/1260cm-1.gif]

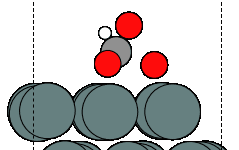

Supplement: Supplementary file 2 — cs4c01290_si_002.zip [file cs4c01290_si_002.zip › vibration animations/Sn(200)/HCO3/bidentate/-0.5 V_RHE/1550cm-1.gif]

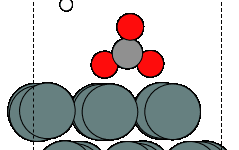

Supplement: Supplementary file 2 — cs4c01290_si_002.zip [file cs4c01290_si_002.zip › vibration animations/Sn(200)/HCO3/bidentate/-0.5 V_RHE/3655cm-1.gif]

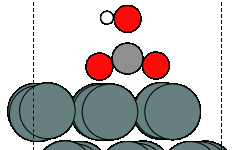

Supplement: Supplementary file 2 — cs4c01290_si_002.zip [file cs4c01290_si_002.zip › vibration animations/Sn(200)/HCO3/bidentate/-0.5 V_RHE/987cm-1.gif]

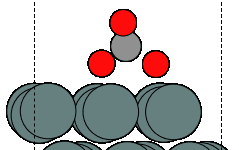

Supplement: Supplementary file 2 — cs4c01290_si_002.zip [file cs4c01290_si_002.zip › vibration animations/Sn(200)/HCO3/bidentate/-1 V_RHE/1204cm-1.gif]

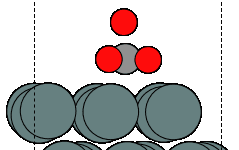

Supplement: Supplementary file 2 — cs4c01290_si_002.zip [file cs4c01290_si_002.zip › vibration animations/Sn(200)/HCO3/bidentate/-1 V_RHE/1261cm-1.gif]

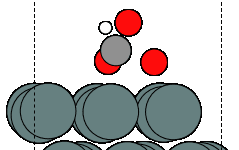

Supplement: Supplementary file 2 — cs4c01290_si_002.zip [file cs4c01290_si_002.zip › vibration animations/Sn(200)/HCO3/bidentate/-1 V_RHE/1541cm-1.gif]

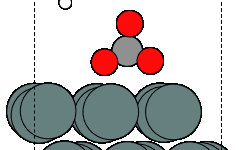

Supplement: Supplementary file 2 — cs4c01290_si_002.zip [file cs4c01290_si_002.zip › vibration animations/Sn(200)/HCO3/bidentate/-1 V_RHE/3676cm-1.gif]

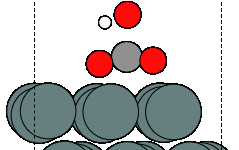

Supplement: Supplementary file 2 — cs4c01290_si_002.zip [file cs4c01290_si_002.zip › vibration animations/Sn(200)/HCO3/bidentate/-1 V_RHE/925cm-1.gif]

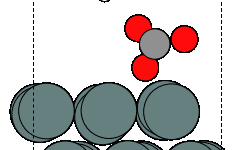

Supplement: Supplementary file 2 — cs4c01290_si_002.zip [file cs4c01290_si_002.zip › vibration animations/Sn(200)/HCO3/monodentate/0 V_RHE/1164cm-1.gif]

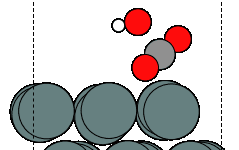

Supplement: Supplementary file 2 — cs4c01290_si_002.zip [file cs4c01290_si_002.zip › vibration animations/Sn(200)/HCO3/monodentate/0 V_RHE/1269cm-1.gif]

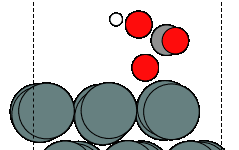

Supplement: Supplementary file 2 — cs4c01290_si_002.zip [file cs4c01290_si_002.zip › vibration animations/Sn(200)/HCO3/monodentate/0 V_RHE/1531cm-1.gif]

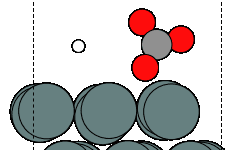

Supplement: Supplementary file 2 — cs4c01290_si_002.zip [file cs4c01290_si_002.zip › vibration animations/Sn(200)/HCO3/monodentate/0 V_RHE/3714cm-1.gif]

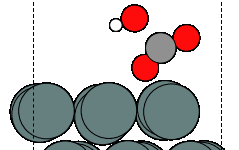

Supplement: Supplementary file 2 — cs4c01290_si_002.zip [file cs4c01290_si_002.zip › vibration animations/Sn(200)/HCO3/monodentate/0 V_RHE/975cm-1.gif]

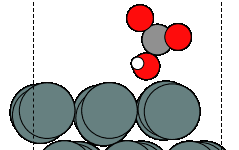

Supplement: Supplementary file 2 — cs4c01290_si_002.zip [file cs4c01290_si_002.zip › vibration animations/Sn(200)/HCO3/monodentate/-0.5 V_RHE/1157cm-1.gif]

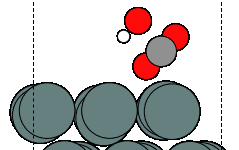

Supplement: Supplementary file 2 — cs4c01290_si_002.zip [file cs4c01290_si_002.zip › vibration animations/Sn(200)/HCO3/monodentate/-0.5 V_RHE/1236cm-1.gif]

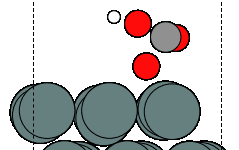

Supplement: Supplementary file 2 — cs4c01290_si_002.zip [file cs4c01290_si_002.zip › vibration animations/Sn(200)/HCO3/monodentate/-0.5 V_RHE/1549cm-1.gif]

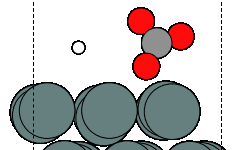

Supplement: Supplementary file 2 — cs4c01290_si_002.zip [file cs4c01290_si_002.zip › vibration animations/Sn(200)/HCO3/monodentate/-0.5 V_RHE/3675cm-1.gif]

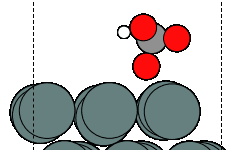

Supplement: Supplementary file 2 — cs4c01290_si_002.zip [file cs4c01290_si_002.zip › vibration animations/Sn(200)/HCO3/monodentate/-0.5 V_RHE/954cm-1.gif]

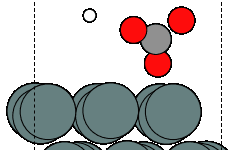

Supplement: Supplementary file 2 — cs4c01290_si_002.zip [file cs4c01290_si_002.zip › vibration animations/Sn(200)/HCO3/monodentate/-1 V_RHE/1113cm-1.gif]

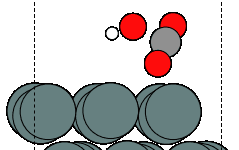

Supplement: Supplementary file 2 — cs4c01290_si_002.zip [file cs4c01290_si_002.zip › vibration animations/Sn(200)/HCO3/monodentate/-1 V_RHE/1257cm-1.gif]

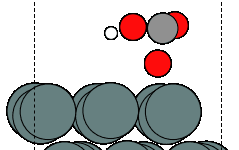

Supplement: Supplementary file 2 — cs4c01290_si_002.zip [file cs4c01290_si_002.zip › vibration animations/Sn(200)/HCO3/monodentate/-1 V_RHE/1575cm-1.gif]

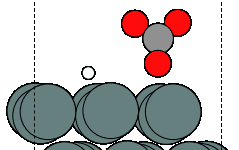

Supplement: Supplementary file 2 — cs4c01290_si_002.zip [file cs4c01290_si_002.zip › vibration animations/Sn(200)/HCO3/monodentate/-1 V_RHE/3773cm-1.gif]

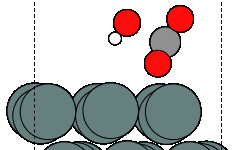

Supplement: Supplementary file 2 — cs4c01290_si_002.zip [file cs4c01290_si_002.zip › vibration animations/Sn(200)/HCO3/monodentate/-1 V_RHE/877cm-1.gif]

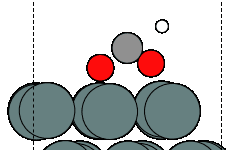

Supplement: Supplementary file 2 — cs4c01290_si_002.zip [file cs4c01290_si_002.zip › vibration animations/Sn(200)/OCHO/bidentate/0 V_RHE/1265cm-1.gif]

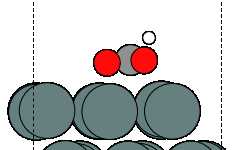

Supplement: Supplementary file 2 — cs4c01290_si_002.zip [file cs4c01290_si_002.zip › vibration animations/Sn(200)/OCHO/bidentate/0 V_RHE/1334cm-1.gif]

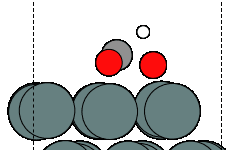

Supplement: Supplementary file 2 — cs4c01290_si_002.zip [file cs4c01290_si_002.zip › vibration animations/Sn(200)/OCHO/bidentate/0 V_RHE/1513cm-1.gif]

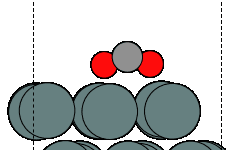

Supplement: Supplementary file 2 — cs4c01290_si_002.zip [file cs4c01290_si_002.zip › vibration animations/Sn(200)/OCHO/bidentate/0 V_RHE/2963cm-1.gif]

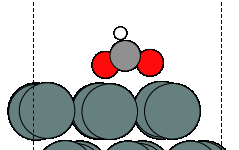

Supplement: Supplementary file 2 — cs4c01290_si_002.zip [file cs4c01290_si_002.zip › vibration animations/Sn(200)/OCHO/bidentate/0 V_RHE/988cm-1.gif]

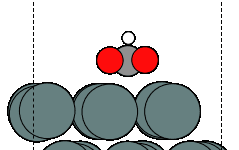

Supplement: Supplementary file 2 — cs4c01290_si_002.zip [file cs4c01290_si_002.zip › vibration animations/Sn(200)/OCHO/bidentate/-0.5 V_RHE/1293cm-1.gif]

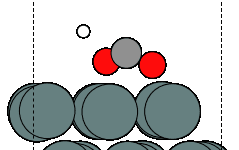

Supplement: Supplementary file 2 — cs4c01290_si_002.zip [file cs4c01290_si_002.zip › vibration animations/Sn(200)/OCHO/bidentate/-0.5 V_RHE/1311cm-1.gif]

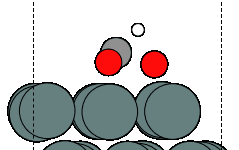

Supplement: Supplementary file 2 — cs4c01290_si_002.zip [file cs4c01290_si_002.zip › vibration animations/Sn(200)/OCHO/bidentate/-0.5 V_RHE/1478cm-1.gif]

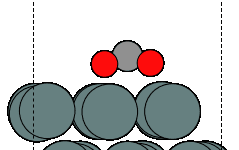

Supplement: Supplementary file 2 — cs4c01290_si_002.zip [file cs4c01290_si_002.zip › vibration animations/Sn(200)/OCHO/bidentate/-0.5 V_RHE/2956cm-1.gif]

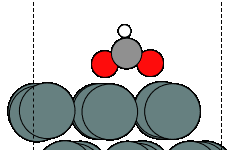

Supplement: Supplementary file 2 — cs4c01290_si_002.zip [file cs4c01290_si_002.zip › vibration animations/Sn(200)/OCHO/bidentate/-0.5 V_RHE/967cm-1.gif]

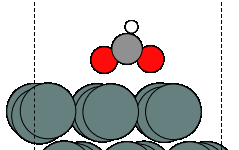

Supplement: Supplementary file 2 — cs4c01290_si_002.zip [file cs4c01290_si_002.zip › vibration animations/Sn(200)/OCHO/bidentate/-1 V_RHE/1110cm-1.gif]

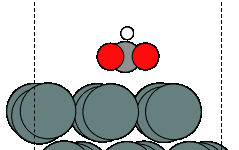

Supplement: Supplementary file 2 — cs4c01290_si_002.zip [file cs4c01290_si_002.zip › vibration animations/Sn(200)/OCHO/bidentate/-1 V_RHE/1267cm-1.gif]

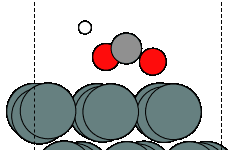

Supplement: Supplementary file 2 — cs4c01290_si_002.zip [file cs4c01290_si_002.zip › vibration animations/Sn(200)/OCHO/bidentate/-1 V_RHE/1308cm-1.gif]

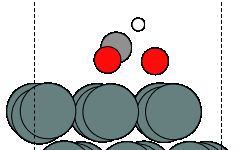

Supplement: Supplementary file 2 — cs4c01290_si_002.zip [file cs4c01290_si_002.zip › vibration animations/Sn(200)/OCHO/bidentate/-1 V_RHE/1443cm-1.gif]

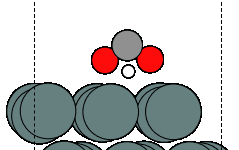

Supplement: Supplementary file 2 — cs4c01290_si_002.zip [file cs4c01290_si_002.zip › vibration animations/Sn(200)/OCHO/bidentate/-1 V_RHE/2831cm-1.gif]

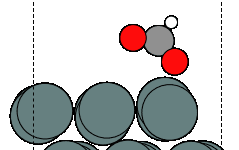

Supplement: Supplementary file 2 — cs4c01290_si_002.zip [file cs4c01290_si_002.zip › vibration animations/Sn(200)/OCHO/monodentate/0 V_RHE/1017cm-1.gif]

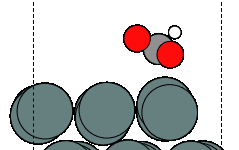

Supplement: Supplementary file 2 — cs4c01290_si_002.zip [file cs4c01290_si_002.zip › vibration animations/Sn(200)/OCHO/monodentate/0 V_RHE/1274cm-1.gif]

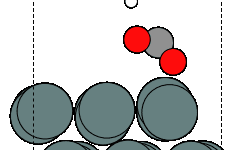

Supplement: Supplementary file 2 — cs4c01290_si_002.zip [file cs4c01290_si_002.zip › vibration animations/Sn(200)/OCHO/monodentate/0 V_RHE/1343cm-1.gif]

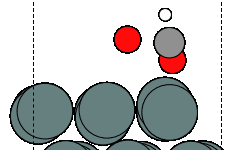

Supplement: Supplementary file 2 — cs4c01290_si_002.zip [file cs4c01290_si_002.zip › vibration animations/Sn(200)/OCHO/monodentate/0 V_RHE/1502cm-1.gif]

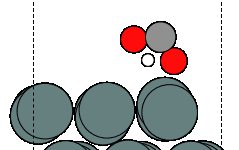

Supplement: Supplementary file 2 — cs4c01290_si_002.zip [file cs4c01290_si_002.zip › vibration animations/Sn(200)/OCHO/monodentate/0 V_RHE/2900cm-1.gif]

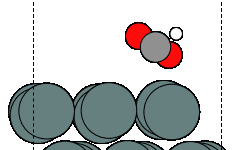

Supplement: Supplementary file 2 — cs4c01290_si_002.zip [file cs4c01290_si_002.zip › vibration animations/Sn(200)/OCHO/monodentate/-0.5 V_RHE/1295cm-1.gif]

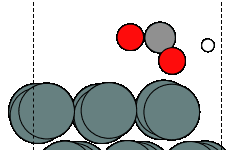

Supplement: Supplementary file 2 — cs4c01290_si_002.zip [file cs4c01290_si_002.zip › vibration animations/Sn(200)/OCHO/monodentate/-0.5 V_RHE/1341cm-1.gif]

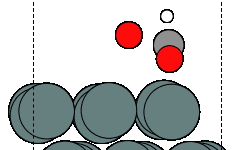

Supplement: Supplementary file 2 — cs4c01290_si_002.zip [file cs4c01290_si_002.zip › vibration animations/Sn(200)/OCHO/monodentate/-0.5 V_RHE/1492cm-1.gif]

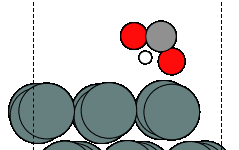

Supplement: Supplementary file 2 — cs4c01290_si_002.zip [file cs4c01290_si_002.zip › vibration animations/Sn(200)/OCHO/monodentate/-0.5 V_RHE/2956cm-1.gif]

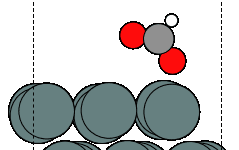

Supplement: Supplementary file 2 — cs4c01290_si_002.zip [file cs4c01290_si_002.zip › vibration animations/Sn(200)/OCHO/monodentate/-0.5 V_RHE/978cm-1.gif]

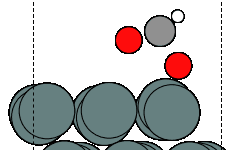

Supplement: Supplementary file 2 — cs4c01290_si_002.zip [file cs4c01290_si_002.zip › vibration animations/Sn(200)/OCHO/monodentate/-1 V_RHE/1297cm-1.gif]

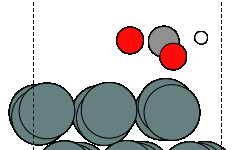

Supplement: Supplementary file 2 — cs4c01290_si_002.zip [file cs4c01290_si_002.zip › vibration animations/Sn(200)/OCHO/monodentate/-1 V_RHE/1415cm-1.gif]

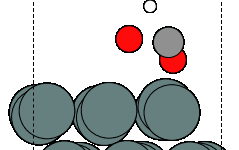

Supplement: Supplementary file 2 — cs4c01290_si_002.zip [file cs4c01290_si_002.zip › vibration animations/Sn(200)/OCHO/monodentate/-1 V_RHE/1482cm-1.gif]
